# Screening of Self-Assembling of Collagen IV Fragments into Stable Structures Potentially Useful in Regenerative Medicine

**DOI:** 10.3390/ijms222413584

**Published:** 2021-12-18

**Authors:** Marcin Kolasa, Grzegorz Galita, Ireneusz Majsterek, Ewa Kucharska, Katarzyna Czerczak, Joanna Wasko, Angelika Becht, Justyna Fraczyk, Anna Gajda, Lukasz Pietrzak, Lukasz Szymanski, Agnieszka Krakowiak, Zbigniew Draczynski, Beata Kolesinska

**Affiliations:** 1 General Command of the Polish Armed Forces, Medical Division, Zwirki i Wigury 103/105, 00-912 Warsaw, Poland; makolasa@ron.mil.pl; 2 Department of Clinical Chemistry and Biochemistry, Medical University of Lodz, Narutowicza 60, 90-136 Lodz, Poland; grzegorz.galita@umed.lodz.pl (G.G.); ireneusz.majsterek@umed.lodz.pl (I.M.); 3 Department Geriatrics and Social Work, Jesuit University Ignatianum in Cracow, Kopernika 26, 31-501 Krakow, Poland; ewa.kucharska@ignatianum.edu.pl; 4 Institute of Organic Chemistry, Faculty of Chemistry, Lodz University of Technology, Zeromskiego 116, 90-924 Lodz, Poland; katarzyna.czerczak@dokt.p.lodz.pl (K.C.); joanna.wasko@p.lodz.pl (J.W.); anglika.becht@dokt.p.lodz.pl (A.B.); justyna.fraczyk@p.lodz.pl (J.F.); anna.gajda@p.lodz.pl (A.G.); 5 Institute of Mechatronics and Information Systems, Faculty of Electrical, Electronic, Computer and Control Engineering, Lodz University of Technology, Stefanowskiego 18/22, 90-924 Lodz, Poland; lukasz.pietrzak@p.lodz.pl (L.P.); lukasz.szymanski@p.lodz.pl (L.S.); 6 Centre of Molecular and Macromolecular Studies Polish Academy of Sciences, Department of Bioorganic Chemistry, Sienkiewicza 112, 90-363 Lodz, Poland; akrakow@cbmm.lodz.pl; 7 Institute of Material Sciences of Textiles and Polymer Composites, Faculty of Material Technologies and Textile Design, Lodz University of Technology, Zeromskiego 116, 90-924 Lodz, Poland; zbigniew.draczynski@p.lodz.pl

**Keywords:** collagen IV, basement membrane, self-assembling, ability to mimic native collagen structures, porous material, solid phase peptide synthesis, triazine coupling reagent

## Abstract

The aim of the research was to check whether it is possible to use fragments of type IV collagen to obtain, as a result of self-assembling, stable spatial structures that could be used to prepare new materials useful in regenerative medicine. Collagen IV fragments were obtained by using DMT/NMM/TosO^−^ as a coupling reagent. The ability to self-organize and form stable spatial structures was tested by the CD method and microscopic techniques. Biological studies covered: resazurin assay (cytotoxicity assessment) on BJ, BJ-5TA and C2C12 cell lines; an alkaline version of the comet assay (genotoxicity), Biolegend Legendplex human inflammation panel 1 assay (SC cell lines, assessment of the inflammation activity) and MTT test to determine the cytotoxicity of the porous materials based on collagen IV fragments. It was found that out of the pool of 37 fragments (peptides **1**–**33** and **2.1**–**2.4**) reconstructing the outer sphere of collagen IV, nine fragments (peptides: **2**, **4**, **5**, **6**, **14**, **15**, **25**, **26** and **30**), as a result of self-assembling, form structures mimicking the structure of the triple helix of native collagens. The stability of spatial structures formed as a result of self-organization at temperatures of 4 °C, 20 °C, and 40 °C was found. The application of the MST method allowed us to determine the K_d_ of binding of selected fragments of collagen IV to ITGα1β1. The stability of the spatial structures of selected peptides made it possible to obtain porous materials based on their equimolar mixture. The formation of the porous materials was found for cross-linked structures and the material stabilized only by weak interactions. All tested peptides are non-cytotoxic against all tested cell lines. Selected peptides also showed no genotoxicity and no induction of immune system responses. Research on the use of porous materials based on fragments of type IV collagen, able to form stable spatial structures as scaffolds useful in regenerative medicine, will be continued.

## 1. Introduction

Regenerative medicine concerns not only the healing of tissue after an injury but also restoring damaged tissues to their native function. The development of biomaterials useful in regenerative medicine has made it possible to obtain new medical materials that not only do not induce cellular system responses but also stimulate tissue regeneration and restore their original functionality. In addition, they allow communication at the cellular level [1,2]. The materials and methods used to make new scaffolds are very diverse. However, the most promising is the use of natural compounds that reflect the natural environment of cells as much as possible. From the pool of biopolymers, collagen is the most used because it is the main building block of the extracellular matrix (ECM). Collagen materials have been used in the regeneration of osteochondral defects [3], connective tissue [4], adipose tissue [5,6], mammary glands [7] and others [8,9,10,11,12]. In addition, in the case of scaffolds based on natural materials, including collagens [13], the pore architecture can be designed to mimic the anisotropic nature of ECM in native tissues [14,15]. Moreover, isotropic scaffolds can be prepared with pores ranging in size from 90 to 300 mm. This parameter influences the growth and mechanics of cells. It is also possible to obtain materials with a defined diameter percolation, which affects cell infiltration [16,17]. Other biopolymers are used as modifiers of the biological activity of collagen materials, e.g., chitosan, alginate, elastin, the presence of which affects the chemical and mechanical properties of scaffolds [18,19]. Other components of the ECM are also used as additives [20,21,22,23].

The cellular response to the biomaterials used for the preparation of scaffolds is the result of mechanical, architectural and chemical factors. Receiving signals and transmitting them into and out of the cell is a complex and highly dynamic process [24,25,26,27]. The mechanism of signal transmission to and from cells is related to transmembrane receptors, in which integrins play an important role, and control many cell functions, including differentiation [28,29]. For tissue engineering, tailoring a biological response results from the use of ligands or signaling molecules that are presented to cells and interact with receptors. The ECM is a dynamic three-dimensional structure that anchors and surrounds cells. The ECM consists of interacting macromolecules, creating an interstitial matrix, basement membrane and mineralized matrix. The ECM determines the structural integrity of organs and tissues during development and homeostasis. It is also the main determinant of the fate and survival of the cell [30]. Cells synthesize huge amounts (~300) of various ECM proteins, which are termed the “core matrisome”. The main components of the ECM are: (1) collagens; (2) elastin; (3) glycoproteins; and (4) proteoglycans. In addition, there are ECM-related proteins, including growth factors and enzymes involved in protein degradation and modification (e.g., cross-linking enzymes). Collagens and elastin form insoluble cross-linked supra-structures that resist tensile forces and impart elasticity to tissues. The proteoglycans embedded in these supra-structures and the associated glycosaminoglycans moisturize the matrix, provide turgor and regulate the diffusion of nutrients, metabolites and hormones. Glycoproteins such as fibronectin and laminins form multimeric assemblies and provide connections between cells and the ECM by interacting with other supra-structures, growth factors, cytokines, and membrane receptors. Collagens are the main structural components of connective tissue and the most common protein family in animals, making up about 30% of the total protein mass [31]. Collagens self-organize into structurally and functionally different multimeric assemblies [32]. These diverse supra-structures give tissues tensile strength and form a scaffold that protects them from mechanical stress [33]. The interaction between collagens and cells mediates cell adhesion and motility during tissue morphogenesis, growth and wound healing. Collagen molecules are trimeric and can be homo- or heterotrimeric. The collagen superfamily is characterized by the presence of a COL domain that contains a repeated tripeptide sequence containing glycine and proline residues. The COL domain forms a triple helix in any system containing three α chains. Collagens also contain a number of non-collagen (NC) domains that are involved in a wide spectrum of interactions with other matrix proteins and cells. Collagens I, II, III, V, XI, XXIV, and XXVII are fibril-forming proteins and contain long, uninterrupted COL domains terminating in small globular NC domains. The fibril-associated collagens (FACIT), characterized by a discontinuous triple helix structure, include collagens type IX, XII, XIV, and XX. Their characteristic feature is the presence of a tandem repeating short COL domains interfered by NC regions. Collagen types IV, VI, VIII, and X are a network-forming group of collagens [34]. Collagen IV is the main component of the basement membrane (also defined as a basement membrane zone (BMZ)) and is crucial for structural maintenance [35]. Although six collagen IV α-chains are encoded, only three heterotrimeric structures are presented in tissues [36]. The α-chains of IV collagen have similar domain structures and 50–70% amino acid homology. The collagen IV α-chains include three domains: a short N-terminal 7S domain, a central triple-helical (COL domain) and a non-collagenous 1 (NC1) domain at C-end [37,38]. The triple-helical domain includes interrupted G-X-Y repeats. The 7S and NC1 domains are responsible for assembling collagen IV into a three-dimensional structure conditioning both stiffness and flexibility due to numerous Gly-X-Y interruptions [39]. The collagen IV type network participates in cell adhesion by the integrin, CD44 receptor, transmembrane collagen receptor and discoidin domain receptor 1. Collagen type IV is connected with laminin network through nidogens and gives mechanical stability to the structure [40]. The interactions of NC1 domain with α1β1 integrin inhibit signaling pathways in vascular epithelial cells via integrins. Collagen type IV also regulates HIF-1α and VEGF expression through inhibition of the MAPK signaling cascade, which is associated with the antitumorigenic activity of NC1 domain [41].

Since the basement membrane interacts with various cells such as epithelial, endothelial, fat and smooth muscle cells, its components become extremely useful from the point of view of wide application in medicine [42,43,44,45,46]. However, due to the fact that the basement membrane extracellular matrix is too thin for direct use, a method has been developed to obtain materials containing all of the components of the basement membrane and those which have retained biological activity. This extract is known as Matrigel, Cultrex, or EHS matrix and it stimulates cell differentiation. In vivo, this material is used for repairing damaged tissues and studying angiogenic inhibitors and stimulators [47,48].

The aim of the research was to check whether it is possible to use fragments of type IV collagen to obtain, as a result of self-assembling, stable spatial structures that could be used to prepare new materials useful in regenerative medicine. It can be expected that the use of the set of fragments forming the outer sphere in place of the native collagen IV (which, unfortunately, is also associated with the Alport syndrome and Goodpasture syndrome) should guarantee both the proper spatial structure formed as a result of self-organization and ensure interaction with cells and other components of the basal membrane. It was assumed that at the stage of selecting collagen IV fragments, their susceptibility to self-organization with the use of various techniques will be tested and that their safety and usefulness will be assessed on both fibroblasts and muscle cells.

## 2. Results and Discussion

Collagen peptides produced by the proteolytic cleavage of collagen were considered useless. In recent years, however, it has been shown that they enhance and regulate various cellular processes mainly involving integrins. Many collagen peptides have been identified from many different types of collagen capable of regulating processes such as cell proliferation, migration, apoptosis, and reduction of angiogenesis. The most interesting collagen peptides are generated from the primary collagen of the tissue interstitial matrix, type I collagen and the basement membrane, type IV collagen [49]. However, the process of enzymatic degradation only takes place with the use of a selected group of enzymes. Additionally, large polypeptides are formed as a result of enzymatic and chemical degradation of collagens [50].

On the other hand, the so-called Collagen Mimetic Peptides (CMP), which are used as a molecular tool for collagen research and as building blocks for the development of collagen biomaterial mimetics, are known. The uses of the CMP have made it possible to deepen the understanding of the structure and molecular properties of collagen triple helix and the interaction of collagen with molecular ligands. CMP has been used to create heterotrimeric CMP derivatives and CMP-based collagen-like materials [51].

In our research, we made attempts to use the fragments of collagen IV forming the outer sphere of the native protein to obtain new porous materials and their initial evaluation (lack of cytotoxicity and the ability to proliferate cells). However, the commencement of this stage of research required: (1) checking the safety of collagen peptides, (2) testing the self-assembly ability of collagen IV fragments to create permanent spatial structures characteristic of collagen IV.

### 2.1. Synthesis of Collagen IV Fragments Reconstructing the Inner Sphere of the Native Protein

**Figure 1:**
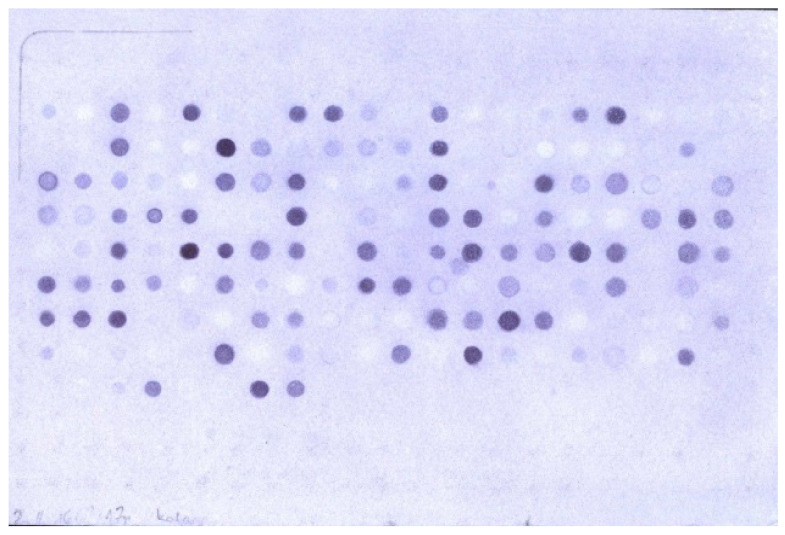
Stained immune complexes of immobilized collagen IV fragments with antibodies.

A pool of collagen fragments IV (CO4A6_HUMAN Q14031 Collagen alpha-6(IV) chain) was used for the tests. These fragments positively interacted with polyclonal antibodies against the human collagen alpha-6 (IV) chain in a dot blot test with a library of cellulose-immobilized collagen IV fragments (in the first screening, a library of non-overlapping deca-amino acid fragments of collagen IV was obtained). The scan of the cellulose immobilized library of non-overlapping fragments of collage IV after the Dot blot test is shown in Figure 1.

The set of fragments selected in this way forms the outer sphere of the native protein [52]. The reconstructed outer sphere of a protein determines the ability to interact with other proteins or molecules because fragments properly exposed to the outside are involved in the protein-protein and peptide-protein interactions [53,54,55]. As part of the research, 33 peptides are planned to be tested (peptides **1**–**33**) selected in the first screening. Mapping (the reading frame shifted by five amino acid residues towards the N and C-terminus) allowed for the selection of additional four peptides (peptides **2.1**–**2.4**) selected in the second screening [52]. Peptides **2.1**–**2.4** derived from peptides **1**, **31**, **32** selected in the first screening. For the synthesis of collagen IV fragments, the classic solid-phase method was used. As a coupling reagent 4-(4,6-dimethoxy-1,3,5-triazin-2-yl)-4-methylmorpholinium toluene-4-sulfonate (DMT/NMM/TosO^−^) [56] was used. Synthesized fragments of collagen IV are summarized in Table 1.

Additionally, 4 fragments (peptides **2.1**–**2.4**) (Table 2) were synthesized, the structures of which were selected in the second screening using the dot-blot technique [49].

### 2.2. Research on Biological Activity of Collagen IV Fragments

**Figure 2:**
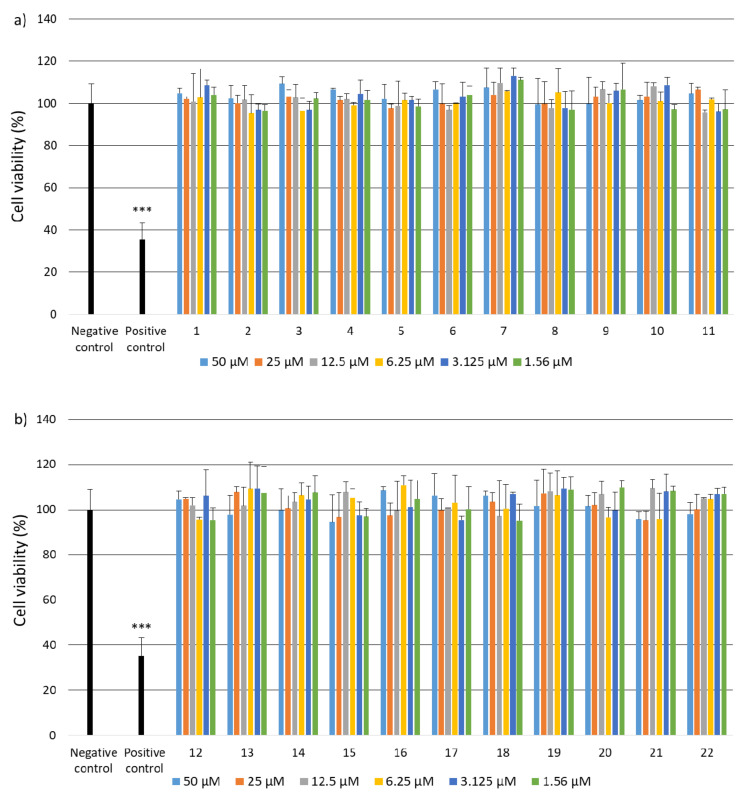

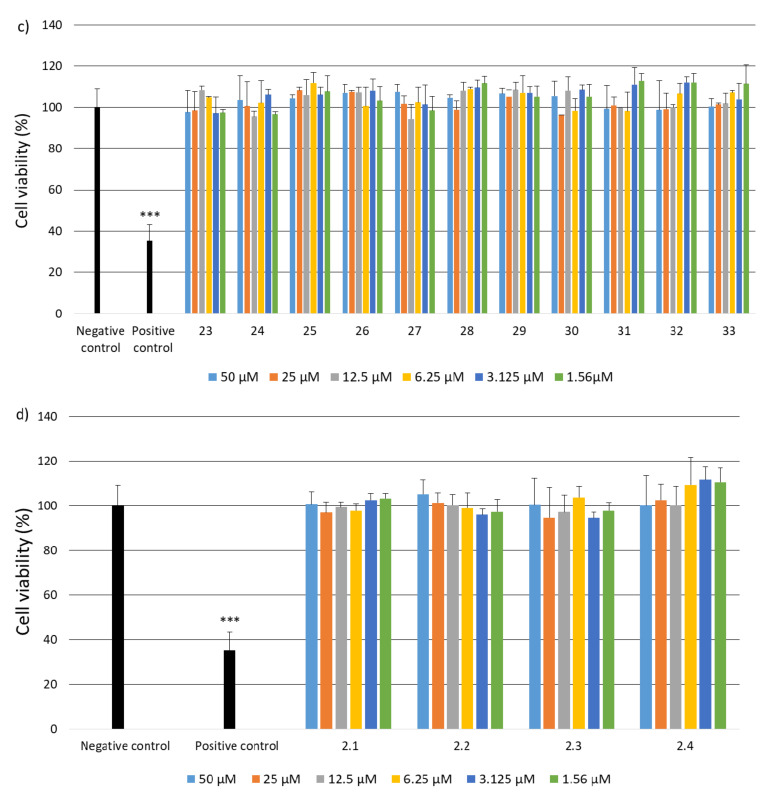
Analysis of the cytotoxicity effect (at different concentrations) of fragments of collagen IV on BJ cell lines. (**a**) peptides **1**–**11**, (**b**) peptides **12**–**22**, (**c**) peptides **23**–**33**, (**d**) peptides **2.1**–**2.4**. The in vitro toxicology resazurin assay was used to assess the cytotoxicity of the tested compounds. All of the experiments were performed in triplicate. Data are expressed as mean ± SD (*n* = 3), *** *p* < 0.001 versus the negative control.

**Figure 3:**
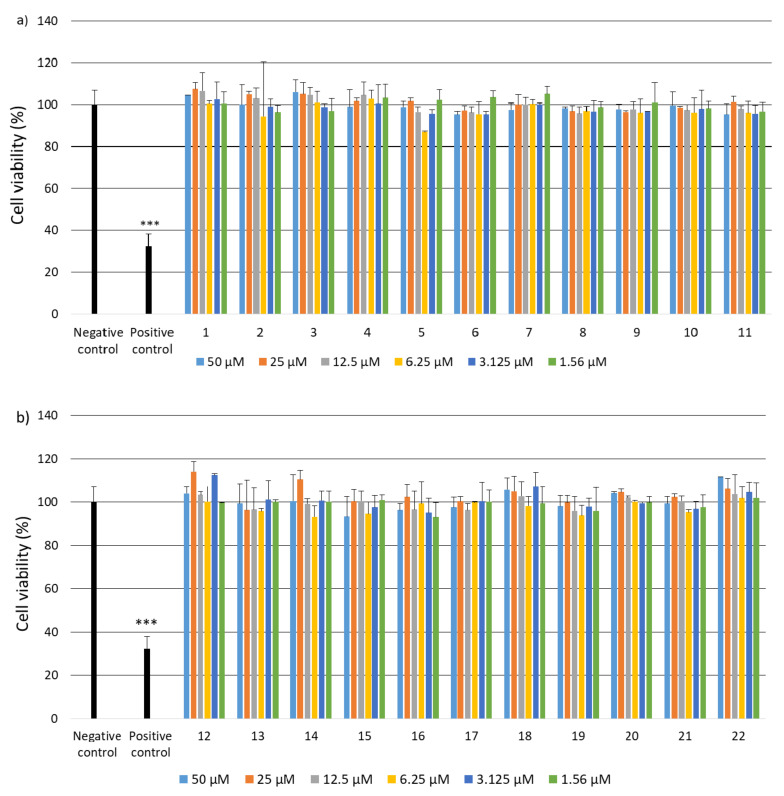

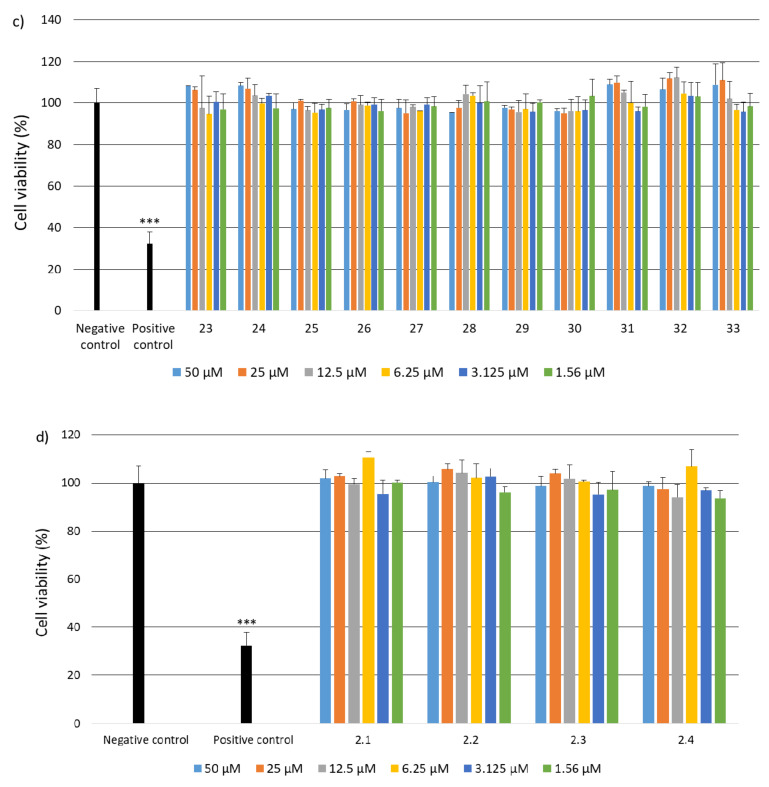
Analysis of the cytotoxicity effect (at different concentrations) of fragments of collagen IV on BJ-5ta cell lines. (**a**) peptides **1**–**11**, (**b**) peptides **12**–**22**, (**c**) peptides **23**–3**3**, (**d**) peptides **2.1**–**2.4**. The in vitro toxicology assay resazurin was used to assess the cytotoxicity of the tested compounds. All of the experiments were performed in triplicate. Data are ex-pressed as mean ± SD (*n* = 3), *** *p* < 0.001 versus the negative control.

**Figure 4:**
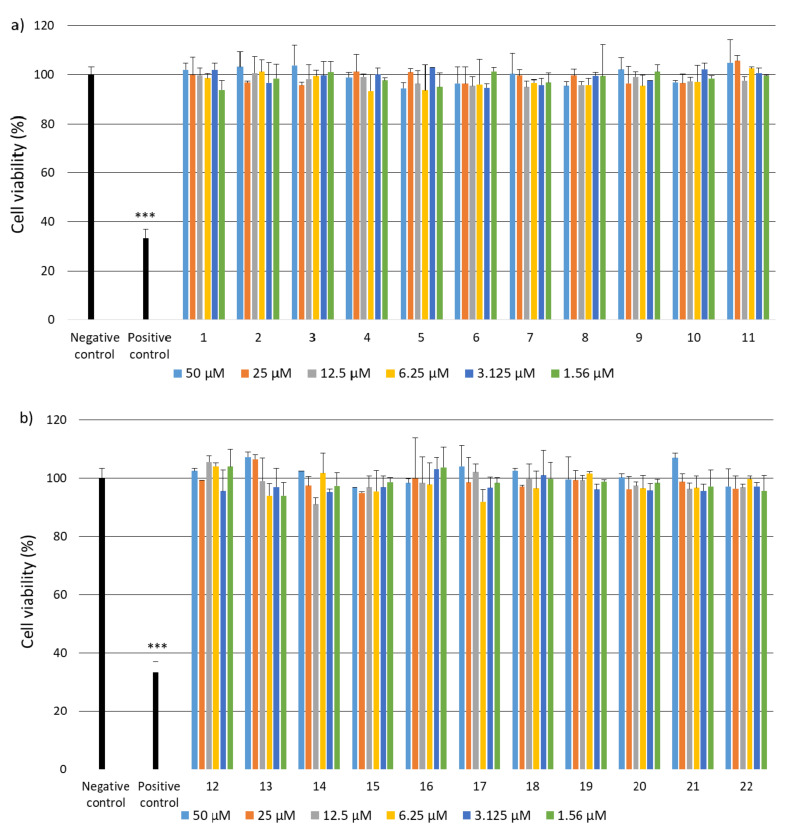

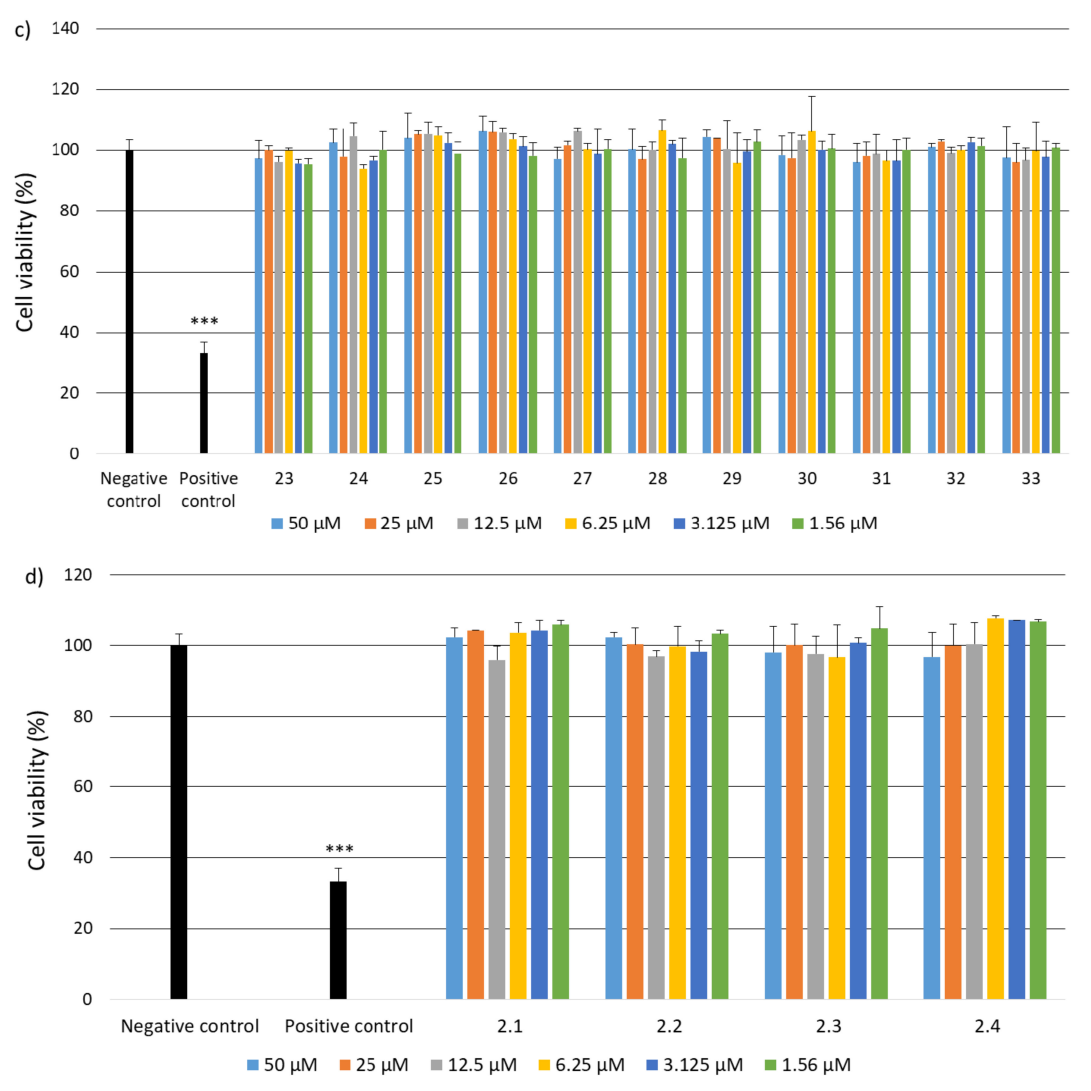
Analysis of the cytotoxicity effect (at different concentrations) of fragments of collagen IV on C2C12 cell lines. (**a**) peptides **1**–**11**, (**b**) peptides **12**–**22**, (**c**) peptides **23**–**33**, (**d**) peptides **2.1**–**2.4**. The in vitro toxicology assay resazurin was used to assess the cytotoxicity of the tested compounds. All of the experiments were performed in triplicate. Data are ex-pressed as mean ± SD (*n* = 3), *** *p* < 0.001 versus the negative control.

Due to the fact that the selected fragments of collagen IV reconstructing the external sphere of the native protein, it was planned to use them to obtain porous materials useful as a scaffold in regenerative medicine, it was necessary to check their safety. Despite the fact that they are a fragment of a native human protein, these studies were necessary because many examples of the cytotoxic activity of peptides are known. A flagship example of this is the cytotoxicity of protein fragments, which as a result of self-assembly form hot spots for the protein aggregation process leading to the formation of insoluble deposits. A characteristic feature of these peptides is the β-sheet structure. It is also known that the dissolved oligomeric aggregates are often more cytotoxic [57] than the finally formed insoluble aggregates. The cytotoxicity of peptides with the f β—hairpin structure is also well documented [58]. Therefore, in the first stage of the study, it was examined whether the selected peptides did not exhibit cytotoxicity in the BJ, BJ-5TA and C2C12 cell lines (Figure 2, Figure 3 and Figure 4). The use of skin (human, mouse) and muscle cell lines resulted from the potential use of materials derived from collagen IV fragments in regenerative medicine. For the evaluation of cytotoxicity, resazurin-based assay kit (resazurin-based assay belongs to the colorimetric tests) was used. The amount of red intermediate pigment (resorufin) formed as a result of resazurin reduction is directly proportional to the number of viable cells. The cytotoxicity of peptides **1**–**33** and **2.1**–**2.4** was tested at concentrations of 50, 25, 13.5, 6.25, 1.125 and 1.56 μM. Cells cultured in a complete growth medium were used as a negative control, whereas cells incubated with 100% DMSO constituted a positive control. All of the analyzed samples and controls were incubated for 48 h.

The obtained results show that each of the tested collagen IV fragments did not exhibit cytotoxic properties at any concentrations. In each test sample, the level of cell viability after exposure to collagen oligopeptides did not fall below 93%. The results are identical to those obtained for the negative control, which was the incubation of cells with the medium only. Moreover, for almost all the tested peptides, even a slightly higher level of cell viability can be observed compared to the negative control, which proves that the potential of cells to proliferate is preserved in the presence of collagen IV fragments.

**Figure 5:**
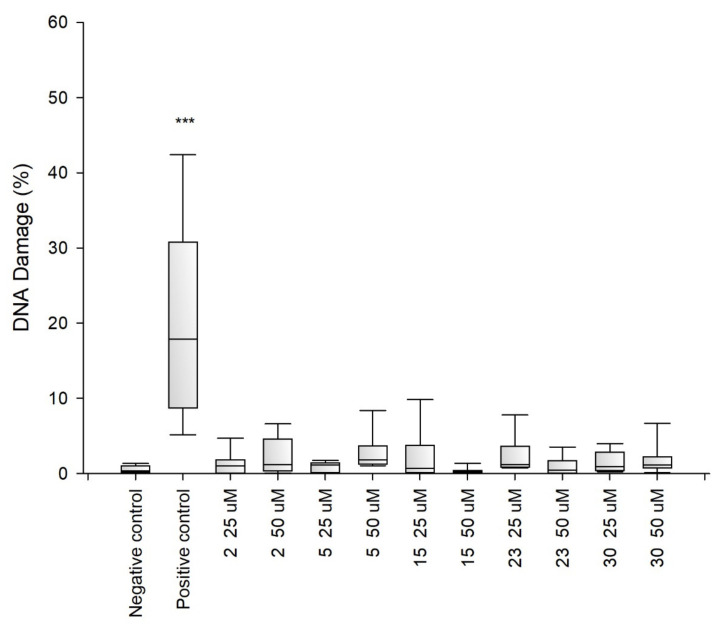
Analysis of genotoxicity of the selected fragments of collagen IV on BJ cell lines. Statistical analysis was based on the results of three independent tests. The differences were statistically significant as follows: *** *p* < 0.001 versus the negative control.

**Figure 6:**
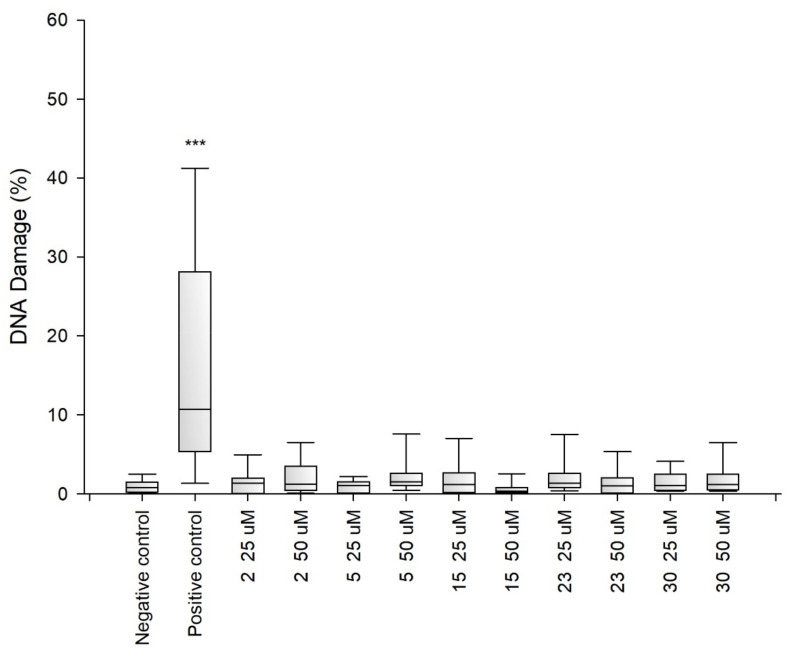
Analysis of genotoxicity of the selected fragments of collagen IV on BJ-5ta cell lines. Statistical analysis was based on the results of three independent tests. The differences were statistically significant as follows: *** *p* < 0.001 versus the negative control.

**Figure 7:**
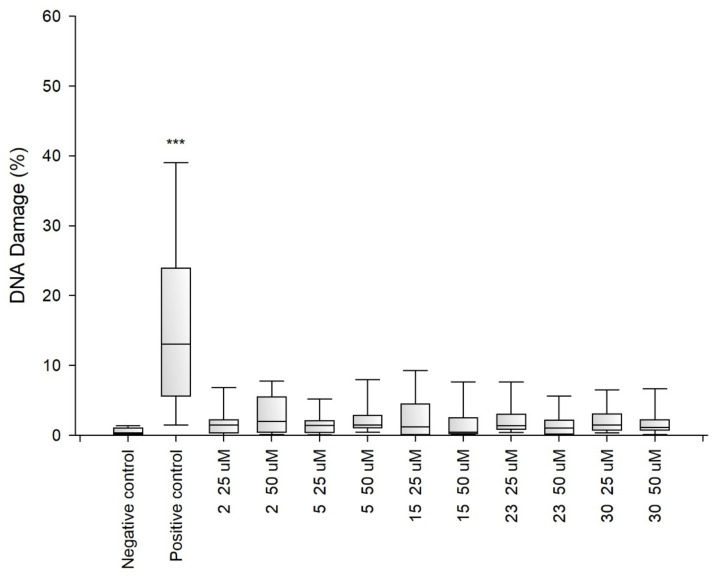
Analysis of genotoxicity of the selected fragments of collagen IV on C2C12 cell lines. Statistical analysis was based on the results of three independent tests. The differences were statistically significant as follows: *** *p* < 0.001 versus the negative control.

Genotoxicity assessment is part of the safety assessment of all types of substances. Compounds identified as genotoxic are considered carcinogenic and/or mutagenic and may cause cancer and/or genetic defects. Testing the genotoxicity of new chemicals is an integral part of the development of new compounds for medical use and is a regulatory requirement [59,60,61]. Therefore, for selected peptides ^41^CFPEKGARGR^50^ (**2**), ^221^GFQGEKGVKG^230^ (**5**) ^721^LPGFPGLPGK^730^ (**15**), ^961^MSNLWLKGDK^970^ (**23**) and ^1331^DPGFPGMKGK^1340^ (**30**), tests were performed to determine their genotoxicity (Figure 5, Figure 6 and Figure 7). The study assessed the genotoxicity of the peptides **2**, **5**, **15**, **23** and **30** at concentrations of 25 and 50 μM.

The alkaline version of the comet assay was used to assess the level of DNA damage evoked by the tested variants of chitosan gels. The amount of DNA damage was estimated from the percentage of DNA in the comet tail. The obtained results showed that at both concentrations tested collagen IV fragments did not induce significant DNA damage in BJ, BJ-5TA and C2C12 cells.

Standardized in vitro test protocols for the evaluation of new biomaterials are generally compliant with the Protocols of the International Organization for Standardization (ISO) [62] and they mainly relate to the cytotoxic effect on various immortalized cell lines, primary cells, stem cells essential for the target tissue. Additionally, tests are used to assess the ability of cells to grow on the materials used (metabolic tests or qualitative imaging to assess the ability of cells to differentiate). However, the results of studies on biomaterials indicate a weak correlation between in vitro studies of biomaterials and the results of in vivo studies on tissue regeneration [63]. It has been found that in vitro assessments of biomaterials are not adequate to predict the results of in vivo tests and it is necessary to improve and/or develop more effective in vitro protocols/tests [64].

Considering the importance of the immune response to the presence of “foreign” material it is surprising that current evaluation protocols do not include early-stage immunoassay. The immune system’s response to the presence of “foreign” material involves the release of activating cytokines and the initiation of a chain of events that leads to inflammatory response [65].

**Figure 8:**
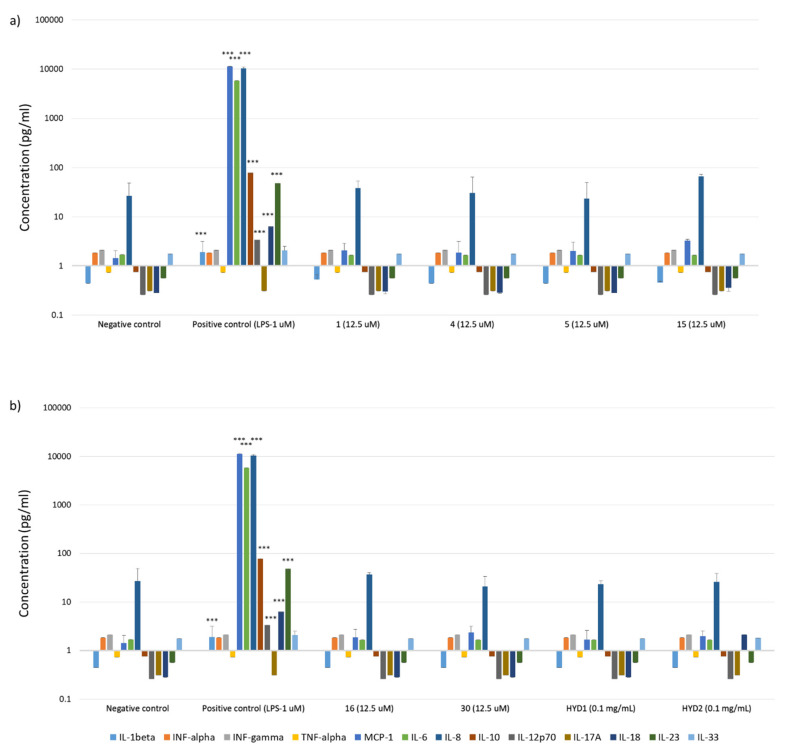
Analysis of the inflammation response (**a**) of peptides **1**, **4**, **5**, **15** and (**b**) **16**, **30**, **HYD1**, **HYD2** on SC cell lines. Biolegend Legendplex human inflammation assay was used to assess the level of the cytokines and chemokines induced by test compounds relative to the negative control. Cells treated with lipopolysaccharide at 1uM concentration constituted the positive control. All of the experiments were performed in triplicate. Data are expressed as mean ± SD (*n* = 3), *** *p* < 0.001 versus the negative control.

Additionally, in the case of collagen IV, its participation in the immunoreactivity of basement membrane in the inflammatory process was demonstrated [66,67]. In addition, it has been found that an elevated level of collagen IV, as well as the presence of collagen IV fragments, may be either a biomarker of the disease state [68,69,70] or show variable biological activity, such as cell growth, differentiation and migration, regulators of angiogenesis, inhibitors of metalloproteinases, dipeptidylpeptidase-IV [71,72,73,74]. Therefore, additionally for selected peptides ^21^AGEKSYGKPC^30^ (1), ^161^LAPGSFKGMK^170^ (4), ^221^GFQGEKGVKG230 (5), ^721^LPGFPGLPGK^730^ (15), ^781^PGLKGVHGKP^790^ (16) and ^1331^DPGFPGMKGK^1340^ (30), studies were carried out on the level of inflammatory responses, mediated by various cytokines and chemokines induced by using LEGENDplex™ Human Inflammation Panel 1 assay (Figure 8). In addition, the products of thermal degradation and subsequent enzymatic hydrolysis with trypsin were used as internal controls. HYD1 is a mixture of peptides obtained from native human collagen IV, while HYD2 is a mixture of peptides obtained from recombinant human collagen IV alpha 6 proteins. The use of native proteins in the test was impossible due to their insolubility in water [75] and the assay medium. The Human Inflammation Panel allows simultaneous quantification of 13 human inflammatory cytokines/chemokines, including IL-1β, IFN-α2, IFN-γ, TNF-α, MCP-1 (CCL2), IL-6, IL-8 (CXCL8), IL-10, IL-12p70, IL-17A, IL-18, IL-23 and IL-33.

Analysis of the inflammatory response indicated that none of the tested collagen IV fragments significantly affected the increase in cytokine and chemokine levels relative to the negative control. Analysis relative to the positive control (LPS) showed statistically significant differences in all collagen IV fragments tested (*p* < 0.001). The obtained results clearly prove that the examined collagen IV fragments do not induce an inflammatory response in SC cells.

### 2.3. Research on the Ability to Self-Organization of Collagen IV Fragments

**Figure 9:**
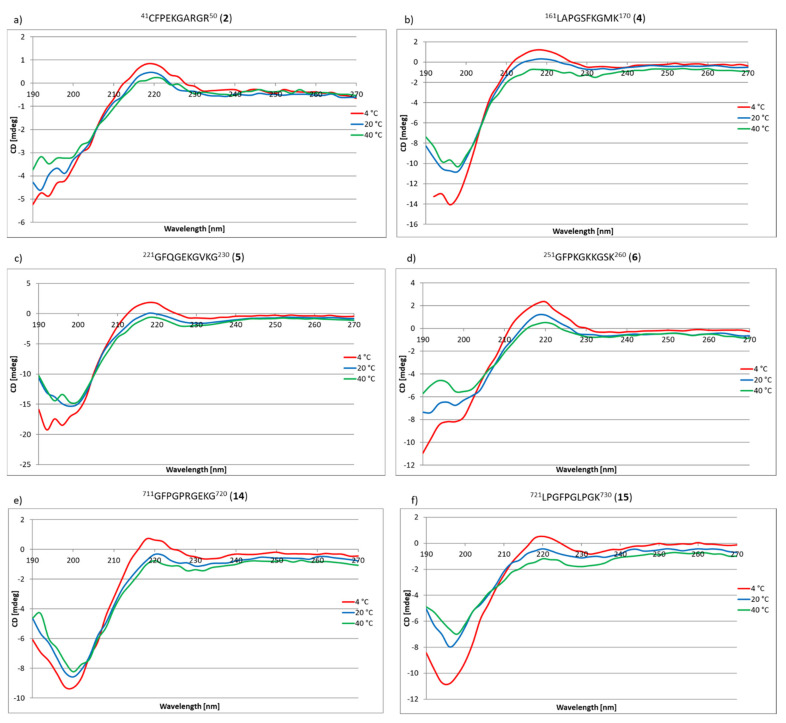

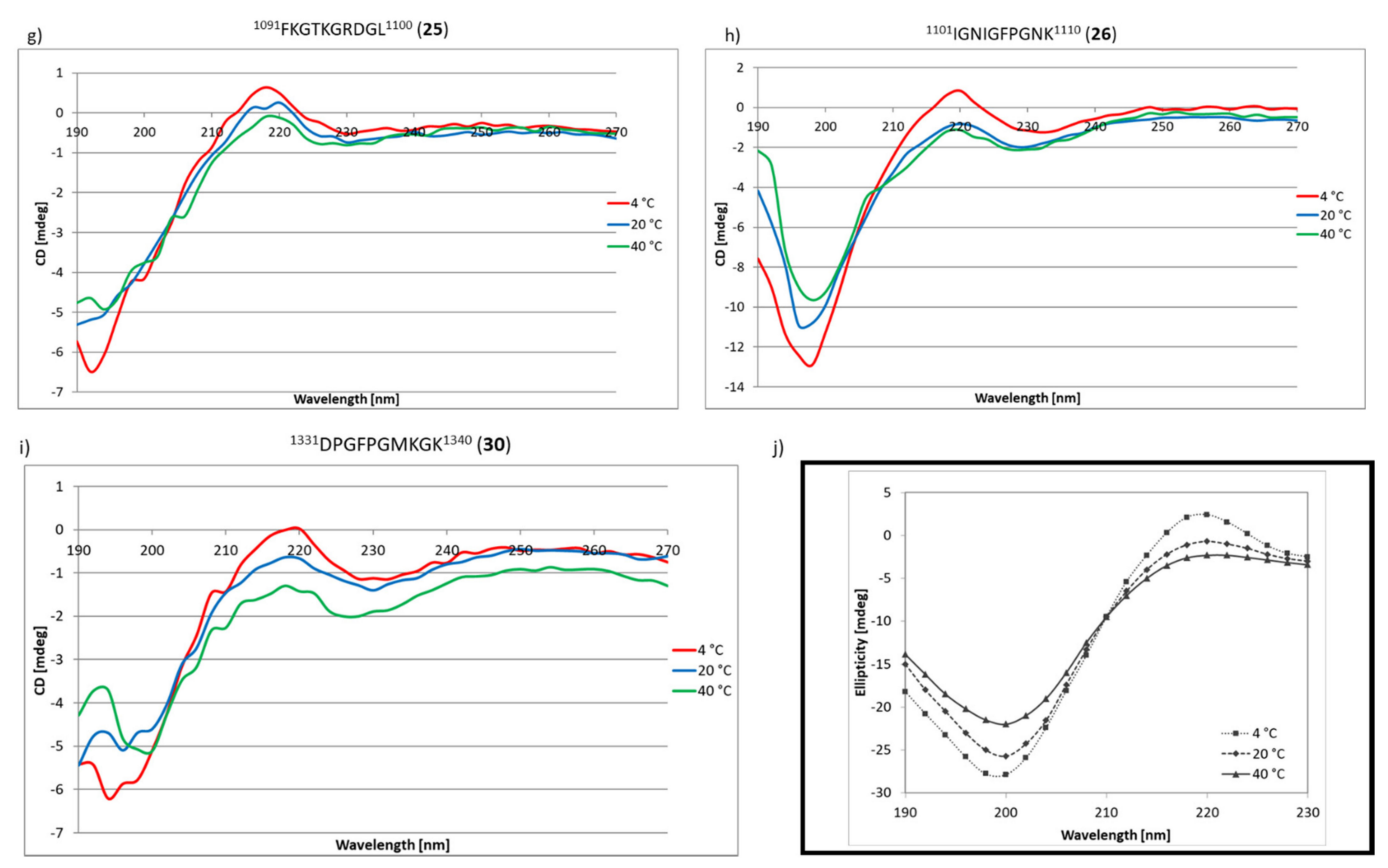
CD spectra of peptides: **2** (**a**), **4** (**b**), **5** (**c**), **6** (**d**), **14** (**e**), **15** (**f**), **25** (**g**), **26** (**h**), and **30** (**i**). (**j**) shows the CD simulated spectrum of the triple helix based on the literature data at different temperatures. The CD spectra were measured at 4 °C, 20 °C and 40 °C, after 24 h of incubation of the aqueous sample solutions at the same temperatures.

The next stage of the research was to check whether peptides **1**–**33** and **2.1**–**2.4** have the ability to self-organize, enabling the formation of structures mimicking the structure of the triple helix characteristic of collagens, including the COL domain of type IV collagen. The method of circular dichroism was used in the research, allowing both the secondary and tertiary structure of peptides and proteins to be determined. This method can also be successfully used to observe the process of protein self-organization depending on the temperature or pH. Additionally, this method is sensitive, does not degrade the sample and is characterized by a satisfactory measurement speed [76]. A characteristic feature of triple helix collagens is the maximum at about 220 nm and the minimum at about 190–200 nm [77]. Based on literature data [78], the CD spectrum corresponding to the triple helix characteristic of collagens as a function of temperature was simulated (Figure 9j). The main factors stabilizing the collagen triple helix are the hydrogen bonds between the hydrogen atom of the amino group and the oxygen atom of the carbonyl group. These are relatively weak bonds [79,80], and their stability depends, inter alia, on temperature. Therefore, the stability of the collagen triple helix changes with the change of this factor. Due to the fact that the studied collagen fragments can be potentially used in regenerative medicine, it was necessary to check the stability of the structure in the temperature range as close as possible to physiological conditions. Therefore, the tests were performed at 4 °C, 20 °C and 40 °C after incubation for 24 h. The incubation process was to enable the self-assembly to form structures that mimic the triple helix of collagen. Out of peptides **1**–**33**, nine fragments (peptides **2**, **4**, **5**, **6**, **14**, **15**, **25**, **26** and **30**) (Figure 9a–i) have the ability to self-organize to form structures that mimic the structure of the triple helix. In the spectra, the presence of a characteristic maximum at a wavelength of about 220 nm and a minimum at about 190–200 nm was observed. However, the most important fact is that these peptides formed stable three-dimensional structures even at 40 °C.

**Figure 10:**
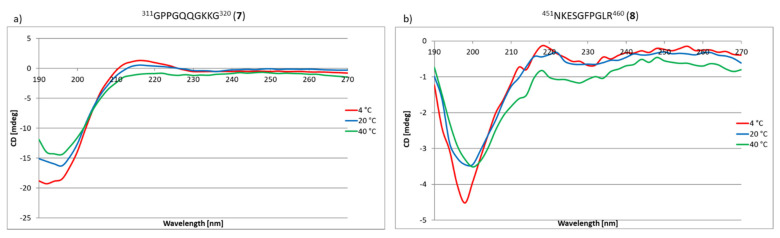

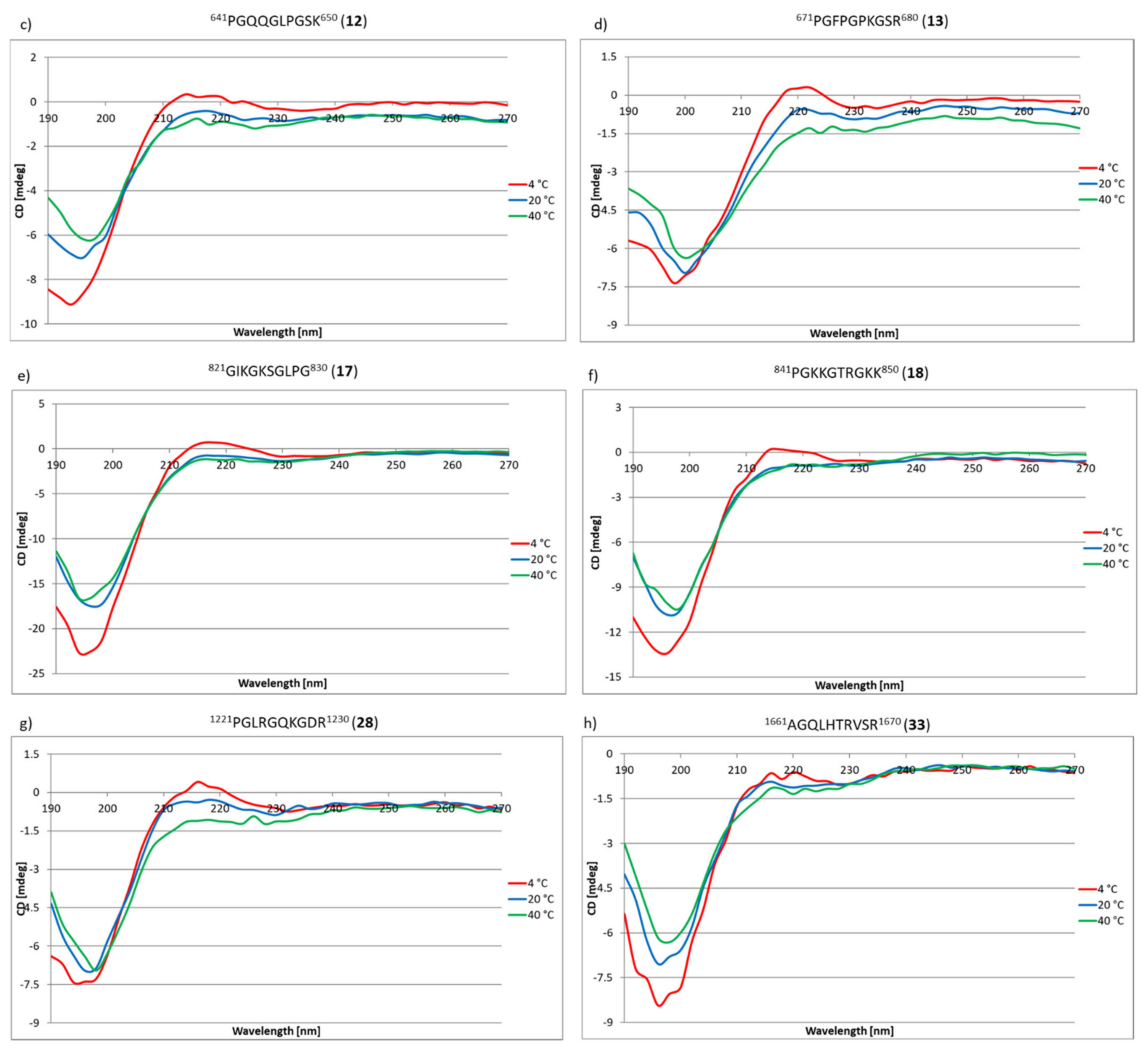

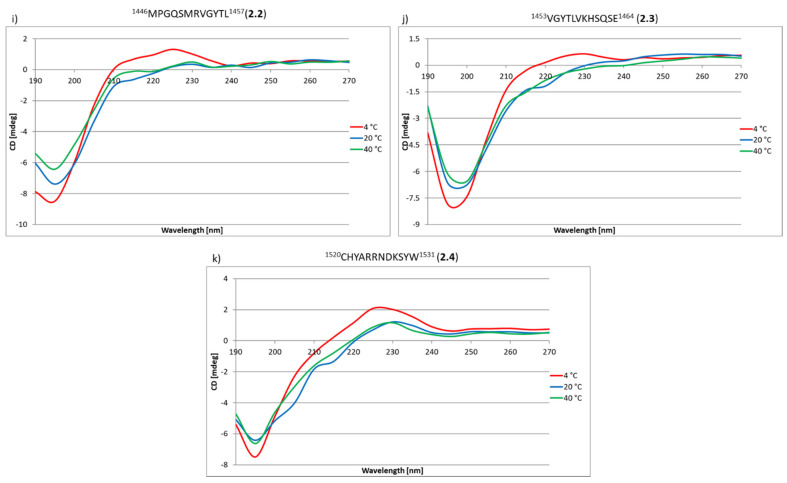
CD spectra of peptides: **7** (**a**), **8** (**b**), **12** (**c**), **13** (**d**), **17** (**e**), **18** (**f**), **28** (**g**), **33** (**h**), **2.2** (**i**), **2.3** (**j**) and **2.4** (**k**). The CD spectra were measured at 4 °C, 20 °C and 40 °C, after 24 h of incubation of the aqueous sample solutions at the same temperatures.

For peptides **7**, **8**, **12**, **13**, **17**, **18**, **28**, **33** and **2.2**, **2.3** and **2.4,** it was found that they are capable of self-assembling and forming the triple helix mimicking structures only at 4 °C (Figure 10).

For the remaining peptides, their susceptibility to self-organization and creation of stable three-dimensional structures were not found (see Figures S75–S92: CD spectra of **1**, **3**, **9**, **10**, **11**, **16**, **19**, **20**, **21**, **22**, **23**, **24**, **27**, **29**, **31**, **32**, **33, 2.1** peptides in the Supplementary Materials).

**Figure 11:**
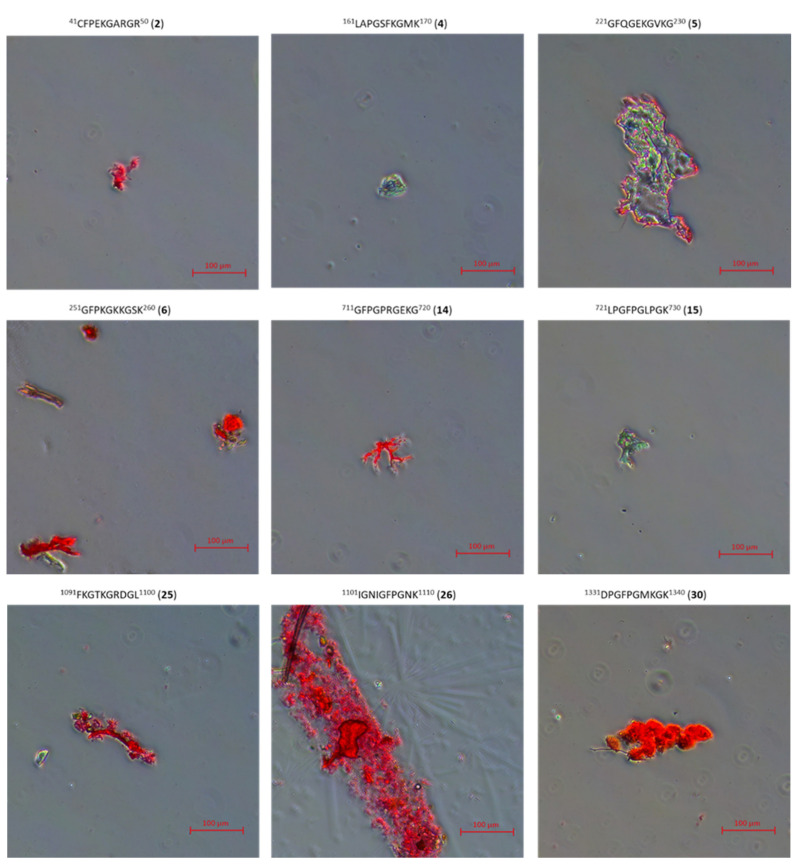
Microscopic picture of peptide **2**, **4**, **5**, **6**, **14**, **15**, **25**, **26**, **30** aggregates stained with Congo Red.

In addition to CD studies, microscopic studies of peptides **1**–**33** and **2.1**–**2.4** stained with Congo Red were also performed. This method of visualization of peptide aggregates is a typical method of studying β-sheet structures in amyloid-type fibers [81,82,83]. For peptides **2**, **4**, **5**, **6**, **14**, **15**, **25**, **26**, **30** (peptides able to aggregate to stable structures mimicking the structure of the collagen helix at 4 °C, 20 °C and 40 °C), microscopic pictures show there are only amorphous structures (Figure 11).

**Figure 12:**
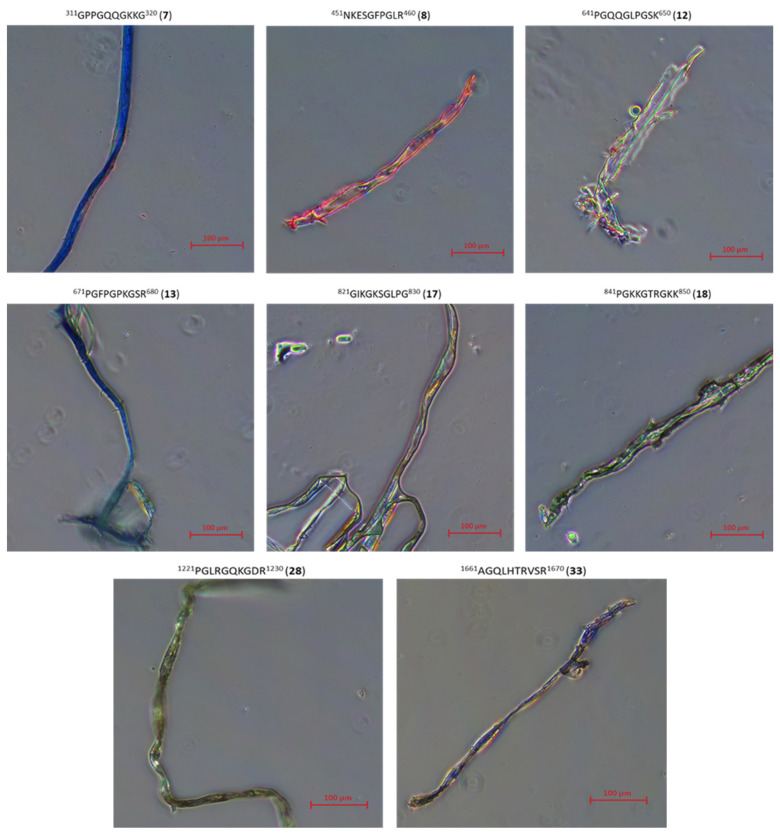

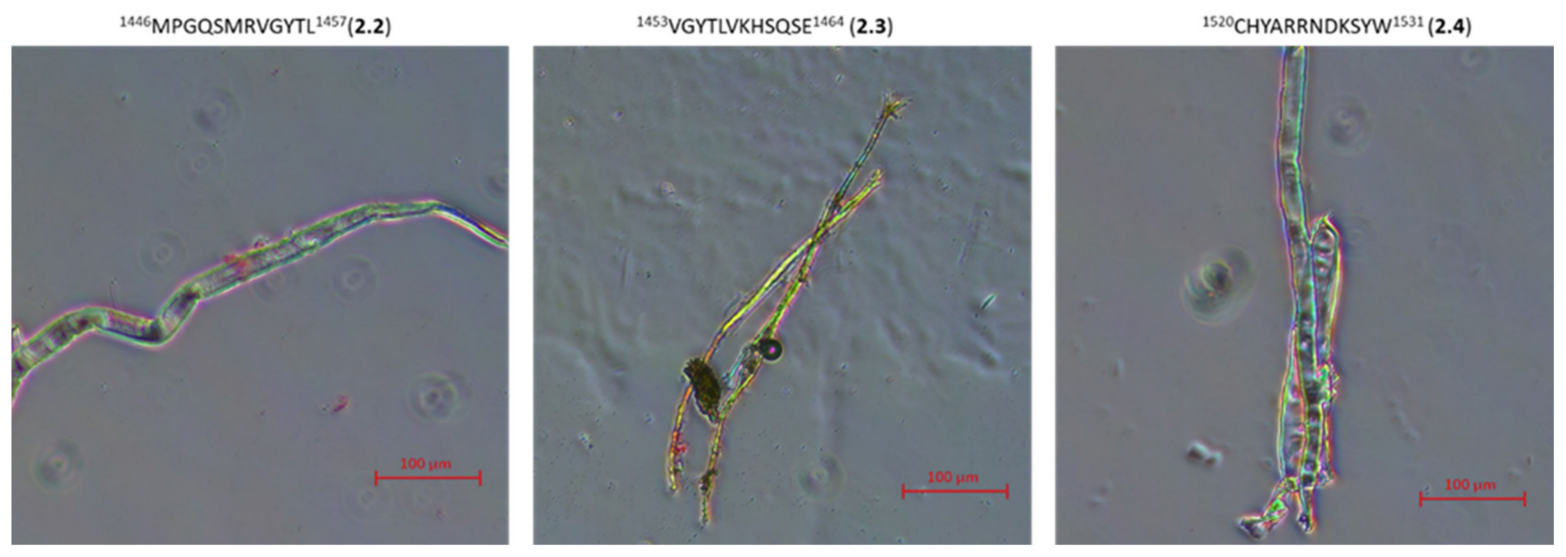
Microscopic picture of peptide **7, 8, 12, 13, 17, 18, 28, 33, 2.2**, **2.3**, **2.4** aggregates stained with Congo Red.

On the other hand, for peptides **7**, **8**, **12**, **13**, **17**, **18**, **28**, **33**, **2.2**, **2.3**, **2.4**, for which it has been shown in CD studies that they are able to form, as a result of aggregation, structures mimicking the collagen helix only at the temperature of 4 °C, it was found that they form fibrous structures (Figure 12), but with a color significantly different from the characteristic red color of amyloid fibrous structures with characteristic β-sheet structural elements.

**Figure 13:**
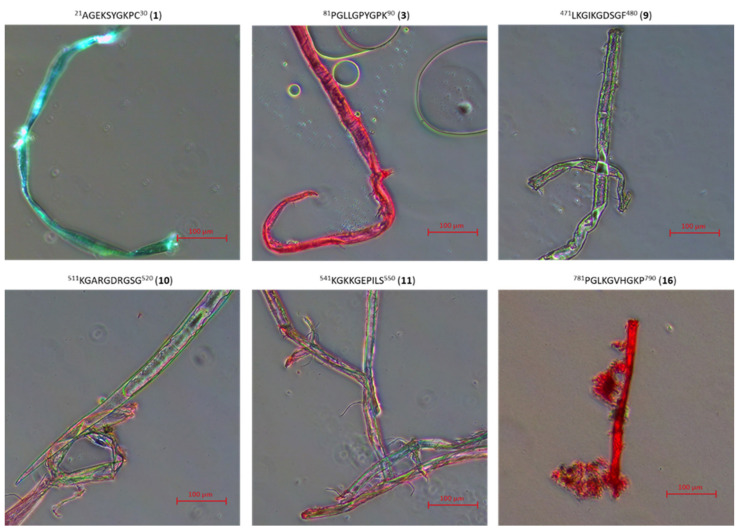

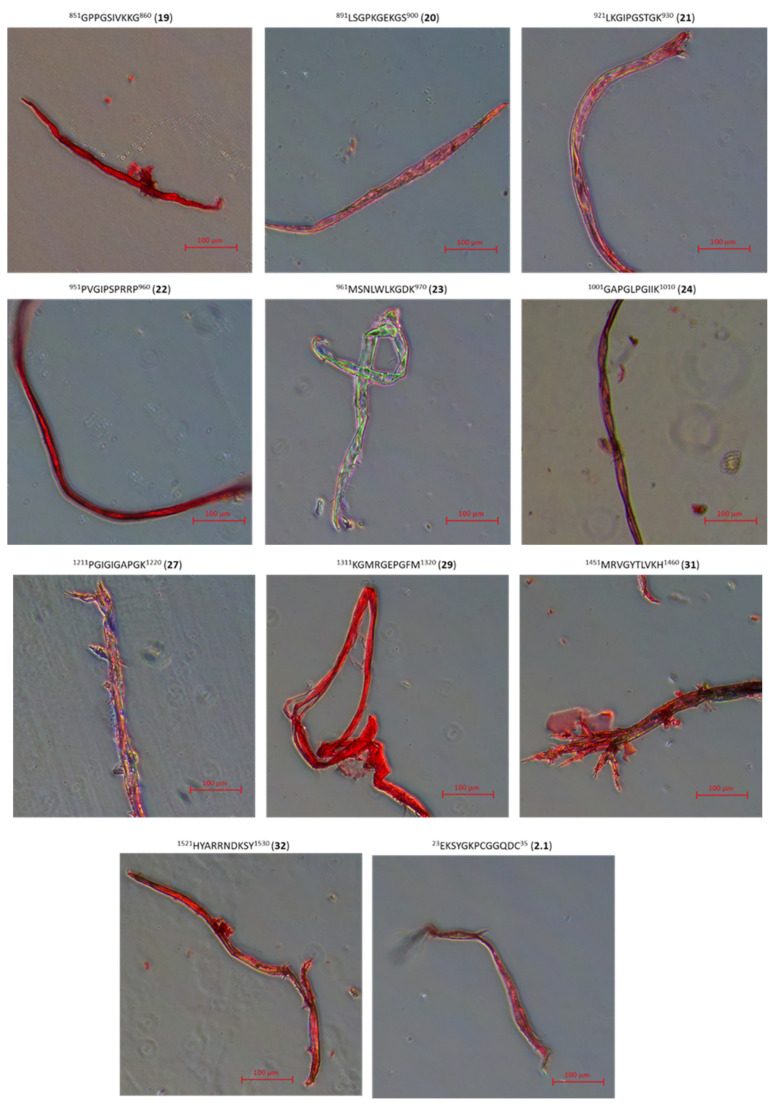
Microscopic picture of peptide 1, 3, 9, 10, 11, 16, 19, 20, 21, 22, 23, 24, 27, 29, 31, 32, 2.1, aggregates stained with Congo Red.

Results of microscopic examination of peptides **1**, **3**, **9**, **10**, **11**, **16**, **19**, **20**, **21**, **22**, **23**, **24**, **27**, **29**, **31**, **32** and **2.1**, classified on the basis of CD outcomes as having no ability to form, due to aggregation, structures mimicking the helical structure of collagen, allowed to state that all of them, after incubation at 37 °C, create fibrous structures resembling amyloid structures characterized by the presence of β-sheet conformers (Figure 13). The obtained fibrous structures were either red (observation in transmitted light) or apple green (polarized light).

**Figure 14:**
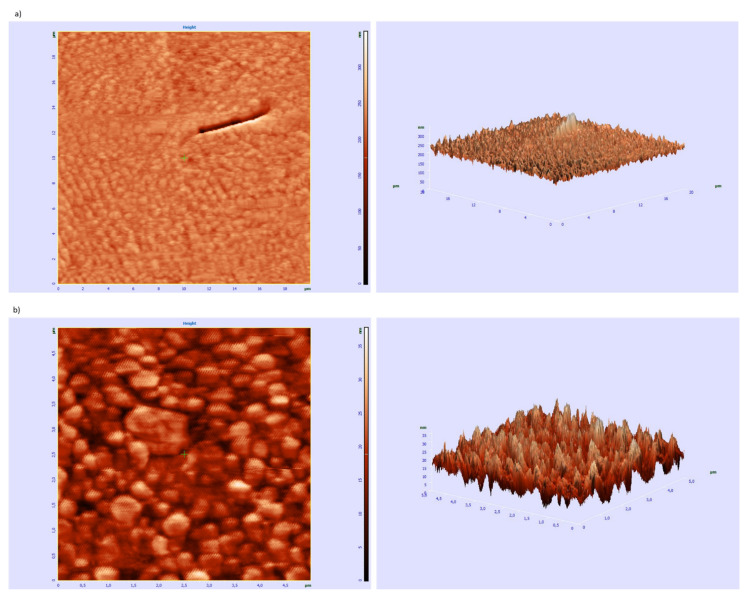
AFM pictures of peptide **8**, (**a**) image on a scale of up to 20 μm, (**b**) zoom image to scale 5 μm.

For peptide **8** ^451^NKESGFPGLR^460^, which as a result of self-organization only at a lowered temperature forms a spatial structure that mimics the helical structure of collagen, the AFM studies were performed. The obtained images show two types of self-organization. One of them can be described as a lamellar organization (Figure 14a).

The second way leads to aggregates in a form of spherulites or irregular bigger structures (Figure 14b). That can suggest aggregation in water solution, prior to subsidence on the silicon surface.

### 2.4. Interaction Study of Collagen IV Fragments with α1β1 Integrin (ITGα1β1)

In addition to ensuring the mechanical stability of the basement membrane, collagen IV is involved in interaction with various cell types. These interactions are critical to a variety of biological processes including cell adhesion, migration, survival, proliferation and differentiation. Both collagen IV fragments located within the triple helix and the NC1 domain are involved in the interaction with cell receptors, which suggests the participation of various types of adhesion receptors [84]. The main receptors with which collagen IV interacts are integrins and non-integrin receptors. On the other hand, peptides derived from collagen IV can disrupt the interaction with integrins, in particular with α5β1 integrin, and in parallel activate Ang2/Tie2 signa-ling pathway essential for maintaining vascular homeostasis [85].

**Figure 15:**
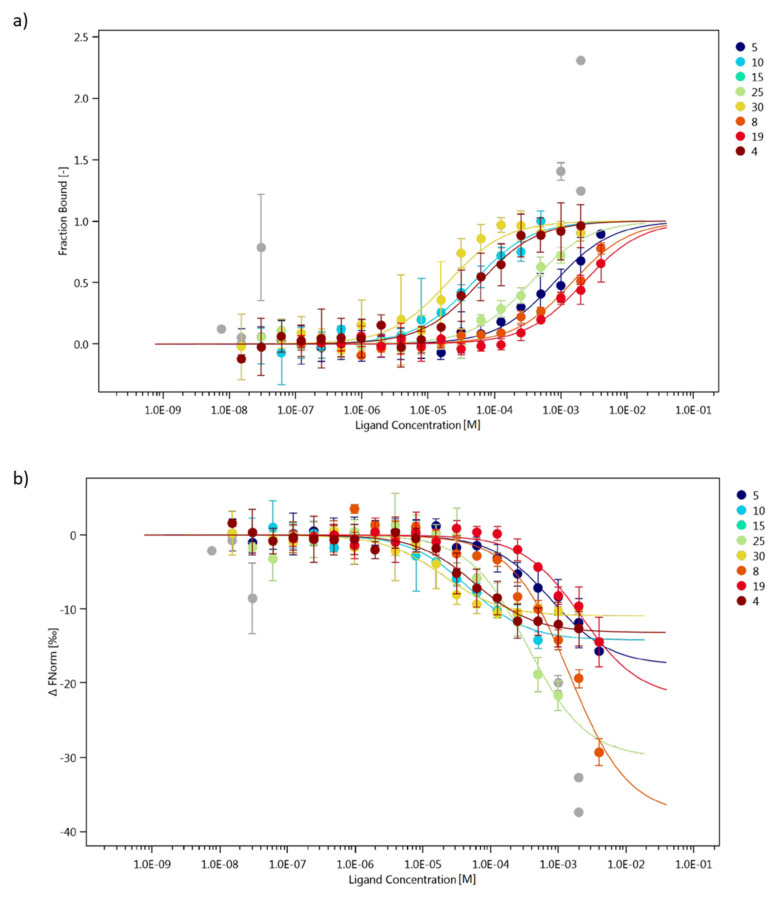
MST determination of K_d_ for interactions of the labeled α1β1 integrin and the selected fragments of collagen IV (peptides **4**, **5**, **8**, **10**, **15**, **19**, **25** and **30**). The α1β1 integrin concentration was kept constant (25 nM), while the non-labeled binding partner collagen IV peptides were used at increasing concentrations as the titration ligand. The MST measurement was performed at 80% or 100% (for peptide **4**) LED power and medium (40%) MST power. An MST-on time 4 s was used for analysis and a K_d_ (µM ± µM confidence) was derived for these interactions (*n* ≥ 3 independent measurements, error bars represent standard deviation). (**a**) shows the fraction bound plotted against the ligand concentration [M], (**b**) shows the dependence of ΔFnorm (Baseline Corrected Normalized Fluorescence) against the concentration of the ligand [M].

Attempts were made to check the ability of selected fragments of collagen IV to interact with α1β1 integrin (ITGα1β1). The tests were performed using the Microscale Thermophoresis (MST) technique [86]. The following fragments were used for the research: **4**, **5**, **8**, **10**, **15**, **19**, **25** and **30**. The selected fragments included peptides that were able to mimic the spatial structure of collagen at 4 °C, 20 °C and 40 °C (peptides **4**, **5**, **15**, **25** and **30**), a fragment mimicking the spatial structure of collagen only at 4 °C (peptide **8**) and two fragments that do not have the ability to aggregate into stable three-dimensional structures (peptides **10** and **19**). For MST experiment His-tagged ITGα1β1 was labeled using a fluorescent dye, which enable a site-specific and stable noncovalent fluorescence labeling of polyhistidine-tagged proteins. The concentrations of labeled ITGα1β1 were kept constant at 25 nM, whereas the peptides were used as titration ligands with increasing concentrations. Figure 15a shows the concentration-related fraction bound, while Figure 15b shows the concentration-dependence of ΔFnorm (Baseline Corrected Normalized Fluorescence). Peptide **30** was found to have the highest affinity for ITGα1β1. This fragment is characterized by the ability to self-organize and form spatial structures mimicking the structure of collagen at all tested temperatures. The ability to bind collagen IV fragments to the α1β1 integrin decreased as follows: peptide **10** > peptide **4** > peptide **25** > peptide **5** > peptide **8** > peptide **19**. There was no binding to integrin for peptide **15.** Analyzing the relationship between the ability to bind collagen IV fragments and the ability to form spatial structures mimicking the structure of collagen, it can be concluded that three fragments (peptides **4**, **5** and **30**) establish a stable spatial structure even at 40 °C form complexes with integrin α1β1. Peptide **8**, capable of forming the stable spatial structure only at a reduced temperature, was also associated with ITGα1β1. Binding to ITGα1β1 was also observed for fragments **10** and **19**, which did not have the ability to form helical-type structures. These observations indicate that in the case of interactions with α1β1 integrin, the spatial structure of the ligands is not the only factor determining the interaction capacity.

The literature data [87] show that the GFPGER sequence of collagen is the most strongly bound ligand to ITGα1β1. This structural motif is marked in the table below. Table 3 presents a summary of the analyzed peptides, taking into account the results of the CD tests (ability to form structures mimicking the collagen helix), the determined dissociation constant and the maximum concentration in the MST test. The highest concentrations were fitted to capture the inflection points of the curves. Figure 15b shows that for peptides with higher K_d_ values, that is, peptides **8**, **19**, no flattening is visible after the inflection point, while for peptides **5** and **25** only its outline is visible.

By analyzing the dependence of the ability to bind to ITGα1β1, it can be seen that the presence of basic amino acids may have an effect on protein binding. Peptide **15**, which does not bind to integrin, has only one lysine, while the remaining peptides have two or three lysine or arginine residues each. A KG sequence is present in all binding peptides (except **8**).

Taking into account the recognizable sequence “GFPGER”, it cannot be clearly stated that its presence determines the binding to ITGα1β1. The same part of this sequence (GFPG) is present at the same position in the highest affinity peptide **30** and the non-protein-interacting peptide **15**. The difference is the presence of a lysine residue in the peptide **30** at the site where, according to the recognizable sequence, arginine should be located, i.e., also an amino acid of basic nature, while the proline is present in peptide **15** at this position.

Overall, these two sequences only differ in three amino acids. In peptide **30** they are D, M and K, while in peptide **15** they are two leucine residues and a proline residue. The non-interacting peptide **15** has more nonpolar (hydrophobic) amino acid residues compared to peptide **30**. Peptide **8** has almost all of the recognizable sequences, but its affinity is low, and the same applies to peptide **5**. Peptide **4**, on the other hand, binds quite strongly to TGα1β1 without any literature ligand element (it contains glycine and lysine, but their arrangement does not match the recognizable motif).

### 2.5. Study of the Usefulness of Selected Collagen IV Fragments in Obtaining Porous Materials

**Figure 16:**
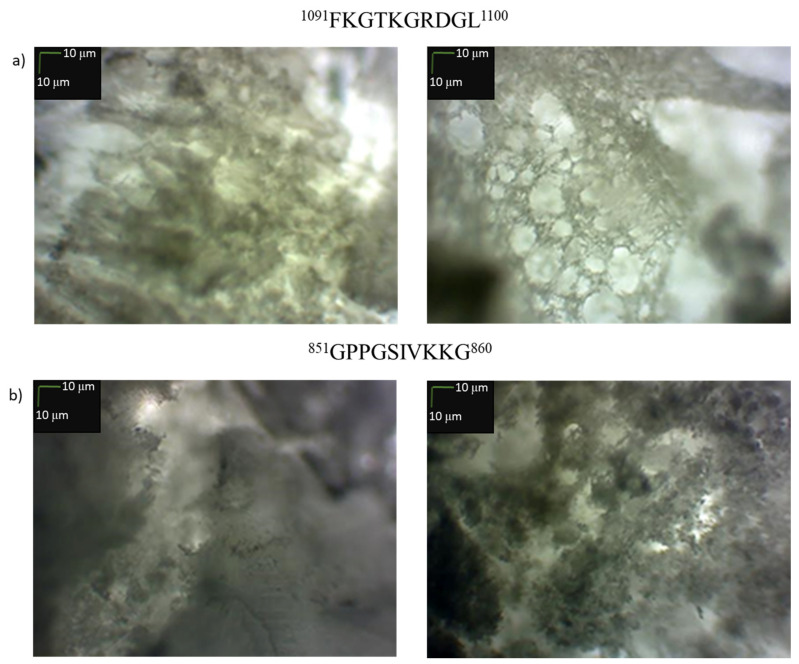
Porous structures formed by (**a**) ^1091^FKGTKGRDGL^1100^ (**25**) a peptide having the ability to mimic the structure of the triple helix, (**b**) ^851^GPPGSIVKKG^860^ (**19**) fragment that does not have the ability to mimic the structure of the triple helix at any of the analyzed temperatures.

Attempts were also made to obtain porous structures formed solely as a result of self-organization (no cross-linking was used) from two selected fragments. One was used that aggregates into a conformer mimicking the triple helix structure at each of the analyzed temperatures (^1091^FKGTKGRDGL^1100^, ^25^) and a collagen IV fragment that did not show this ability (^851^GPPGSIVKKG^860^, **19**). For this purpose, 100 mg of each peptide was dissolved in 2 mL of water and incubated for 24 h at 40 °C. Then, solid CO_2_ (100 mg) used as a blowing agent was added to the solution and the solution was thoroughly stirred, frozen in liquid nitrogen and freeze-dried. Pictures of the obtained structures are shown in Figure 16.

The microscopic picture (Figure 16) confirms the formation of a porous structure by both peptides. The more porous structure was formed by the fragment ^1091^FKGTKGRDGL^1100^ self-assembling into a stable spatial structure mimicking the collagen triple helix. However, in order to obtain a porous stable structure, it is necessary to cross-link them. It can also be expected that this process should prevent rapid degradation of collagen-based biomaterials in vivo [88].

**Figure 17:**
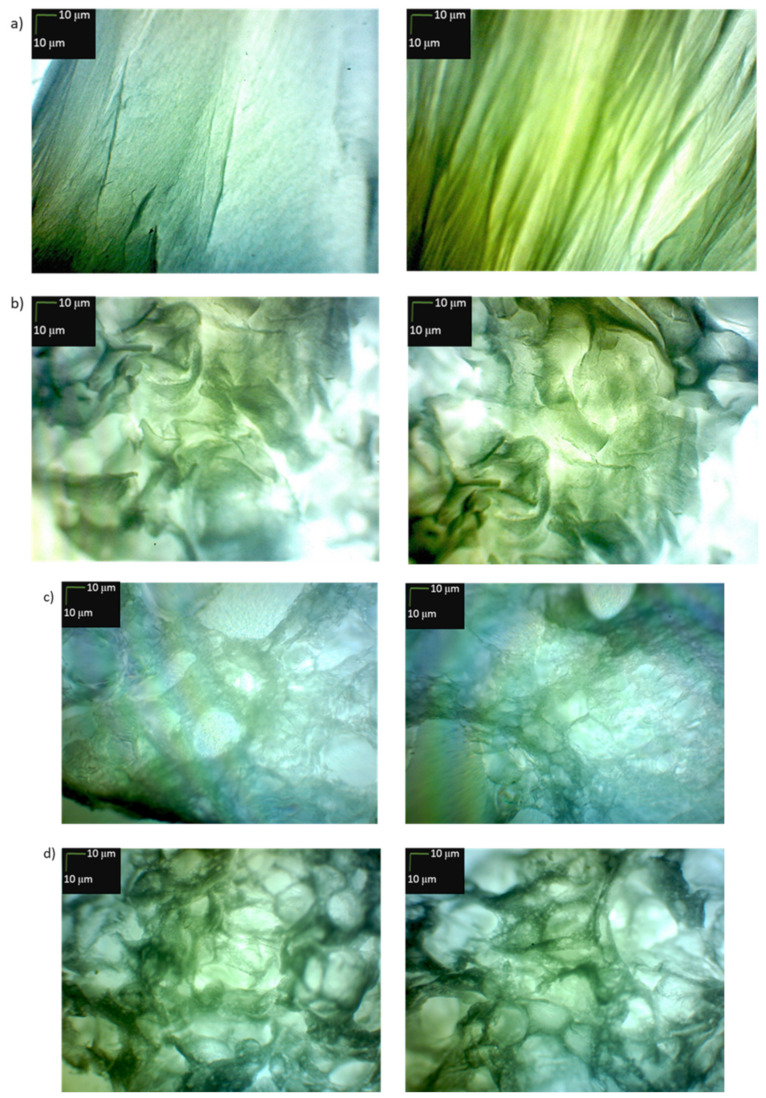

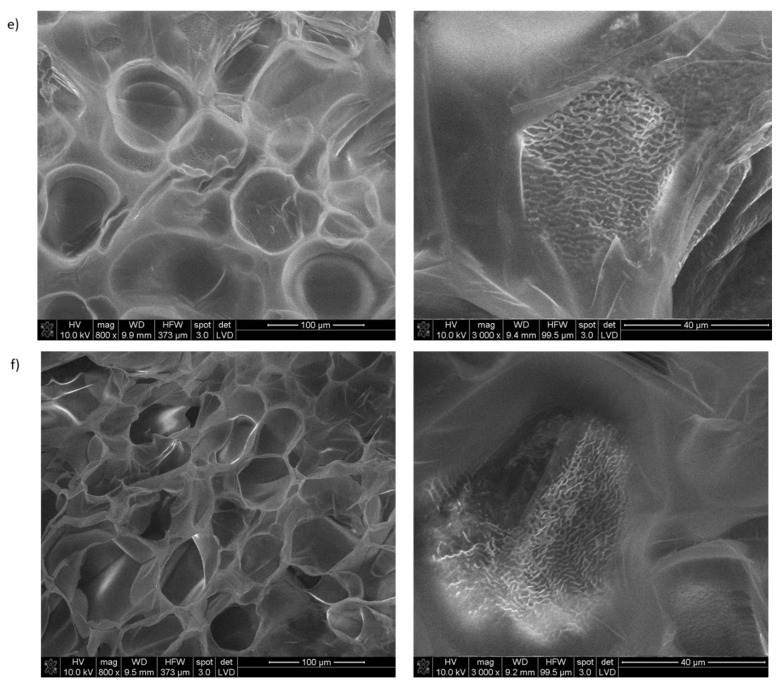
Solid structures formed by the equimolar mixture of peptides **2**, **4**, **5**, **6**, **14**, **15**, **25, 26** and **30**. (**a**) material obtained after lyophilization of the solution and incubation for 24 h at 40 °C; (**b**) material obtained after lyophilization of the solution and incubation for 24 h at 40 °C and adding solid CO_2_; (**c**) the material obtained after cross-linking with DMT/NNN/TosO^−^ of the solid material obtained under conditions (**a**); (**d**) the material obtained after cross-linking with DMT/NNN/TosO^−^ of the solid material obtained under the conditions (**b**); (**e**) SEM picture of the material obtained after cross-linking with DMT/NNN/TosO^−^ of the solid material obtained under conditions (**a**); (**f**) SEM picture of the material obtained after cross-linking with DMT/NNN/TosO^−^ of the solid material obtained under the conditions (**b**).

Encouraged by these results, we attempted to obtain porous materials using an equimolar mixture of peptides **2**, **4**, **5**, **6**, **14**, **15**, **25**, **26** and **30**, that is, those that formed stable spatial structures at all tested temperatures. For the preparation of the peptide mixture, 0.1 μmole of each peptide was used. The experiment without cross-linking was also repeated (Figure 17a,b). In one case, a solution of peptides **2**, **4**, **5**, **6**, **14**, **15**, **25**, **26** and **30** in water, after incubation at 40 °C, was frozen and lyophilized (Figure 17a). In the second case, solid CO_2_ was added to the solution after incubation as a blowing agent, and the suspension was also lyophilized (Figure 17b).

In the case of the material obtained without solid CO_2_, the obtained image shows the formation of fibrous and/or amorphous structures. On the other hand, the material obtained with the use of CO_2_ (Figure 17b) was porous. Solid materials consisting of peptides **2**, **4**, **5**, **6**, **14**, **15**, **25**, **26** and **30** obtained by lyophilization were used for their cross-linking. The method of collagen cross-linking using EDC and NHS was adopted [89], DMT/NMM/TosO^−^ was applied as the coupling reagent because it can also be successfully used in aqueous solutions [90,91]. Since the cross-linking takes place in the solid material, the first step consists in diffusing the condensing reagent solution into the solid material, and only then the coupling between the carboxyl and amine groups is possible. The cross-linking time was 60 min. After cross-linking was complete, the excess coupling reagent was removed by washing with 0.1 M Na_2_HPO_4_ solution and deionized water.

Cross-linking of the materials significantly improved the porosity of the formed structures. The material obtained as a result of cross-linking of the derivative (b) (Figure 17d) is characterized by high porosity. Also, the porous structure was obtained by cross-linking (Figure 17c) of the material (a) which appeared to have a fibrous and/or amorphous structure. Based on SEM studies, it was found that the cross-linked material obtained with the addition of solid CO_2_ (material obtained under the conditions (b)) has more regular and smaller pores in the structure (Figure 17f). The presence of open-pore structures is observed in both products obtained as a result of freeze-drying. However, an advantageously higher number of porous structures can be observed in the case of the procedure using additional solid CO_2_.

**Figure 18:**
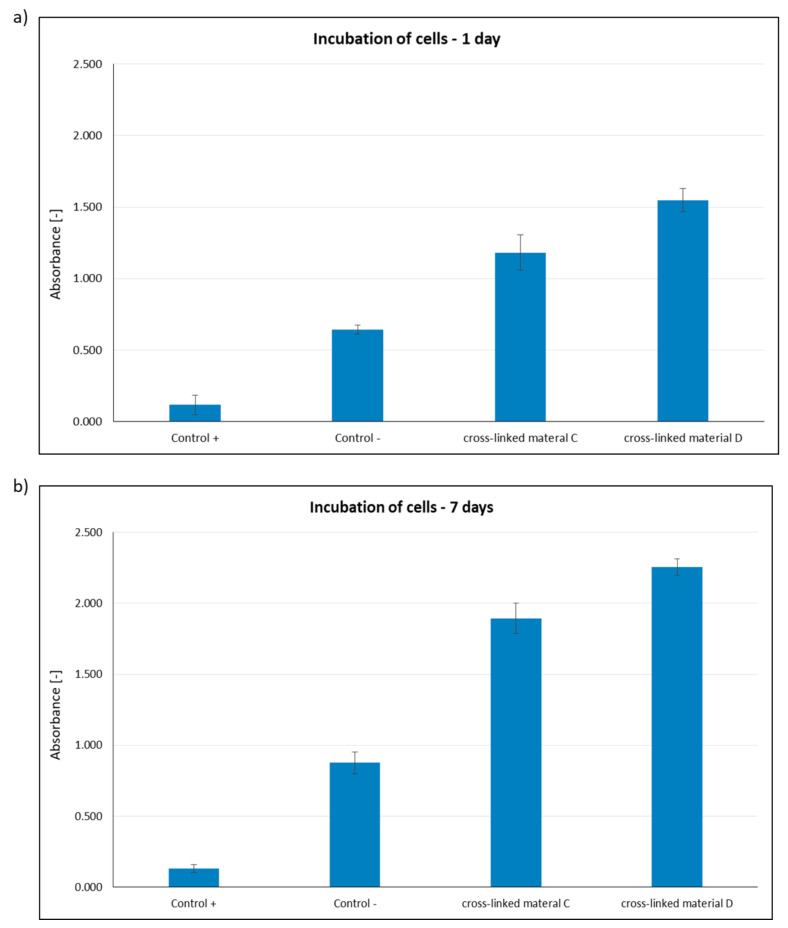
Cell metabolic activity of the BJ cell line cultured in the presence of material C and D. (**a**): cells incubated in the presence of materials for 1 day; (**b**): cells incubated in the presence of materials for 7 days. Cytotoxicity tests were performed in five replicates, using 3-(4,5-dimethylthiazol-2-yl)-2,5-diphenyltetrazolium bromide (MTT test). No statistical significance was observed between the samples incubated for 1 and 7 days.

Porous materials C (obtained by cross-linking with DMT/NMM/TosO^−^ solid material obtained by lyophilization of equimolar mixture of peptides **2**, **4**, **5**, **6**, **14**, **15**, **25**, **26** and **30** pre-incubated for 24 h at 40 °C) and D (obtained by cross-linking with DMT/NMM/TosO^−^ solid material obtained in freeze-drying of previously incubated for 24 h at 40 °C equimolar mixture of peptides **2**, **4**, **5**, **6**, **14**, **15**, **25**, **26** and **30** with the addition of solid CO_2_) were used to check their effect on the BJ cell line. Experiments were performed after 1 day (Figure 18a) and 7 days (Figure 18b) of incubation to assess the effects of the cross-linked solid materials from selected collagen IV fragments over the short and long term. This is important from the point of using the materials in regenerative medicine. An MTT test was used to determine the cytotoxicity (correlation to the metabolic activity of cells) of the cross-linked material based on collagen IV. As the Control^−^, cells cultured in the medium without the addition of any substances or any harmful factors were used. As the Control^+^ we used cells cultured with the addition of DMSO on the second day of incubation. DMSO was added to a 5% final concentration in the culture medium.

Based on the results of cytotoxicity studies, it can be concluded that cross-linked material D is not cytotoxic. In the case of cross-linked material C, the absorbance value was lower, but still significantly higher than the Control^−^. The significant increase in absorbance after 7 days of incubation directly indicates that the number of cells in the culture medium must have increased radically, which also confirms the proliferation of cells grown in the presence of an extract of cross-linked material.

Porous materials derived from collagen IV peptides have a high potential for applicability in regenerative medicine. Satisfactory results of biological studies of collagen IV fragments reconstructing the external sphere of the native protein, capable of forming, through self-organization, stable spatial structures imitating the structure of the native protein confirm this hypothesis. Research into the full characterization of porous structures and their use as scaffolds for tissue regeneration will continue.

## 3. Materials and Methods

### 3.1. General Information

Collagen IV fragments ^21^AGEKSYGKPC^30^ (**1**), ^41^CFPEKGARGR^50^ (**2**), ^81^PGLLGPYGPK**90** (**3**), ^161^LAPGSFKGMK^170^ (**4**), ^221^GFQGEKGVKG^230^ (**5**), ^251^GFPKGKKGSK^260^ (**6**), ^311^GPPGQQGKKG^320^ (**7**), ^451^NKESGFPGLR^460^ (**8**), ^471^LKGIKGDSGF^480^ (**9**), ^511^KGARGDRGSG^520^ (**10**), ^541^KGKKGEPILS^550^ (**11**), ^641^PGQQGLPGSK^650^ (**12**), ^671^PGFPGPKGSR^680^ (**13**), ^711^GFPGPRGEKG^720^ (**14**), ^721^LPGFPGLPGK^730^ (**15**), ^781^PGLKGVHGKP^790^ (**16**), ^821^GIKGKSGLPG^830^ (**17**), ^841^PGKKGTRGKK^850^ (**18**), ^851^GPPGSIVKKG^860^ (**19**), ^891^LSGPKGEKGS^900^ (**20**), ^921^LKGIPGSTGK^930^ (**21**), ^951^PVGIPSPRRP^960^ (**22**), ^961^MSNLWLKGDK^970^ (**23**), ^1001^GAPGLPGIIK^1010^ (**24**), ^1091^FKGTKGRDGL^1100^ (**25**), ^1101^IGNIGFPGNK^1110^ (**26**), ^1211^PGIGIGAPGK^1220^ (**27**), ^1221^PGLRGQKGDR^1230^ (**28**), ^1311^KGMRGEPGFM^1320^ (**29**), ^1331^DPGFPGMKGK^1340^ (**30**), ^1451^MRVGYTLVKH^1460^ (**31**), ^1521^HYARRNDKSY^1530^ (**32**), ^1661^AGQLHTRVSR^1670^ (**33**), ^23^EKSYGKPCGGQDC^35^ (**2.1**), ^1446^MPGQSMRVGYTL^1457^ (**2.2**), ^1453^VGYTLVKHSQSE^1464^ (**2.3**), ^1520^CHYARRNDKSYW^1531^ (**2.4**) were synthetized on chlorotrityl resin according the Fmoc/tBu strategy.

- Devices used in determining the purity and confirming the structure of the tested collagen fragments IV

Analytical High Performance Liquid Chromatograpy (HPLC) was performed on an LC Dionex UltiMate 3000 (ThermoFisher Scientific, Waltham, MA, USA), using a Kinetex Reversed Phase C18 column (100 × 4.6 mm).

Gradients of 0.1% TFA in H_2_O (B) and 0.1% TFA in CH_3_CN (A) were used, at a flow rate of 0.4 mL/min with UV detection at 220 and 254 nm.

Gradient method 1: A (0.1% TFA in CH_3_CN) and B (0.1% TFA in H_2_O) 30:70 to 90:10 in 30 min, followed by an isocratic run for 5 min.

Gradient method 2: A (0.1% TFA in CH_3_CN) and B (0.1% TFA in H_2_O) 5:95 to 90:10 in 30 min, followed by an isocratic run for 5 min.

Preparative HPLC was carried out on a CombiFlash, EZPrep, Teledyne ISCO (Lincoln, NE, USA) using a Supelco Discovery BIO Wide Pore C18 column (25 cm × 21.2 mm, 10 mm; Sigma-Aldrich) flow rate 5 mL/min, detection wavelengths 220 and 254 nm, gradient ratio A (0.1% TFA in CH_3_CN) and B (0.1% TFA in H_2_O) 0:100 to 82:18 in 30 min, followed by an isocratic run for 5 min.

Mass Spectrometry (MS) analysis was performed on a Bruker microOTOF-QIII (Bruker Corporation, Billerica, MA, USA).

- General Procedures for Synthesis of Peptides **1**–**33** and **2.1**–**2.4**.

GP 1—Attachment of C-terminal amino acid to 2-chlorotrityl chloride resin

To swelled in DCM (10 mL) 2-chlorotrityl chloride resin with loading = 1.0 mmol/g was added a mixture consisting of Fmoc-protected amino acid (3 equivalents related to the resin) and six equivalents of EtNiPr2 were dissolved in CH_2_Cl_2_ (10 mL/g resin). The suspension was shaken for 60 min. The resin was filtered and washed with CH_2_Cl_2_/MeOH/EtNiPr_2_ 17:2:1 (3×), DMF (2×) and CH_2_Cl_2_ (3×). The resin with attached C-terminal amino acid was dried in a vacuum desiccator to constant mass.

GP 2—Coupling

For every coupling, the mixture consisting of 3 equivalents of the amino acid, 3 equivalents of 4-(4,6-dimethoxy-1,3,5-triazin-2-yl)-4- methylmorpholinium toluene-4-sulfonate (DMT/NMM/TosO^−^) [53] and 6 equivalents of N-methylmorpholine (NMM) dissolved in N,N-dimethylformamide (DMF) (final amino acid concentration in DMF 0.5 mM) was used. The solution was added to the resin. The suspension was shaken for 1–2 h. The Kaiser or chloranil tests were used to monitor the completion of the reaction.

GP 3—Deprotection

The Fmoc protecting group was removed using 25% piperidine solution in DMF (2 × 5 min).

GP 4—Cleavage from the Resin

The peptides were cleaved from the resin by using a mixture (ca. 2 mL/0.1 g resin) of 95% TFA (2,2,2-trifluoroacetic acid), 2.5% H_2_O and 2.5% TIS (triisopropylsilane) or a mixture consisting of 94% TFA, 2.5% H_2_O, 2.5% EDT and 2.5% TIS for peptides containing Met or Cys. Cleavage was performed over 4 h at room temperature. The crude product was lyophilized, identified by MS. The purity of the final product was determined by RP-HPLC.

### 3.2. Peptide Synthesis

- Synthesis of ^21^AGEKSYGKPC^30^ (**1**)

Starting materials: resin (1.0 g, 1.0 mmol) and for each coupling: Fmoc-Cys(Trt)-OH (1.757 g, 3.0 mmol), Fmoc-Pro-OH (1.012 g, 3.0 mmol), Fmoc-Lys(Boc)-OH (1.405 g, 3.0 mmol), Fmoc-Gly-OH (0.892 g, 3.0 mmol), Fmoc-Tyr(tBu)-OH (1.379 g, 3.0 mmol), Fmoc-Ser(tBu)-OH (1.150 g, 3.0 mmol), Fmoc-Lys(Boc)-OH (1.405 g, 3.0 mmol), Fmoc-Glu(OtBu)-OH (1.276 g, 3.0 mmol), Fmoc-Gly-OH (0.892 g, 3.0 mmol), Fmoc-Ala-OH (0.934 g, 3.0 mmol). DMT/NMM/TosO^−^ (1.239 g, 3.0 mmol) and NMM (0.66 mL, 6.0 mmol). HPLC (30–90% A in 30 min, Method 1): *t*_R_ = 2.9 min, purity = 88%.MS: 1039.5492, 529.2788 ([M + H]^+^, C_44_H_71_N_12_O_15_S^+^, [M + 2H]^2+^, calc. 1038.47)

- Synthesis of ^41^CFPEKGARGR^50^ (**2**)

Starting materials: resin (1.0 g, 1.0 mmol) and for each coupling: Fmoc-Arg(Pbf)-OH (1.946 g, 3.0 mmol), Fmoc-Gly-OH (0.892 g, 3.0 mmol), Fmoc-Arg(Pbf)-OH (1.946 g, 3.0 mmol), Fmoc-Ala-OH (0.934 g, 3.0 mmol), Fmoc-Gly-OH (0.892 g, 3.0 mmol), Fmoc-Lys(Boc)-OH (1.405 g, 3.0 mmol), Fmoc-Glu(OtBu)-OH (1.276 g, 3.0 mmol), Fmoc-Pro-OH (1.012 g, 3.0 mmol), Fmoc-Phe-OH (1.162 g, 3.0 mmol), Fmoc-Cys(Trt)-OH (1.757 g, 3.0 mmol). DMT/NMM/TosO^−^ (1.239 g, 3.0 mmol) and NMM (0.66 mL, 6.0 mmol). HPLC (30–90% A in 30 min, Method 1): *t*_R_ = 2.2 min, purity = 80%.MS: 1120.6136, 374,2167 ([M + H]^+^, C_47_H_78_N_17_O_13_S^+^, [M + 3H]^3+^, calc. 1119.55).

- Synthesis of ^81^PGLLGPYGPK^90^ (**3**)

Starting materials: resin (1.0 g, 1.0 mmol) and for each coupling: Fmoc-Lys(Boc)-OH (1.405 g, 3.0 mmol), Fmoc-Pro-OH (1.012 g, 3.0 mmol), Fmoc-Gly-OH (0.892 g, 3.0 mmol), Fmoc-Tyr(tBu)-OH (1.379 g, 3.0 mmol), Fmoc-Pro-OH (1.012 g, 3.0 mmol), Fmoc-Gly-OH (0.892 g, 3.0 mmol), Fmoc-Leu-OH (1.060 g, 3.0 mmol), Fmoc-Leu-OH (1.060 g, 3.0 mmol), Fmoc-Gly-OH (0.892 g, 3.0 mmol), Fmoc-Pro-OH (1.012 g, 3.0 mmol). DMT/NMM/TosO^−^ (1.239 g, 3.0 mmol) and NMM (0.66 mL, 6.0 mmol). HPLC (30–90% A in 30 min, Method 1): *t*_R_ = 11.5 min, purity = 86%. MS: 998.6127, 499.8145 ([M + H]^+^, C_48_H_76_N_11_O_12_^+^, [M + 2H]^2+^, calc. 997.55).

- Synthesis of ^161^LAPGSFKGMK^170^ (**4**)

Starting materials: resin (1.0 g, 1.0 mmol) and for each coupling: Fmoc-Lys(Boc)-OH (1.405 g, 3.0 mmol), Fmoc-Met-OH (1.114 g, 3.0 mmol), Fmoc-Gly-OH (0.892 g, 3.0 mmol), Fmoc-Lys(Boc)-OH (1.405 g, 3.0 mmol), Fmoc-Phe-OH (1.162 g, 3.0 mmol), Fmoc-Ser(tBu)-OH (1.150 g, 3.0 mmol), Fmoc-Gly-OH (0.892 g, 3.0 mmol), Fmoc-Pro-OH (1.012 g, 3.0 mmol), Fmoc-Ala-OH (0.934 g, 3.0 mmol), Fmoc-Leu-OH (1.060 g, 3.0 mmol). DMT/NMM/TosO^−^ (1.239 g, 3.0 mmol) and NMM (0.66 mL, 6.0 mmol). HPLC (10-90% A in 30 min, Method 1): *t*_R_ = 2.8 min, purity = 87%. MS: 1035.6251 ([M + H]^+^, C_47_H_79_N_12_O_12_S^+^, calc. 1034.55).

- Synthesis of ^221^GFQGEKGVKG^230^ (**5**)

Starting materials: resin (1.0 g, 1.0 mmol) and for each coupling: Fmoc-Gly-OH (0.892 g, 3.0 mmol), Fmoc-Lys(Boc)-OH (1.405 g, 3.0 mmol), Fmoc-Val-OH (1.018 g, 3.0 mmol), Fmoc-Gly-OH (0.892 g, 3.0 mmol), Fmoc-Lys(Boc)-OH (1.405 g, 3.0 mmol), Fmoc-Glu(OtBu)-OH (1.276 g, 3.0 mmol), Fmoc-Gly-OH (0.892 g, 3.0 mmol), Fmoc-Gln(Trt)-OH (1.832 g, 3.0 mmol), Fmoc-Phe-OH (1.162 g, 3.0 mmol), Fmoc-Gly-OH (0.892 g, 3.0 mmol). DMT/NMM/TosO^−^ (1.239 g, 3.0 mmol) and NMM (0.66 mL, 6.0 mmol). HPLC (30–90% A in 30 min, Method 1): *t*_R_ = 2.6 min, purity = 98%. MS: 1006.5898, 503.7991 ([M + H]^+^, C_44_H_72_N_13_O_14_^+^, [M + 2H]^2+^, calc. 1005.51).

- Synthesis of ^251^GFPKGKKGSK^260^ (**6**)

Starting materials: resin (1.0 g, 1.0 mmol) and for each coupling: Fmoc-Lys(Boc)-OH (1.405 g, 3.0 mmol), Fmoc-Ser(tBu)-OH (1.150 g, 3.0 mmol), Fmoc-Gly-OH (0.892 g, 3.0 mmol), Fmoc-Lys(Boc)-OH (1.405 g, 3.0 mmol), Fmoc-Lys(Boc)-OH (1.405 g, 3.0 mmol), Fmoc-Gly-OH (0.892 g, 3.0 mmol), Fmoc-Lys(Boc)-OH (1.405 g, 3.0 mmol), Fmoc-Pro-OH (1.012 g, 3.0 mmol), Fmoc-Phe-OH (1.162 g, 3.0 mmol), Fmoc-Gly-OH (0.892 g, 3.0 mmol). DMT/NMM/TosO^−^ (1.239 g, 3.0 mmol) and NMM (0.66 mL, 6.0 mmol). HPLC (30–90% A in 30 min, Method 1): *t*_R_ = 2.0 min, purity = 93%. MS: 1033.6762, 345.2301 ([M + H]^+^, C_47_H_81_N_14_O_12_^+^, [M + 3H]^3+^, calc. 1032.59).

- Synthesis of ^311^GPPGQQGKKG^320^ (**7**)

Starting materials: resin (1.0 g, 1.0 mmol) and for each coupling: Fmoc-Gly-OH (0.892 g, 3.0 mmol), Fmoc-Lys(Boc)-OH (1.405 g, 3.0 mmol), Fmoc-Lys(Boc)-OH (1.405 g, 3.0 mmol), Fmoc-Gly-OH (0.892 g, 3.0 mmol), Fmoc-Gln(Trt)-OH (1.832 g, 3.0 mmol), Fmoc-Gln(Trt)-OH (1.832 g, 3.0 mmol), Fmoc-Gly-OH (0.892 g, 3.0 mmol), Fmoc-Pro-OH (1.012 g, 3.0 mmol), Fmoc-Pro-OH (1.012 g, 3.0 mmol), Fmoc-Gly-OH (0.892 g, 3.0 mmol). DMT/NMM/TosO^−^ (1.239 g, 3.0 mmol) and NMM (0.66 mL, 6.0 mmol). HPLC (30–90% A in 30 min, Method 1): *t*_R_ = 2.2 min, purity = 90%. MS: 953.5674 ([M + H]^+^, C_40_H_69_N_14_O_13_^+^, calc. 952.49).

- Synthesis of ^451^NKESGFPGLR^460^ (**8**)

Starting materials: resin (1.0 g, 1.0 mmol) and for each coupling: Fmoc-Arg(Pbf)-OH (1.946 g, 3.0 mmol), Fmoc-Leu-OH (1.060 g, 3.0 mmol), Fmoc-Gly-OH (0.892 g, 3.0 mmol), Fmoc-Pro-OH (1.012 g, 3.0 mmol), Fmoc-Phe-OH (1.162 g, 3.0 mmol), Fmoc-Gly-OH (0.892 g, 3.0 mmol), Fmoc-Ser(tBu)-OH (1.150 g, 3.0 mmol), Fmoc-Glu(OtBu)-OH (1.276 g, 3.0 mmol), Fmoc-Lys(Boc)-OH (1.405 g, 3.0 mmol), Fmoc-Asn(Trt)-OH (1.790 g, 3.0 mmol). DMT/NMM/TosO^−^ (1.239 g, 3.0 mmol) and NMM (0.66 mL, 6.0 mmol). HPLC (30–90% A in 30 min, Method 1): *t*_R_ = 8.9 min, purity = 95%. MS: 552.8254 ([M + 2H]^2+^, C_48_H_78_N_15_O_15_^+^, calc. 1103.56).

- Synthesis of ^471^LKGIKGDSGF^480^ (**9**)

Starting materials: resin (1.0 g, 1.0 mmol) and for each coupling: Fmoc-Phe-OH (1.162 g, 3.0 mmol), Fmoc-Gly-OH (0.892 g, 3.0 mmol), Fmoc-Ser(tBu)-OH (1.150 g, 3.0 mmol), Fmoc-Asp(OtBu)-OH (1.234 g, 3.0 mmol), Fmoc-Gly-OH (0.892 g, 3.0 mmol), Fmoc-Lys(Boc)-OH (1.405 g, 3.0 mmol), Fmoc-Ile-OH (1.060 g, 3.0 mmol), Fmoc-Gly-OH (0.892 g, 3.0 mmol), Fmoc-Lys(Boc)-OH (1.405 g, 3.0 mmol), Fmoc-Leu-OH (1.060 g, 3.0 mmol). DMT/NMM/TosO^−^ (1.239 g, 3.0 mmol) and NMM (0.66 mL, 6.0 mmol). HPLC (30–90% A in 30 min, Method 1): *t*_R_ = 8.1 min, purity = 85%. MS: 1021.5612, 511.2875 ([M + H]^+^, C_46_H_77_N_12_O_14_^+^, [M + 2H]^2+^, calc. 1020.55).

- Synthesis of ^511^KGARGDRGSG^520^ (**10**)

Starting materials: resin (1.0 g, 1.0 mmol) and for each coupling: Fmoc-Gly-OH (0.892 g, 3.0 mmol), Fmoc-Ser(tBu)-OH (1.150 g, 3.0 mmol), Fmoc-Gly-OH (0.892 g, 3.0 mmol), Fmoc-Arg(Pbf)-OH (1.946 g, 3.0 mmol), Fmoc-Asp(OtBu)-OH (1.234 g, 3.0 mmol), Fmoc-Gly-OH (0.892 g, 3.0 mmol), Fmoc-Arg(Pbf)-OH (1.946 g, 3.0 mmol), Fmoc-Ala-OH (0.934 g, 3.0 mmol), Fmoc-Gly-OH (0.892 g, 3.0 mmol), Fmoc-Lys(Boc)-OH (1.405 g, 3.0 mmol). DMT/NMM/TosO^−^ (1.239 g, 3.0 mmol) and NMM (0.66 mL, 6.0 mmol). HPLC (30–90% A in 30 min, Method 1): *t*_R_ = 1.9 min, purity = 99%. MS: 960.5373, 480.7737, 320.8502 ([M + H]^+^, C_36_H_66_N_17_O_14_^+^, [M + 2H]^2+^, [M + 3H]^3+^, calc. 959.47).

- Synthesis of ^541^KGKKGEPILS^550^ (**11**)

Starting materials: resin (1.0 g, 1.0 mmol) and for each coupling: Fmoc-Ser(tBu)-OH (1.150 g, 3.0 mmol), Fmoc-Leu-OH (1.060 g, 3.0 mmol), Fmoc-Ile-OH (1.060 g, 3.0 mmol), Fmoc-Pro-OH (1.012 g, 3.0 mmol), Fmoc-Glu(OtBu)-OH (1.276 g, 3.0 mmol), coupling Fmoc-Gly-OH (0.892 g, 3.0 mmol), Fmoc-Lys(Boc)-OH (1.405 g, 3.0 mmol), Fmoc-Lys(Boc)-OH (1.405 g, 3.0 mmol), Fmoc-Gly-OH (0.892 g, 3.0 mmol), Fmoc-Lys(Boc)-OH (1.405 g, 3.0 mmol). DMT/NMM/TosO^−^ (1.239 g, 3.0 mmol) and NMM (0.66 mL, 6.0 mmol). HPLC (30–90% A in 30 min, Method 1): *t*_R_ = 2.9 min, purity = 85%. MS: 1056.7046, 528.8563 ([M + H]^+^, C_47_H_86_N_13_O_14_^+^, [M + 2H]^2+^, calc. 1055.62).

- Synthesis of ^641^PGQQGLPGSK^650^ (**12**)

Starting materials: resin (1.0 g, 1.0 mmol) and for each coupling: Fmoc-Lys(Boc)-OH (1.405 g, 3.0 mmol), Fmoc-Ser(tBu)-OH (1.150 g, 3.0 mmol), Fmoc-Gly-OH (0.892 g, 3.0 mmol), Fmoc-Pro-OH (1.012 g, 3.0 mmol), Fmoc-Leu-OH (1.060 g, 3.0 mmol), Fmoc-Gly-OH (0.892 g, 3.0 mmol), Fmoc-Gln(Trt)-OH (1.832 g, 3.0 mmol), Fmoc-Gln(Trt)-OH (1.832 g, 3.0 mmol), Fmoc-Gly-OH (0.892 g, 3.0 mmol), Fmoc-Pro-OH (1.012 g, 3.0 mmol). DMT/NMM/TosO- (1.239 g, 3.0 mmol) and NMM (0.66 mL, 6.0 mmol). HPLC (10-90% A in 30 min, Method 1): *t*_R_ = 2.9 min, purity = 99%. MS: 968.5737, 484.7949 ([M + H]^+^, C_41_H_70_N_13_O_14_^+^, [M + 2H]^2+^, calc. 967.49).

- Synthesis of ^671^PGFPGPKGSR^680^ (**13**)

Starting materials: resin (1.0 g, 1.0 mmol) and for each coupling: Fmoc-Arg(Pbf)-OH (1.946 g, 3.0 mmol), Fmoc-Ser(tBu)-OH (1.150 g, 3.0 mmol), Fmoc-Gly-OH (0.892 g, 3.0 mmol), Fmoc-Lys(Boc)-OH (1.405 g, 3.0 mmol), Fmoc-Pro-OH (1.012 g, 3.0 mmol), Fmoc-Gly-OH (0.892 g, 3.0 mmol), Fmoc-Pro-OH (1.012 g, 3.0 mmol), Fmoc-Phe-OH (1.162 g, 3.0 mmol), Fmoc-Gly-OH (0.892 g, 3.0 mmol), Fmoc-Pro-OH (1.012 g, 3.0 mmol). DMT/NMM/TosO^−^ (1.239 g, 3.0 mmol) and NMM (0.66 mL, 6.0 mmol). HPLC (10-90% A in 30 min, Method 2): *t*_R_ = 22.4 min, purity = 87%. MS: 999.5955, 333.8715 ([M + H]^+^, C_45_H_71_N_14_O_12_^+^, [M + 3H]^3+^, calc. 998.51).

- Synthesis of ^711^GFPGPRGEKG^720^ (**14**)

Starting materials: resin (1.0 g, 1.0 mmol) and for each coupling: Fmoc-Gly-OH (0.892 g, 3.0 mmol), Fmoc-Lys(Boc)-OH (1.405 g, 3.0 mmol), Fmoc-Glu(OtBu)-OH (1.276 g, 3.0 mmol), Fmoc-Gly-OH (0.892 g, 3.0 mmol), Fmoc-Arg(Pbf)-OH (1.946 g, 3.0 mmol), Fmoc-Pro-OH (1.012 g, 3.0 mmol), Fmoc-Gly-OH (0.892 g, 3.0 mmol), Fmoc-Pro-OH (1.012 g, 3.0 mmol), Fmoc-Phe-OH (1.162 g, 3.0 mmol), Fmoc-Gly-OH (0.892 g, 3.0 mmol). DMT/NMM/TosO^−^ (1.239 g, 3.0 mmol) and NMM (0.66 mL, 6.0 mmol). HPLC (30–90% A in 30 min, Method 1): *t*_R_ = 2.8 min, purity = 84%. MS: 1001.5502, 501.2775 ([M + H]^+^, C_44_H_69_N_14_O_13_^+^, [M + 2H]^2+^, calc. 1000.49).

- Synthesis of ^721^LPGFPGLPGK^730^ (**15**)

Starting materials: resin (1.0 g, 1.0 mmol) and for each coupling: Fmoc-Lys(Boc)-OH (1.405 g, 3.0 mmol), Fmoc-Gly-OH (0.892 g, 3.0 mmol), Fmoc-Pro-OH (1.012 g, 3.0 mmol), Fmoc-Leu-OH (1.060 g, 3.0 mmol), Fmoc-Gly-OH (0.892 g, 3.0 mmol), Fmoc-Pro-OH (1.012 g, 3.0 mmol), Fmoc-Phe-OH (1.162 g, 3.0 mmol), Fmoc-Gly-OH (0.892 g, 3.0 mmol), Fmoc-Pro-OH (1.012 g, 3.0 mmol), Fmoc-Leu-OH (1.060 g, 3.0 mmol). DMT/NMM/TosO^−^ (1.239 g, 3.0 mmol) and NMM (0.66 mL, 6.0 mmol). HPLC (30–90% A in 30 min, Method 1): *t*_R_ = 11.8 min, purity = 91%. MS: 982.6217, 491.8182 ([M + H]^+^, C_48_H_76_N_11_O_11_^+^, [M + 2H]^2+^, calc. 981.55).

- Synthesis of ^781^PGLKGVHGKP^790^ (**16**)

Starting materials: resin (1.0 g, 1.0 mmol) and for each coupling: Fmoc-Pro-OH (1.012 g, 3.0 mmol), Fmoc-Lys(Boc)-OH (1.405 g, 3.0 mmol), Fmoc-Gly-OH (0.892 g, 3.0 mmol), Fmoc-His(Trt)-OH (1.859 g, 3.0 mmol), Fmoc-Val-OH (1.018 g, 3.0 mmol), Fmoc-Gly-OH (0.892 g, 3.0 mmol), Fmoc-Lys(Boc)-OH (1.405 g, 3.0 mmol), Fmoc-Leu-OH (1.060 g, 3.0 mmol), Fmoc-Gly-OH (0.892 g, 3.0 mmol), Fmoc-Pro-OH (1.012 g, 3.0 mmol). DMT/NMM/TosO^−^ (1.239 g, 3.0 mmol) and NMM (0.66 mL, 6.0 mmol). HPLC (30–90% A in 30 min, Method 1): *t*_R_ = 2.7 min, purity = 97%. MS: 989.6496, 330.5547 ([M + H]^+^, C_45_H_77_N_14_O_11_^+^, [M + 3H]^3+^, calc. 988.57).

- Synthesis of ^821^GIKGKSGLPG^830^ (**17**)

Starting materials: resin (1.0 g, 1.0 mmol) and for each coupling: Fmoc-Gly-OH (0.892 g, 3.0 mmol), Fmoc-Pro-OH (1.012 g, 3.0 mmol), Fmoc-Leu-OH (1.060 g, 3.0 mmol), Fmoc-Gly-OH (0.892 g, 3.0 mmol), Fmoc-Ser(tBu)-OH (1.150 g, 3.0 mmol), Fmoc-Lys(Boc)-OH (1.405 g, 3.0 mmol), Fmoc-Gly-OH (0.892 g, 3.0 mmol), Fmoc-Lys(Boc)-OH (1.405 g, 3.0 mmol), Fmoc-Ile-OH (1.060 g, 3.0 mmol), Fmoc-Gly-OH (0.892 g, 3.0 mmol). DMT/NMM/TosO^−^ (1.239 g, 3.0 mmol) and NMM (0.66 mL, 6.0 mmol). HPLC (10-90% A in 30 min, Method 1): *t*_R_ = 2.8 min, purity = 99%. MS: 913.6027, 457.3051 ([M + H]^+^, C_40_H_73_N_12_O_12_^+^, [M + 2H]^2+^, calc. 912.52).

- Synthesis of ^841^PGKKGTRGKK^850^ (**18**)

Starting materials: resin (1.0 g, 1.0 mmol) and for each coupling: Fmoc-Lys(Boc)-OH (1.405 g, 3.0 mmol), Fmoc-Lys(Boc)-OH (1.405 g, 3.0 mmol), Fmoc-Gly-OH (0.892 g, 3.0 mmol), Fmoc-Arg(Pbf)-OH (1.946 g, 3.0 mmol), Fmoc-Thr(tBu)-OH (1.192 g, 3.0 mmol), Fmoc-Gly-OH (0.892 g, 3.0 mmol), Fmoc-Lys(Boc)-OH (1.405 g, 3.0 mmol), Fmoc-Lys(Boc)-OH (1.405 g, 3.0 mmol), Fmoc-Gly-OH (0.892 g, 3.0 mmol), Fmoc-Pro-OH (1.012 g, 3.0 mmol). DMT/NMM/TosO^−^ (1.239 g, 3.0 mmol) and NMM (0.66 mL, 6.0 mmol). HPLC (30–90% A in 30 min, Method 1): *t*_R_ = 2.0 min, purity = 81%. MS: 1056.7249, 352.9130 ([M + H]^+^, C_45_H_86_N_17_O_12_^+^, [M + 3H]^3+^, calc. 1055.64).

- Synthesis of ^851^GPPGSIVKKG^860^ (**19**)

Starting materials: resin (1.0 g, 1.0 mmol) and for each coupling: Fmoc-Gly-OH (0.892 g, 3.0 mmol), Fmoc-Lys(Boc)-OH (1.405 g, 3.0 mmol), Fmoc-Lys(Boc)-OH (1.405 g, 3.0 mmol), Fmoc-Val-OH (1.018 g, 3.0 mmol), Fmoc-Ile-OH (1.060 g, 3.0 mmol), Fmoc-Ser(tBu)-OH (1.150 g, 3.0 mmol), Fmoc-Gly-OH (0.892 g, 3.0 mmol), Fmoc-Pro-OH (1.012 g, 3.0 mmol), Fmoc-Pro-OH (1.012 g, 3.0 mmol), Fmoc-Gly-OH (0.892 g, 3.0 mmol). DMT/NMM/TosO^−^ (1.239 g, 3.0 mmol) and NMM (0.66 mL, 6.0 mmol). HPLC (30–90% A in 30 min, Method 1): *t*_R_ = 2.9 min, purity = 95%. MS: 939.6105, 470.3106 ([M + H]^+^, C_42_H_75_N_12_O_12_^+^, [M + 2H]^2+^, calc. 938.54).

- Synthesis of ^891^LSGPKGEKGS^900^ (**20**)

Starting materials: resin (1.0 g, 1.0 mmol) and for each coupling: Fmoc-Ser(tBu)-OH (1.150 g, 3.0 mmol), Fmoc-Gly-OH (0.892 g, 3.0 mmol), Fmoc-Lys(Boc)-OH (1.405 g, 3.0 mmol), Fmoc-Glu(OtBu)-OH (1.276 g, 3.0 mmol), Fmoc-Gly-OH (0.892 g, 3.0 mmol), Fmoc-Lys(Boc)-OH (1.405 g, 3.0 mmol), Fmoc-Pro-OH (1.012 g, 3.0 mmol), Fmoc-Gly-OH (0.892 g, 3.0 mmol), Fmoc-Ser(tBu)-OH (1.150 g, 3.0 mmol), Fmoc-Leu-OH (1.060 g, 3.0 mmol). DMT/NMM/TosO^−^ (1.239 g, 3.0 mmol) and NMM (0.66 mL, 6.0 mmol). HPLC (30–90% A in 30 min, Method 1): *t*_R_ = 2.1 min, purity = 82%. MS: 959.5691, 480.2892 ([M + H]^+^, C_40_H_71_N_12_O_15_^+^, [M + 2H]^2+^, calc. 958.49).

- Synthesis of ^921^LKGIPGSTGK^930^ (**21**)

Starting materials: resin (1.0 g, 1.0 mmol) and for each coupling: Fmoc-Lys(Boc)-OH (1.405 g, 3.0 mmol), Fmoc-Gly-OH (0.892 g, 3.0 mmol), Fmoc-Thr(tBu)-OH (1.192 g, 3.0 mmol), Fmoc-Ser(tBu)-OH (1.150 g, 3.0 mmol), Fmoc-Gly-OH (0.892 g, 3.0 mmol), Fmoc-Pro-OH (1.012 g, 3.0 mmol), Fmoc-Ile-OH (1.060 g, 3.0 mmol), Fmoc-Gly-OH (0.892 g, 3.0 mmol), Fmoc-Lys(Boc)-OH (1.405 g, 3.0 mmol), Fmoc-Leu-OH (1.060 g, 3.0 mmol). DMT/NMM/TosO^−^ (1.239 g, 3.0 mmol) and NMM (0.66 mL, 6.0 mmol). HPLC (30–90% A in 30 min, Method 1): *t*_R_ = 2.9 min, purity = 91%. MS: 957.6297, 479.3194 ([M + H]^+^, C_42_H_77_N_12_O_13_^+^, [M + 2H]^2+^, calc. 956.55).

- Synthesis of ^951^PVGIPSPRRP^960^ (**22**)

Starting materials: resin (1.0 g, 1.0 mmol) and for each coupling: Fmoc-Pro-OH (1.012 g, 3.0 mmol), Fmoc-Arg(Pbf)-OH (1.946 g, 3.0 mmol), Fmoc-Arg(Pbf)-OH (1.946 g, 3.0 mmol), Fmoc-Pro-OH (1.012 g, 3.0 mmol), Fmoc-Ser(tBu)-OH (1.150 g, 3.0 mmol), Fmoc-Pro-OH (1.012 g, 3.0 mmol), Fmoc-Ile-OH (1.060 g, 3.0 mmol), Fmoc-Gly-OH (0.892 g, 3.0 mmol), Fmoc-Val-OH (1.018 g, 3.0 mmol), Fmoc-Pro-OH (1.012 g, 3.0 mmol). DMT/NMM/TosO^−^ (1.239 g, 3.0 mmol) and NMM (0.66 mL, 6.0 mmol). HPLC (30–90% A in 30 min, Method 1): *t*_R_ = 8.5 min, purity = 92%. MS: 1075.6860, 538.3456, 359.2365 ([M + H]^+^, C_48_H_83_N_16_O_12_^+^, [M + 2H]^2+^, [M + 3H]^3+^, calc. 1074.62).

- Synthesis of ^961^MSNLWLKGDK^970^ (**23**)

Starting materials: resin (1.0 g, 1.0 mmol) and for each coupling: Fmoc-Lys(Boc)-OH (1.405 g, 3.0 mmol), Fmoc-Asp(OtBu)-OH (1.234 g, 3.0 mmol), Fmoc-Gly-OH (0.892 g, 3.0 mmol), Fmoc-Lys(Boc)-OH (1.405 g, 3.0 mmol), Fmoc-Leu-OH (1.060 g, 3.0 mmol), Fmoc-Trp(Boc)-OH (1.579 g, 3.0 mmol), Fmoc-Leu-OH (1.060 g, 3.0 mmol), Fmoc-Asn(Trt)-OH (1.790 g, 3.0 mmol), Fmoc-Ser(tBu)-OH (1.150 g, 3.0 mmol), Fmoc-Met-OH (1.114 g, 3.0 mmol). DMT/NMM/TosO^−^ (1.239 g, 3.0 mmol) and NMM (0.66 mL, 6.0 mmol). HPLC (30–90% A in 30 min, Method 1): *t*_R_ = 10.0 min, purity = 91%. MS: 1191.6721, 596.3429, 397.9016 ([M + H]^+^, C_53_H_87_N_14_O_15_S^+^, [M + 2H]^2+^, [M + 3H]^3+^, calc. 1190.60).

- Synthesis of ^1001^GAPGLPGIIK^1010^ (**24**)

Starting materials: resin (1.0 g, 1.0 mmol) and for each coupling: Fmoc-Lys(Boc)-OH (1.405 g, 3.0 mmol), Fmoc-Ile-OH (1.060 g, 3.0 mmol), Fmoc-Ile-OH (1.060 g, 3.0 mmol), Fmoc-Gly-OH (0.892 g, 3.0 mmol), Fmoc-Pro-OH (1.012 g, 3.0 mmol), Fmoc-Leu-OH (1.060 g, 3.0 mmol), Fmoc-Gly-OH (0.892 g, 3.0 mmol), Fmoc-Pro-OH (1.012 g, 3.0 mmol), Fmoc-Ala-OH (0.934 g, 3.0 mmol), Fmoc-Gly-OH (0.892 g, 3.0 mmol). DMT/NMM/TosO^−^ (1.239 g, 3.0 mmol) and NMM (0.66 mL, 6.0 mmol). HPLC (30–90% A in 30 min, Method 1): *t*_R_ = 10.0 min, purity = 80%. MS: 922.6209, 461.8178 ([M + H]^+^, C_43_H_76_N_11_O_11_^+^, [M + 2H]^2+^, calc. 921.55).

- Synthesis of ^1091^FKGTKGRDGL^1100^ (**25**)

Starting materials: resin (1.0 g, 1.0 mmol) and for each coupling: Fmoc-Leu-OH (1.060 g, 3.0 mmol), Fmoc-Gly-OH (0.892 g, 3.0 mmol), Fmoc-Asp(OtBu)-OH (1.234 g, 3.0 mmol), Fmoc-Arg(Pbf)-OH (1.946 g, 3.0 mmol), Fmoc-Gly-OH (0.892 g, 3.0 mmol), Fmoc-Lys(Boc)-OH (1.405 g, 3.0 mmol), Fmoc-Thr(tBu)-OH (1.192 g, 3.0 mmol), Fmoc-Gly-OH (0.892 g, 3.0 mmol), Fmoc-Lys(Boc)-OH (1.405 g, 3.0 mmol), Fmoc-Phe-OH (1.162 g, 3.0 mmol). DMT/NMM/TosO^−^ (1.239 g, 3.0 mmol) and NMM (0.66 mL, 6.0 mmol). HPLC (30–90% A in 30 min, Method 1): *t*_R_ = 2.8 min, purity = 92%. MS: 1078.6536, 360.2275 ([M + H]^+^, C_47_H_80_N_15_O_14_^+^, [M + 3H]^3+^, calc. 1077.58).

- Synthesis of ^1101^IGNIGFPGNK^1110^ (**26**)

Starting materials: resin (1.0 g, 1.0 mmol) and for each coupling: Fmoc-Lys(Boc)-OH (1.405 g, 3.0 mmol), Fmoc-Asn(Trt)-OH (1.790 g, 3.0 mmol), Fmoc-Gly-OH (0.892 g, 3.0 mmol), Fmoc-Pro-OH (1.012 g, 3.0 mmol), Fmoc-Phe-OH (1.162 g, 3.0 mmol), Fmoc-Gly-OH (0.892 g, 3.0 mmol), Fmoc-Ile-OH (1.060 g, 3.0 mmol), Fmoc-Asn(Trt)-OH (1.790 g, 3.0 mmol), Fmoc-Gly-OH (0.892 g, 3.0 mmol), Fmoc-Ile-OH (1.060 g, 3.0 mmol). DMT/NMM/TosO^−^ (1.239 g, 3.0 mmol) and NMM (0.66 mL, 6.0 mmol). HPLC (30–90% A in 30 min, Method 1): *t*_R_ = 9.6 min, purity = 80%. MS: 1016.6166, 508.8136 ([M + H]^+^, C_46_H_74_N_13_O_13_^+^, [M + 2H]^2+^, calc. 1015.53).

- Synthesis of ^1211^PGIGIGAPGK^1220^ (**27**)

Starting materials: resin (1.0 g, 1.0 mmol) and for each coupling: Fmoc-Lys(Boc)-OH (1.405 g, 3.0 mmol), Fmoc-Gly-OH (0.892 g, 3.0 mmol), Fmoc-Pro-OH (1.012 g, 3.0 mmol), Fmoc-Ala-OH (0.934 g, 3.0 mmol), Fmoc-Gly-OH (0.892 g, 3.0 mmol), Fmoc-Ile-OH (1.060 g, 3.0 mmol), Fmoc-Gly-OH (0.892 g, 3.0 mmol), Fmoc-Ile-OH (1.060 g, 3.0 mmol), Fmoc-Gly-OH (0.892 g, 3.0 mmol), Fmoc-Pro-OH (1.012 g, 3.0 mmol). DMT/NMM/TosO^−^ (1.239 g, 3.0 mmol) and NMM (0.66 mL, 6.0 mmol). HPLC (30–90% A in 30 min, Method 1): *t*_R_ = 8.8 min, purity = 95%. MS: 866.5585, 433.7870 ([M + H]^+^, C_39_H_68_N_11_O_11_^+^, [M + 2H]^2+^, calc. 865.49).

- Synthesis of ^1221^PGLRGQKGDR^1230^ (**28**)

Starting materials: resin (1.0 g, 1.0 mmol) and for each coupling: Fmoc-Arg(Pbf)-OH (1.946 g, 3.0 mmol), Fmoc-Asp(OtBu)-OH (1.234 g, 3.0 mmol), Fmoc-Gly-OH (0.892 g, 3.0 mmol), Fmoc-Lys(Boc)-OH (1.405 g, 3.0 mmol), Fmoc-Gln(Trt)-OH (1.832 g, 3.0 mmol), Fmoc-Gly-OH (0.892 g, 3.0 mmol), Fmoc-Arg(Pbf)-OH (1.946 g, 3.0 mmol), Fmoc-Leu-OH (1.060 g, 3.0 mmol), Fmoc-Gly-OH (0.892 g, 3.0 mmol), Fmoc-Pro-OH (1.012 g, 3.0 mmol). DMT/NMM/TosO^−^ (1.239 g, 3.0 mmol) and NMM (0.66 mL, 6.0 mmol). HPLC (30–90% A in 30 min, Method 1): *t*_R_ = 2.0 min, purity = 80%. MS: 1083.6613, 361.8937 ([M + H]^+^, C_44_H_79_N_18_O_14_^+^, [M + 3H]^3+^, calc. 1082.58).

- Synthesis of ^1311^KGMRGEPGFM^1320^ (**29**)

Starting materials: resin (1.0 g, 1.0 mmol) and for each coupling: Fmoc-Met-OH (1.114 g, 3.0 mmol), Fmoc-Phe-OH (1.162 g, 3.0 mmol), Fmoc-Gly-OH (0.892 g, 3.0 mmol), Fmoc-Pro-OH (1.012 g, 3.0 mmol), Fmoc-Glu(OtBu)-OH (1.276, 3.0 mmol), Fmoc-Gly-OH (0.892 g, 3.0 mmol), Fmoc-Arg(Pbf)-OH (1.946 g, 3.0 mmol), Fmoc-Met-OH (1.114 g, 3.0 mmol), Fmoc-Gly-OH (0.892 g, 3.0 mmol), Fmoc-Lys(Boc)-OH (1.405 g, 3.0 mmol). DMT/NMM/TosO^−^ (1.239 g, 3.0 mmol) and NMM (0.66 mL, 6.0 mmol). HPLC (30–90% A in 30 min, Method 1): *t*_R_ = 9.3 min, purity = 92%. MS: 1109.5893, 555.3018 ([M + H]^+^, C_47_H_77_N_14_O_13_S_2_^+^, [M + 2H]^2+^, calc. 1108.51).

- Synthesis of ^1331^DPGFPGMKGK^1340^ (**30**)

Starting materials: resin (1.0 g, 1.0 mmol) and for each coupling: Fmoc-Lys(Boc)-OH (1.405 g, 3.0 mmol), Fmoc-Gly-OH (0.892 g, 3.0 mmol), Fmoc-Lys(Boc)-OH (1.405 g, 3.0 mmol), Fmoc-Met-OH (1.114 g, 3.0 mmol), Fmoc-Gly-OH (0.892 g, 3.0 mmol), Fmoc-Pro-OH (1.012 g, 3.0 mmol), Fmoc-Phe-OH (1.162 g, 3.0 mmol), Fmoc-Gly-OH (0.892 g, 3.0 mmol), Fmoc-Pro-OH (1.012 g, 3.0 mmol), Fmoc-Asp(OtBu)-OH (1.234 g, 3.0 mmol). DMT/NMM/TosO^−^ (1.239 g, 3.0 mmol) and NMM (0.66 mL, 6.0 mmol). HPLC (30–90% A in 30 min, Method 1): *t*_R_ = 8.5 min, purity = 92%. MS: 1033.5643, 517.2877 ([M + H]^+^, C_46_H_73_N_12_O_13_S^+^, [M + 2H]^2+^, calc. 1032.49).

- Synthesis of ^1451^MRVGYTLVKH^1460^ (**31**)

Starting materials: resin (1.0 g, 1.0 mmol) and for each coupling: Fmoc-His(Trt)-OH (1.859 g, 3.0 mmol), Fmoc-Lys(Boc)-OH (1.405 g, 3.0 mmol), Fmoc-Val-OH (1.018 g, 3.0 mmol), Fmoc-Leu-OH (1.060 g, 3.0 mmol), Fmoc-Thr(tBu)-OH (1.192 g, 3.0 mmol), Fmoc-Tyr(tBu)-OH (1.379 g, 3.0 mmol), Fmoc-Gly-OH (0.892 g, 3.0 mmol), Fmoc-Val-OH (1.018 g, 3.0 mmol), Fmoc-Arg(Pbf)-OH (1.946 g, 3.0 mmol), Fmoc-Met-OH (1.114 g, 3.0 mmol). DMT/NMM/TosO^−^ (1.239 g, 3.0 mmol) and NMM (0.66 mL, 6.0 mmol). HPLC (30–90% A in 30 min, Method 1): *t*_R_ = 8.3 min, purity = 80%. MS: 1203.6398, 401.8919 ([M + H]^+^, C_54_H_91_N_16_O_13_S^+^, [M + 3H]^3+^, calc. 1202.65).

- Synthesis of ^1521^HYARRNDKSY^1530^ (**32**)

Starting materials: resin (1.0 g, 1.0 mmol) and for each coupling: Fmoc-Tyr(tBu)-OH (1.379 g, 3.0 mmol), Fmoc-Ser(tBu)-OH (1.150 g, 3.0 mmol), Fmoc-Lys(Boc)-OH (1.405 g, 3.0 mmol), Fmoc-Asp(OtBu)-OH (1.234 g, 3.0 mmol), Fmoc-Asn(Trt)-OH (1.790 g, 3.0 mmol), Fmoc-Arg(Pbf)-OH (1.946 g, 3.0 mmol), Fmoc-Arg(Pbf)-OH (1.946 g, 3.0 mmol), Fmoc-Ala-OH (0.934 g, 3.0 mmol), Fmoc-Tyr(tBu)-OH (1.379 g, 3.0 mmol), Fmoc-His(Trt)-OH (1.859 g, 3.0 mmol). DMT/NMM/TosO^−^ (1.239 g, 3.0 mmol) and NMM (0.66 mL, 6.0 mmol). HPLC (30–90% A in 30 min, Method 1): *t*_R_ = 1.9 min, purity = 82%. MS: 1309.6100, 655.3173, 437.2168 ([M + H]^+^, C_56_H_85_N_20_O_17_^+^, [M + H]^2+^, [M + 3H]^3+^, calc. 1308,62).

- Synthesis of ^1661^AGQLHTRVSR^1670^ (**33**)

Starting materials: resin (1.0 g, 1.0 mmol) and for each coupling: Fmoc-Arg(Pbf)-OH (1.946 g, 3.0 mmol), Fmoc-Ser(tBu)-OH (1.150 g, 3.0 mmol), Fmoc-Val-OH (1.018 g, 3.0 mmol), Fmoc-Arg(Pbf)-OH (1.946 g, 3.0 mmol), Fmoc-Thr(tBu)-OH (1.192 g, 3.0 mmol), Fmoc-His(Trt)-OH (1.859 g, 3.0 mmol), Fmoc-Leu-OH (1.060 g, 3.0 mmol), Fmoc-Gln(Trt)-OH (1.832 g, 3.0 mmol), Fmoc-Gly-OH (0.892 g, 3.0 mmol), Fmoc-Ala-OH (0.934 g, 3.0 mmol). DMT/NMM/TosO^−^ (1.239 g, 3.0 mmol) and NMM (0.66 mL, 6.0 mmol). HPLC (30–90% A in 30 min, Method 1): *t*_R_ = 2.0 min, purity = 92%. MS: 1124.6773, 562.8491, 375.5688 ([M + H]+, C_46_H_82_N_19_O_14_^+^, [M + H]^2+^, [M + 3H]^3+^, calc. 1123.61).

- Synthesis of ^23^EKSYGKPCGGQDC^35^ (**2.1**)

Starting materials: resin (1.0 g, 1.0 mmol) and for each coupling: Fmoc-Cys(Trt)-OH (1.757 g, 3.0 mmol), Fmoc-Asp(OtBu)-OH (1.234 g, 3.0 mmol), Fmoc-Gln(Trt)-OH (1.832 g, 3.0 mmol), Fmoc-Gly-OH (0.892 g, 3.0 mmol), Fmoc-Gly-OH (0.892 g, 3.0 mmol), Fmoc-Cys(Trt)-OH (1.757 g, 3.0 mmol), Fmoc-Pro-OH (1.012 g, 3.0 mmol), Fmoc-Lys(Boc)-OH (1.405 g, 3.0 mmol), Fmoc-Gly-OH (0.892 g, 3.0 mmol), Fmoc-Tyr(tBu)-OH (1.379 g, 3.0 mmol), Fmoc-Ser(tBu)-OH (1.150 g, 3.0 mmol), Fmoc-Lys(Boc)-OH (1.405 g, 3.0 mmol), Fmoc-Glu(OtBu)-OH (1.276 g, 3.0 mmol). DMT/NMM/TosO^−^ (1.239 g, 3.0 mmol) and NMM (0.66 mL, 6.0 mmol). HPLC (30–90% A in 30 min, Method 1): *t*_R_ = 0.9 min, purity = 92%. MS: 1372.5602 ([M + H]^+^, C_55_H_87_N_16_O_21_S_2_^+^, calc. 1371.52).

- Synthesis of ^1446^MPGQSMRVGYTL^1457^ (**2.2**)

Starting materials: resin (1.0 g, 1.0 mmol) and for each coupling: Fmoc-Leu-OH (1.060 g, 3.0 mmol), Fmoc-Thr(tBu)-OH (1.192 g, 3.0 mmol), Fmoc-Tyr(tBu)-OH (1.379 g, 3.0 mmol), Fmoc-Gly-OH (0.892 g, 3.0 mmol), Fmoc-Val-OH (1.018 g, 3.0 mmol), Fmoc-Arg(Pbf)-OH (1.946 g, 3.0 mmol), Fmoc-Met-OH (1.114 g, 3.0 mmol), Fmoc-Ser(tBu)-OH (1.150 g, 3.0 mmol), Fmoc-Gln(Trt)-OH (1.832 g, 3.0 mmol), Fmoc-Gly-OH (0.892 g, 3.0 mmol), Fmoc-Pro-OH (1.012 g, 3.0 mmol), Fmoc-Met-OH (1.114 g, 3.0 mmol). DMT/NMM/TosO^−^ (1.239 g, 3.0 mmol) and NMM (0.66 mL, 6.0 mmol). HPLC (30–90% A in 30 min, Method 1): *t*_R_ = 11.4 min, purity = 99%. MS: 1340.6140 ([M + H]^+^, C_57_H_95_N_16_O_17_S_2_^+^, calc. 1339.60).

- Synthesis of ^1453^VGYTLVKHSQSE^1464^ (**2.3**)

Starting materials: resin (1.0 g, 1.0 mmol) and for each coupling: Fmoc-Glu(OtBu)-OH (1.276 g, 3.0 mmol), Fmoc-Ser(tBu)-OH (1.150 g, 3.0 mmol), Fmoc-Gln(Trt)-OH (1.832 g, 3.0 mmol), Fmoc-Ser(tBu)-OH (1.150 g, 3.0 mmol), Fmoc-His(Trt)-OH (1.859 g, 3.0 mmol), Fmoc-Lys(Boc)-OH (1.405 g, 3.0 mmol), Fmoc-Val-OH (1.018 g, 3.0 mmol), Fmoc-Leu-OH (1.060 g, 3.0 mmol), Fmoc-Thr(tBu)-OH (1.192 g, 3.0 mmol), Fmoc-Tyr(tBu)-OH (1.379 g, 3.0 mmol), Fmoc-Gly-OH (0.892 g, 3.0 mmol), Fmoc-Val-OH (1.018 g, 3.0 mmol). DMT/NMM/TosO^−^ (1.239 g, 3.0 mmol) and NMM (0.66 mL, 6.0 mmol). HPLC (30–90% A in 30 min, Method 1): *t*_R_ = 2.6 min, purity = 99%. MS: 1349.6571 ([M + H]^+^, C_59_H_95_N_16_O_20_+, calc. 1347.49).

- Synthesis of ^1520^CHYARRNDKSYW^1531^ (**2.4**)

Starting materials: resin (1.0 g, 1.0 mmol) and for each coupling: Fmoc-Trp(Boc)-OH (1.579 g, 3.0 mmol), Fmoc-Tyr(tBu)-OH (1.379 g, 3.0 mmol), Fmoc-Ser(tBu)-OH (1.150 g, 3.0 mmol), Fmoc-Lys(Boc)-OH (1.405 g, 3.0 mmol), Fmoc-Asp(OtBu)-OH (1.234 g, 3.0 mmol), Fmoc-Asn(Trt)-OH (1.790 g, 3.0 mmol), Fmoc-Arg(Pbf)-OH (1.946 g, 3.0 mmol), Fmoc-Arg(Pbf)-OH (1.946 g, 3.0 mmol), Fmoc-Ala-OH (0.934 g, 3.0 mmol), Fmoc-Tyr(tBu)-OH (1.379 g, 3.0 mmol), Fmoc-His(Trt)-OH (1.859 g, 3.0 mmol), Fmoc-Cys(Trt)-OH (1.757 g, 3.0 mmol). DMT/NMM/TosO^−^ (1.239 g, 3.0 mmol) and NMM (0.66 mL, 6.0 mmol). HPLC (30–90% A in 30 min, Method 1): *t*_R_ = 2.8 min, purity = 99%. MS: 1600.6767 ([M + H]^+^, C_70_H_100_N_23_O_19_S^+^, calc. 1598.77).

### 3.3. CD Studies of Peptides **1**–**33** and **2.1**–**2.4**

The CD spectra were recorded using a Jasco J-1500 instrument (Jasco, Cracow, Poland) and quartz cuvettes with 1 mm optical path lengths. The measurements were acquired in the range of 190–270 nm at temperatures of 4 °C, 20 °C and 40 °C by taking points every 5 nm, with a scan rate of 100 nm per min, an integration time of 1 s, and a bandwidth of 4 nm. The samples were prepared in MiliQ purity water with a final concentration of 0.1 mg/mL. Samples before CD measurements were incubated for 24 h. at 4 °C, 20 °C and 40 °C using a Lauda thermostat.

### 3.4. Microscopic Examination of Aggregates Formed by Collagen IV Fragments

- Microscopic Examination of Collagen IV Fragments Stained with Congo Red

In order to initiate aggregation, peptides were dissolved in a phosphate buffer solution of pH 7.2 and a concentration of 250 μM (approx. 3.5 mg of peptide per 1 mL of solution) and incubated at 37.5 °C for 7 days.

Congo Red solution with a concentration of 250 μM was prepared by dissolving the dye (Sigma-Aldrich) (17.42 mg; 0.025 mmol) in phosphate buffer (pH = 7.2) (10 mL) with the following composition: 100 mM NaH_2_PO_4_/Na_2_HPO_4_, 137 mM NaCl and 10% EtOH. Ethyl alcohol was added to the buffer to counteract the formation of dye micelles. A sample for microscopic analysis (peptide solution) was centrifuged at 12,000–14,000 rpm to precipitate. In the next step, the solution was decanted and the residue was washed three times with water to remove excess dye. Then, a suspension of the peptide in water was applied to a microscope slide. The samples were dried in a stream of air at room temperature and analyzed under polarized light.

The obtained structures were analyzed using a Delta Optical Genetic Pro (Warsaw, Poland) light microscope.

- AFM Studies of the Ability of Peptide 8 to Aggregation

Experiments were made using an NT-MDT AFM Solver apparatus. Specimens were prepared using the drop-casting technique with a Si wafer as a base. The choice of the substratum was dictated by the need for a perfectly flat, non-reactive and inert for examined peptides specimen. The peptides were left to dry for 12 h at room temperature and after that AFM the semi-contact mode was used. No chemical or oven drying was used to prevent peptide denaturation or organization different from spontaneous.

- SEM Studies of the Porous Materials

SEM tests of porous materials were performed using a high-resolution scanning electron microscope (FEI NOVA NanoSEM 230) with an EDS X-ray microanalyzer (EDAX Apollo SDD) The investigations were carried out under low vacuum conditions (0.7 mbar) with electron beam energy 10 keV. A typical procedure for the preparation of samples was as follow: approximately 0.2 g of the sample that had been obtained in the freeze-drying process, was cut with a sharp blade, forming a thin flat layer [92]. Test samples were prepared by mounting them to pin stubs using carbon conductive adhesive tape, without being covered with gold, this type of SEM microscopy allows to observe samples in their natural state, showing their real structure.

### 3.5. Studies on the Ability of Collagen IV Fragments to Interact with ITGα1β1 by Using MST Method

- Preparation of Materials for MST Analysis
  (1) ITGα1β1—a solution with a concentration of 9.3 µM was prepared. For this, 50 µL of sterile PBS (filtered through a 0.22 µm filter) was added to the protein. The solution was divided into 4 µL aliquots. The protein was His-tag labeled at C-end.
  (2) Marker/Dye—100 µg (HIS Lite™ OG488-Tris NTA-Ni Complex (AAT Bioquest, Inc., Sunnyvale, CA, USA) https://www.aatbio.com/products/his-lite-og488-tris-nta-ni-complex?unit=12615 (accessed on 22 April 2021)) was dissolved in 54.4 µL of H_2_O to obtain a 1 mM solution. The solution was divided into 2 µL (stock solution) aliquots. To obtain a 5 µM stock solution, 398 µL PBST was added to 2 µL of dye. The fluorescent dye OG488 (ex = 498 nm, ex = 526 nm) provide an efficient method for site-specific and stable noncovalent fluorescence labeling of polyhistidine-tagged proteins.
  (3) A 0.05% Tween20 solution in PBS (PBST) was prepared.
- Testing the Affinity of the Dye to the Protein

In the first step, 8.6 µL of 9.3 µM ITGα1β1 solution was added to 7.1 µL PBST and then 4.3 µL of 0.05% Tween20 solution was added to equilibrate the concentration. A 4 µM solution of ITGα1β1 in PBST (with Tween20 content = 0.05%) was obtained. In parallel, a 5 µM dye solution was prepared by mixing 2 µL of a 1 mM solution (stock) with 398 µL of PBST. Then, 2 µL was taken from the thus prepared solution and added to 198 µL of PBST to obtain a solution with a concentration of 50 nM. PBST (10 µL) was added to test tubes 2–16 (16 pieces), 20 µL of the prepared protein solution was transferred to test tube 1, and a dilution series was prepared by pipetting 10 µL from tube 1 to 2, then from 2 to 3, continuing to tube 16. After receiving the dilution series, the previously prepared dye solution was added to each 10 µL test tubes, resulting in a final concentration of 25 nM. The samples were incubated for 30 min and then measured using 40% excitation LED power and medium (40%) MST power in standard glass capillaries at 22;C temperature. Fluorescence intensity was controlled by capillary scan before each MST assay.

- Protein Labeling before Studying the Ability to Interact with Peptides

A total of 368 µL of PBST was added to the stock solution of ITGα1β1 (4 µL, 9.3 µM) to give a 100 nM solution. Then 3.7 µL of the stock dye solution (5 µM) was taken and added to 366.3 µL of PBST (final concentration—50 nM). In the next step, 370 µL of both solutions were mixed (the molar ratio protein:dye was 2:1) and incubated for 30 min. After this time, the sample was centrifuged and the supernatant was collected. The labeled protein (12 µL) was mixed with 12 µL of PBST and a pre-test was performed to validate the process according to methodology instructions (NanoTemper Technology).

- Studies of the Affinity of Peptides to ITGα1β1

Peptide solutions were prepared with a concentration 2× higher than the target concentration in the assay. Test tubes (16 pieces) were prepared. PBST (7 µL) was added to tubes 2–16, 14 µL, the prepared peptide solution was transferred to 1 tube, and a series of dilutions was made by pipetting 7 µL from tube 1 to 2, then from 2 to 3, continuing to tube 16. After receiving the dilution series, 7 µL of the labeled protein (50 nM) was added to each tube, resulting in a final concentration of 25 nM. The samples were incubated for 30 min and then measurements were taken at 80% excitation LED power (100% for peptide **4**) and medium (40%) MST power in standard glass capillaries at 22 °C temperature. The MonolithTM NT.115 BLUE/GREEN instrument was used in the tests (NanoTemper Technologies) using the MO. Control v2.2.4 software. The signals of 4 s MST-on time (from five independent measurements were analyzed using the MO Affinity Analysis software (version 2.2.4, NanoTemper Technologies). For each experiment, several quality parameters were quantified automatically (sample aggregation, surface adsorption, photobleaching, fluorescence intensity and homogeneity) and MST conditions were determined for an optimal signal-to-noise ratio for K_d_ extraction. The data were automatically fit and a K_d_ was extracted.

### 3.6. Preparation of Porous Materials from Collagen IV Derivatives

- Preparation of porous materials from ^1091^FKGTKGRDGL^1100^ (25) and ^851^GPPGSIVKKG^860^ (19)

For obtaining porous materials from peptides **25** and **19**, 100 mg of each peptide was dissolved in 2 mL of MiliQ water and solid, finely shredded solid CO_2_ was added (100 mg). The vigorously stirred solution was frozen in liquid nitrogen and freeze-dried. The obtained structures were analyzed using a Delta Optical Genetic Pro (Warsaw, Poland) light microscope.

- Preparation of porous materials with an equimolar mixture of peptides **2, 4**, **5**, **6**, **14, 15**, **25**, **26** and **30**

The first step involved the preparation of the porous material. For this purpose, two solutions were prepared containing 0.1 µmol of each peptide in 30 mL of MiliQ water. Dry ice (3.0 g) was added to one of the solutions. The solutions were frozen in liquid nitrogen and freeze-dried. In the next step, the obtained materials were cross-linked with DMT/NMM/TosO^−^. Two 80% ethanol in water solutions were prepared and DMT/NMM/TosO^−^ (2.478 g, 6 mmol) was added. The freeze-dried solids were immersed in the cross-linking solution for 1 h. At this time, the solution was filtered and the material was washed sequentially with 0.1 M Na_2_HPO_4_ and then with deionized water. Finally, the materials were frozen and freeze-dried.

### 3.7. Biological Activity Studies

- Cell Culture

All experiments on the analysis of the biological properties of the active peptides were performed on a commercially available human fibroblast cell line—BJ (ATCC^®^ CRL-2522™), BJ-5ta (ATCC^®^ CRL-4001™) and mouse myoblast—C2C12 (ATCC^®^ CRL-1772™) (ATCC; Manassas, VA, USA). The selection of cell lines was based on the mechanism of action and potential regenerative properties of selected fragments of collagen IV. Due to the potential use of collagen IV fragments in regenerative medicine in the healing of tissue after an injury but also restoring damaged tissues to their native function, cell lines used in the article are normal and concern skin and muscle tissue. Both human and mouse cell lines were used because both models represent the mammalian model and show tissue morphological similarity. Cell lines were cultured under standard conditions (5% CO_2_, 95% humidity, 37 °C). The culture medium for the BJ line Eagle’s Minimum Essential Medium (EMEM) was supplemented with 4 mM L-glutamine, and fetal bovine serum, 10%. For BJ-5ta Dulbecco’s Modified Eagle Medium (DMEM) and Medium 199 in ratio 4:1 and fetal bovine serum, 10% were used. For C2C12 Dulbecco’s Modified Eagle Medium (DMEM) and fetal bovine serum, 10% were applied.

- Cell Viability

Cell viability level was estimated by using in vitro toxicology assay kit, resazurin-based. The in vitro toxicology assay kit, resazurin-based, belongs to the colorimetric tests. It contains the key compound resazurin as an indicator of redox. The amount of red intermediate pigment (resorufin) that is obtained from resazurin reduction is directly proportional to the number of viable cells. Due to the high sensitivity of this assay, it is ideally suited for measuring the cytotoxicity of compounds. Cells were plated and incubated in 96-well plates (8 × 10^3^ cells per well). In order to assess the cytotoxicity of the compounds, a number of dilutions were made in the range from 50 μM to 1.5 μM. Cells were then incubated with compounds for 48 h. Untreated cells comprised a negative control, whereas cells incubated with 100% DMSO comprised a positive control. All of the analyzed samples and controls were then incubated with collagen IV fragments for 48 h. Following incubation of cells, the well contents were removed and wells were rinsed with 1 X DPBS. Next, 100 μL of the resazurin solution (10% of cell culture medium volume) was added to each well and incubated with the resazurin solution for 2 h. Absorbance was measured at a wavelength of 600 nm and at a reference wavelength of 690 nm using a Synergy HT (BioTek) spectrophotometer [93]. Cell viability was calculated using the following formula:

$$\mathrm{Cell}\;\mathrm{viability}\;\left(\%\right)=\frac{A_{sample}\;\left(A_{600}-A_{690}\right)-A_{blank}\;\left(A_{600}-A_{690}\right)}{A_{control}\;\left(A_{600}-A_{690}\right)-A_{blank}\;\left(A_{600}-A_{690}\right)}\times 100$$

- Genotoxicity Analysis

Genotoxicity assessment was performed using the alkaline version of the comet assay. The comet assay is used to assess the level of DNA damage at the level of individual cells. The comet assay allows the evaluation of the kinetics of the resulting DNA damage. The assay consists of performing single electrophoresis of cells in an agarose gel that has previously been lysed and denatured. As a result of the assay, in a fluorescence microscope, comets are observed, consisting of a “head”, which is part of undamaged DNA, and a “tail”—fragmented DNA resulting from genotoxic compounds. The alkaline version of the comet assay allows the assessment of oxidative damage, single- and double-stranded cracks and alkaline labile sites. Cells were plated and incubated in 12-well plates (2 × 10^5^/well) and cultured in 1 mL of complete EMEM medium for 24 h. Untreated cells containing the resazurin solution (10% of cell culture medium volume) comprised a negative control, whereas cells incubated with 10% DMSO comprised a positive control. After cell adhesion, cells were incubated with active peptides for 48 h. After incubating the cells, the contents of the wells were removed and then 0.3 mL of accutase/well was added to harvest the cells. The harvested cells were centrifuged and suspended in 0.37% Low melting point (LMP) agarose. The pellet suspended in 0.37% LMP agarose was applied to slides previously coated with NMP agarose. Then the obtained preparations were incubated with lysis buffer at pH 10 with 1% X-100 Triton for 1 h at 4 °C. After lysis, cells were incubated with the development buffer for 20 min at 4 °C and electrophoresed (17V, 32mA, 20 min) in an electrophoretic buffer. Next, the slides were rinsed three times with distilled water and allowed to dry completely. The obtained preparations were stained with the DAPI fluorescent dye and analyzed under a fluorescence microscope. The level of DNA damage was estimated using the Lucia Comet Assay software (Laboratory Imaging, s.r.o.) [94].

- Statistical Analysis

Statistical analysis was carried out using the Sigma Plot program (Systat Software, Inc., San Jose, CA, USA). For each analysis of the tests, the normality test was performed using the Shapiro-Wilk test. In the case of the analysis of the cell viability level, a normal distribution was obtained, therefore the statistical analysis between the two groups was performed using the Student’s T-test. For the comet assay analysis, no normal distribution was obtained, therefore the statistical analysis between two groups was performed using the Mann-Whitney rank-sum test. Each of the analyses in individual experiments was based on the results of three independent tests. In the graphs, the differences were statistically significant as follows: * *p* < 0.05, ** *p* < 0.01, *** *p* < 0.001.

- Cell Culture

All experiments on biological properties were performed on a commercially available monocyte/macrophage peripheral blood cell line—SC (ATCC CRL-9855) (ATCC; Manassas, VA, USA). Cell lines were cultured under standard conditions (5% CO_2_, 95% humidity, 37 °C). The culture medium for the SC line was Iscove’s Modified Dulbecco’s Medium (IMDM) with: 4 mM L-glutamine, 1.5 g/L sodium bicarbonate, 0.05 mM 2-mercaptoethanol, 0.1 mM hypoxanthine and 0.016 mM thymidine, 90%; fetal bovine serum, 10%.

- Human Inflammation Panel Assay

The level of inflammatory responses, mediated by various cytokines and chemokines induced by collagen oligopeptides was assessed using LEGENDplex™ Human Inflammation Panel 1 assay. Cells were plated and incubated with collagen oligopeptides in 24-well plates (1 × 10^6^ cells per well) for 48 h. After the incubation period, LEGENDplex™ Human Inflammation Panel 1 assay was performed. The level of cytokines and chemokines was measured using a flow cytometer.

- Secondary Structure Degradation and Enzymatic Hydrolysis of Collagen IV

Native Human Collagen IV protein (Abcam, ab7536) and Recombinant Human Collagen IV alpha 6 protein (Abcam, ab158158) were used in the studies. The degradations were carried out in accordance with the literature procedure [95,96]. Native Human Collagen IV (1 mg) and Recombinant Human Collagen IV alpha 6 (1 mg) were suspended in 0.5 mL MiliQ water and homogenized for 1 min at room temperature. The solution was then heated to 80 °C for 10 min. After cooling to 50 °C, the pH of the mixture was adjusted to 8 using 0.1 M NaOH and the volume was made up to 1.5 mL by adding water with a pH value previously adjusted to 8.

The next step was enzymatic hydrolysis with trypsin (Trypsin from porcine pancreas, Type IX-S, lyophilized powder, 13,000–20,000 BAEE units/mg protein, Sigma-Aldrich). For both protein, the enzyme to substrate ratio was set to 1:50 (wt/wt). Collagen IV was hydrolyzed for 24 h under continuous shaking at 250 rpm at 50 °C. For keeping constant pH during the hydrolysis process, pH-stat titrator was used. After hydrolysis, solution was heated to 80 °C for 10 min, and then cooled to room temperature. The last step was centrifugation at 20,000× *g*, 10 min, at 4 °C. The supernatant with soluble collagen IV peptides was frozen and lyophilizated.

- Examination of cytotoxicity of cross-linked porous materials based on the equimolar mixture of peptides **2**, **4**, **5**, **6**, **14**, **15**, **25**, **26** and **30**

The MTT test was performed using BJ (ATCC^®^ CRL-2522™) (ATCC, Manassas, VA, USA). The extracts (2 mg/mL) were incubated in DMEM (Corning) for 48 h at 37 °C. The cells were seeded into a 96-well plate with 1 × 10^4^ cells per well and grown under standard conditions (37 °C, 5% CO_2_ in humid air). The next day, the culture medium was replaced with extracts. After 1 day and 7 days of treatment, the culture medium was removed and MTT solution was added replaced with the (1 mg/mL, Sigma Aldrich, Poznan, Poland) and incubated for 2 h in an incubator. The MTT solution was then decanted and 100 µL of isopropanol (Sigma Aldrich, Poznan, Poland) was added to each well. Absorbance was measured at 570 nm and at 650 nm for the reference using a microplate reader (Victor X4 Perkin Elmer, PerkinElmer, Inc. Waltham, MA, USA). Cells cultured for 48 h in DMEM were used as a negative control. As a positive control, we used cells incubated for 24 h followed by the addition of DMSO, which were then cultured for another 24 h. Statistical significance was assessed using one-way ANOVA analysis of variance. Values for which the value *** *p* < 0.001, ** *p* < 0.01, * *p* < 0.05 were considered statistically significant.

## 4. Conclusions

It was found that out of the pool of 37 fragments (peptides **1**–**33** and **2.1**–**2.4**) reconstructing the outer sphere of collagen IV (CO4A6_HUMAN Q14031 Collagen alpha-6(IV) chain), nine fragments (peptides: ^41^CFPEKGARGR^50^ **2**, ^161^LAPGSFKGMK^170^ **4**, ^221^GFQGEKGVKG^230^ **5**, ^251^GFPKGKKGSK^260^ **6**, **711**GFPGPRGEKG**720 14**, ^721^LPGFPGLPGK^730^ **15**, ^1091^FKGTKGRDGL^1100^ **25**, ^1101^IGNIGFPGNK^1110^ **26** and ^1331^DPGFPGMKGK^1340^ **30**), due to self-assembling, form structures that mimic the structure of the triple helix of native collagens (CD studies). The stability of spatial structures formed as a result of self-organization at temperatures of 4 °C, 20 °C and 40 °C was found. The high stability of the created supra-structures at elevated temperatures allows us to conclude that they are useful in regenerative medicine. Moreover, it was found that peptides **7**, **8**, **12**, **13**, **17**, **18**, **28**, **33**, **2.2**, **2.3** and **2.4** also form stable three-dimensional structures mimicking the triple helix of native collagen, but in this case, these structures were stable only at 4 °C.

The results showing the ability to create a variety of stable spatial structures, ranging from amyloid-type fibers to heliacal-type structures, have also been confirmed by microscopic examination. All persistent structures are obtained through self-organization of peptides derived from collagen IV.

All tested peptides are non-cytotoxic to BJ, BJ-5TA and C2C12 cell lines. Selected peptides also showed no genotoxicity and no induction of immune system responses.

The stability of the spatial structures of peptides **2**, **4**, **5**, **6**, **14**, **15**, **25**, **26** and **30** made it possible to obtain porous materials based on their equimolar mixture. The possibility of obtaining porous materials was found both as a result of cross-linking (formation of covalent bonds) and the material stabilized only by weak interactions. As a coupling reagent for cross-linking 4-(4,6-dimethoxy-1,3,5-triazin-2-yl)-4-methylmorpholinium toluene-4-sulfonate (DMT/NMM/TosO^−^) was used. Cross-linked porous solid materials are not cytotoxic to the BJ cell line (MTT test).

Research into the full characterization of porous structures and their use as scaffolds for tissue regeneration will be continued. However, a careful evaluation of the safety of collagen IV fragments that recreate the external sphere of the native protein, as well as their susceptibility to formation as a result of self-organization of stable spatial structures mimicking the structure of the native protein indicates that porous materials derived from collage IV peptides can be used in medicine regenerative.

## Figures and Tables

**Table 1:** Summary of purity of the peptides **1**–**33** (fragments selected in the first screening) and values for m/z confirming their structure.

Peptide	Fragment	Purity Based on HPLC	M Calculated [g/mol]	m/z Found
**1**	^21^AGEKSYGKPC^30^	88%	1038.47	529.2788 [M + 2H]^2+^ 1039.5492 [M + H]^+^
**2**	^41^CFPEKGARGR^50^	80%	1119.55	374.2167 [M + 3H]^3+^ 1120.6136 [M + H]^+^
**3**	^81^PGLLGPYGPK^90^	86%	997.55	499.8145 [M + 2H]^2+^ 998.6127 [M + H]^+^
**4**	^161^LAPGSFKGMK^170^	87%	1034.55	1035.6251 [M + H]^+^
**5**	^221^GFQGEKGVKG^230^	98%	1005.51	503.7991 [M + 2H]^2+^ 1006.5898 [M + H]^+^
**6**	^251^GFPKGKKGSK^260^	93%	1032.59	345.2301 [M + 3H]^3+^ 1033.6762 [M + H]^+^
**7**	^311^GPPGQQGKKG^320^	90%	952.49	953.5674 [M + H]^+^
**8**	^451^NKESGFPGLR^460^	95%	1103.56	552.8254 [M + 2H]^2+^
**9**	^471^LKGIKGDSGF^480^	85%	1020.55	511.2875 [M + 2H]^2+^ 1021.5612 [M + H]^+^
**10**	^511^KGARGDRGSG^520^	99%	959.47	320.8502 [M + 3H]^3+^ 480.7737 [M + 2H]^2+^ 960.5373 [M + H]^+^
**11**	^541^KGKKGEPILS^550^	85%	1055.62	528.8563 [M + 2H]^2+^ 1056.7046 [M + H]^+^
**12**	^641^PGQQGLPGSK^650^	99%	967.49	484.7949 [M + 2H]^2+^ 968.5737 [M + H]^+^
**13**	^671^PGFPGPKGSR^680^	87%	998.51	333.8715 [M + 3H]^3+^ 999.5955 [M + H]^+^
**14**	^711^GFPGPRGEKG^720^	84%	1000.49	501.2775 [M + 2H]^2+^ 1001.5502 [M + H]^+^
**15**	^721^LPGFPGLPGK^730^	91%	981.55	491.8182 [M + 2H]^2+^ 982.6217 [M + H]^+^
**16**	^781^PGLKGVHGKP^790^	97%	988.57	330.5547 [M + 3H]^3+^ 989.6496 [M + H]^+^
**17**	^821^GIKGKSGLPG^830^	99%	912.52	457.3051 [M + 2H]^2+^ 913.6027 [M + H]^+^
**18**	^841^PGKKGTRGKK^850^	81%	1055.64	352.9130 [M + 3H]^3+^ 1056.7249 [M + H]^+^
**19**	^851^GPPGSIVKKG^860^	95%	938.54	470.3106 [M + 2H]^2+^ 939.6105 [M + H]^+^
**20**	^891^LSGPKGEKGS^900^	82%	958,49	480,2892 [M + 2H]^2+^ 959,5691 [M + H]^+^
**21**	^921^LKGIPGSTGK^930^	91%	956.55	479.3194 [M + 2H]^2+^ 957.6297 [M + H]^+^
**22**	^951^PVGIPSPRRP^960^	92%	1074.62	359.2365 [M + 3H]^3+^ 538.3456 [M + 2H]^2+^ 1075.6860 [M + H]^+^
**23**	^961^MSNLWLKGDK^970^	91%	1190.60	397.9016 [M + 3H]^3+^ 596.3429 [M + 2H]^2+^ 1191.6721 [M + H]^+^
**24**	^1001^GAPGLPGIIK^1010^	80%	921.55	461.8178 [M + 2H]^2+^ 922.6209 [M + H]^+^
**25**	^1091^FKGTKGRDGL^1100^	92%	1077.58	360.2275 [M + 3H]^3+^ 1078.6536 [M + H]^+^
**26**	^1101^IGNIGFPGNK^1110^	80%	1015.53	508.8136 [M + 2H]^2+^ 1016.6166 [M + H]^+^
**27**	^1211^PGIGIGAPGK^1220^	95%	865.49	433.7870 [M + 2H]^2+^ 866.5585 [M + H]^+^
**28**	^1221^PGLRGQKGDR^1230^	80%	1082.58	361.8937 [M + 3H]^3+^ 1083.6613 [M + H]^+^
**29**	^1311^KGMRGEPGFM^1320^	92%	1108.51	555.3018 [M + 2H]^2+^ 1109.5893 [M + H]^+^
**30**	^1331^DPGFPGMKGK^1340^	92%	1032.49	517.2877 [M + 2H]^2+^ 1033.5643 [M + H]^+^
**31**	^1451^MRVGYTLVKH^1460^	80%	1202.65	401.8919 [M + 3H]^3+^ 1203.6398 [M + H]^+^
**32**	^1521^HYARRNDKSY^1530^	82%	1308.62	437.2168 [M + 3H]^3+^ 655.3173 [M + 2H]^2+^ 1309.6100 [M + H]^+^
**33**	^1661^AGQLHTRVSR^1670^	92%	1123.61	375.5688 [M + 3H]^3+^ 562.8491 [M + 2H]^2+^ 1124.6773 [M + H]^+^

**Table 3:** Summary of dependencies: the structure of collagen IV fragments, the ability to form structures that mimic the collagen helix, the dissociation constant and the maximum concentration. The green color indicates the amino acid residues present in the GFPGER sequence (ligand) with the highest affinity for α1β1 integrin and tested fragments. Red color—different amino acid residues.

Peptide	Fragment	Ability to Mimic the Structure of the Collagen Helix	K_d_ [μM]	Maximum Concentration
**4**	^161^LAPGSFKGMK^170^	+++	59.2 ± 12.5	2 mM
**5**	^221^GFQGEKGVKG^230^	+++	790.3 ± 174.2	4 mM
**8**	^451^NKESGFPGLR^460^	+	1465.3 ± 424.2	4 mM
**10**	^511^KGARGDRGSG^520^	-	47.3 ± 10.3	0.5 mM
**15**	^721^LPGFPGLPGK^730^	+++	no binding	2 mM
**19**	^851^GPPGSIVKKG^860^	-	2191.9 ± 741.9	4 mM
**25**	^1091^FKGTKGRDGL^1100^	+++	350.9 ± 109.9	1 mM
**30**	^1331^DPGFPGMKGK^1340^	+++	19.7 ± 5.6	2 mM

**Table 2:** Summary of purity of the peptides **2.1**–**2.4** (fragments selected in the second screening) and values for m/z confirming their structure.

Peptide	Fragment	Purity Based on HPLC	M Calculated [g/mol]	m/z Found
**2.1**	^23^EKSYGKPCGGQDC^35^	99%	1371.52	1372.5602 [M + H]^+^
**2.2**	^1446^MPGQSMRVGYTL^1457^	99%	1339.60	1340.6140 [M + H]^+^
**2.3**	^1453^VGYTLVKHSQSE^1464^	99%	1347.49	1349.6571 [M + H]^+^
**2.4**	^1520^CHYARRNDKSYW^1531^	99%	1598.77	1600.6767 [M + H]^+^

All HPLC and MS spectra of the test peptides are shown in Supporting Materials (see Figures S1–S37: MS spectra of peptides **1**–**33** and **2.1**–**2.4**, Figures S38–S74: HPLC of peptides **1**–**33** and **2.1**–**2.4**.

## Supplementary Materials

The following are available online at https://www.mdpi.com/article/10.3390/ijms222413584/s1.
