# Supplementary material for: Screening of Self-Assembling of Collagen IV Fragments into Stable Structures Potentially Useful in Regenerative Medicine
Source: Int J Mol Sci. 2021 Dec 18;22(24):13584. doi: 10.3390/ijms222413584 (PMC8708666; doi:10.3390/ijms222413584)
Supplement: Supplementary file 1 [file ijms-22-13584-s001.zip › ijms-1469759-supplementary.pdf]

Screening of Self-Assembling of Collagen IV Fragments into Stable Structures Potentially Useful in Regenerative Medicine – Supplementary Materials

Marcin Kolasa [1], Grzegorz Galita [2], Ireneusz Majsterek [2], Ewa Kucharska [3], Katarzyna Czerczak [4], Joanna Wasko [4], Angelika Becht [4], Justyna Fraczyk [4], Anna Gajda [4], Lukasz Pietrzak [5], Lukasz Szymanski [5], Agnieszka Krakowiak [6], Zbigniew Draczynski [7] and Beata Kolesinska [4,*]

[1] General Command of the Polish Armed Forces, Medical Division, Zwirki i Wigury 103/105, 00-912 Warsaw, Poland; makolasa@ron.mil.pl
[2] Department of Clinical Chemistry and Biochemistry, Medical University of Lodz, Narutowicza 60, 90-136 Lodz, Poland; grzegorz.galita@umed.lodz.pl (G.G.); ireneusz.majsterek@umed.lodz.pl (I.M.)
[3] Department Geriatrics and Social Work, Jesuit University Ignatianum in Cracow, Kopernika 26, 31-501 Krakow, Poland; ewa.kucharska@ignatianum.edu.pl
[4] Institute of Organic Chemistry, Faculty of Chemistry, Lodz University of Technology, Zeromskiego 116, 90-924 Lodz, Poland; katarzyna.czerczak@dokt.p.lodz.pl (K.C.); joanna.wasko@p.lodz.pl (J.W.); anglika.becht@dokt.p.lodz.pl (A.B.); justyna.fraczyk@p.lodz.pl (J.F.); anna.gajda@p.lodz.pl (A.G.)
[5] Institute of Mechatronics and Information Systems, Faculty of Electrical, Electronic, Computer and Control Engineering, Lodz University of Technology, Stefanowskiego 18/22, 90-924 Lodz, Poland; lukasz.pietrzak@p.lodz.pl (L.P.); lukasz.szymanski@p.lodz.pl (L.S.)
[6] Centre of Molecular and Macromolecular Studies of the Polish Academy of Sciences, Department of Bioorganic Chemistry, Sienkiewicza 112, 90-363 Lodz, Poland; akrakow@cbmm.lodz.pl
[7] Institute of Material Sciences of Textiles and Polymer Composites, Faculty of Material Technologies and Textile Design, Lodz University of Technology, Zeromskiego 116, 90-924 Lodz, Poland; zbigniew.draczynski@p.lodz.pl
* Correspondence: beata.kolesinska@p.lodz.pl; Tel.: 48-42-631-32-61

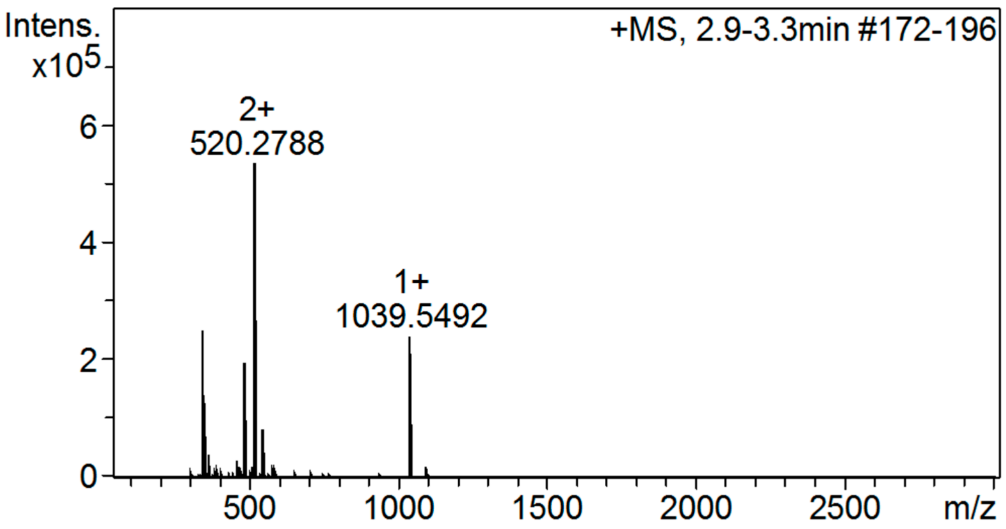

Figure S1. MS spectrum of peptide **1**.

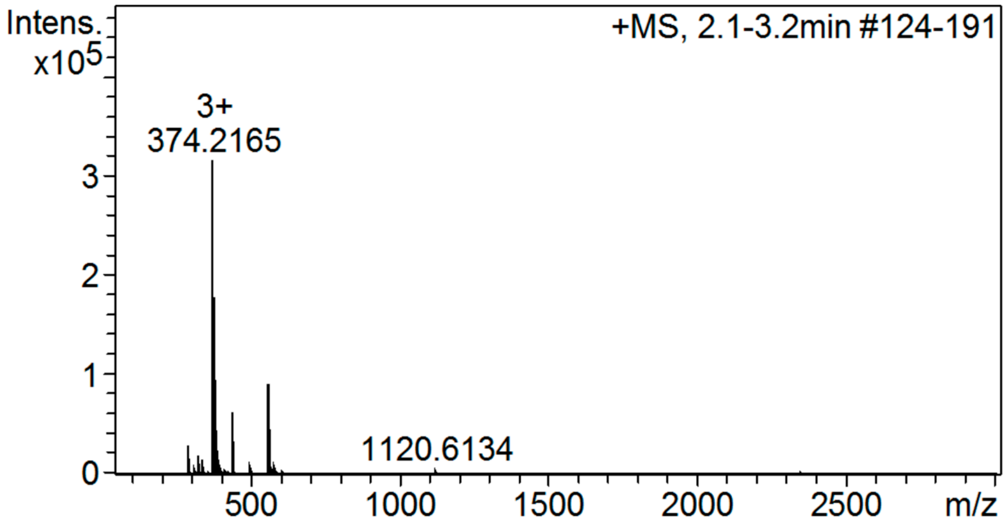

Figure S2. MS spectrum of peptide **2**.

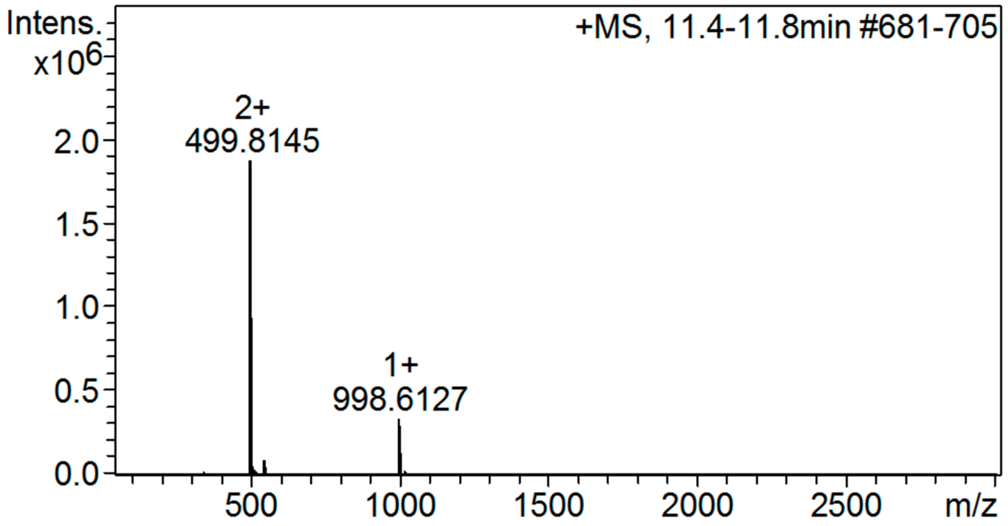

Figure S3. MS spectrum of peptide **3**.

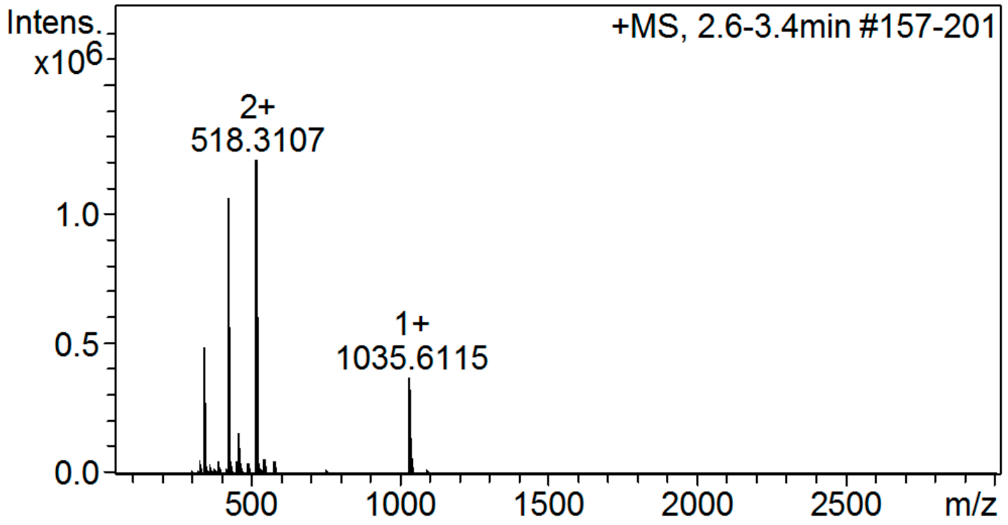

Figure S4. MS spectrum of peptide **4**.

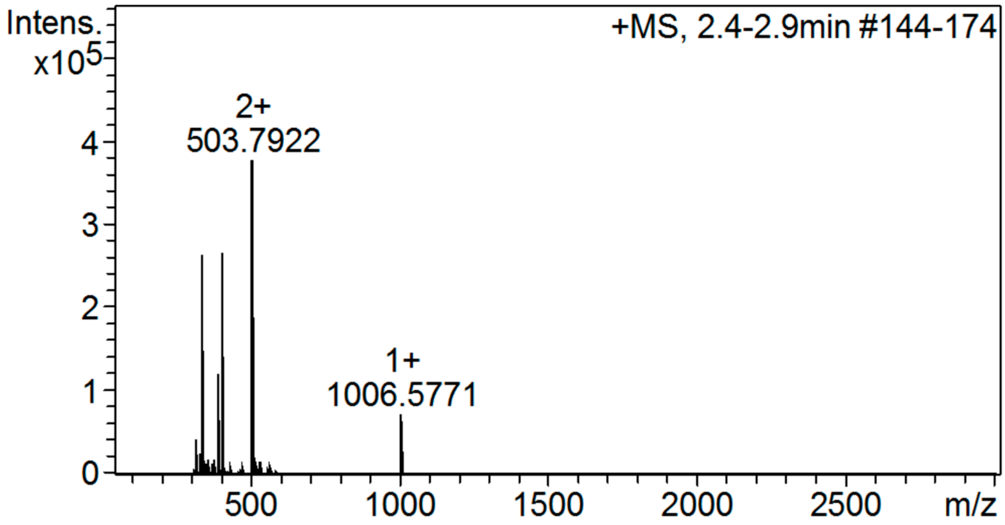

Figure S5. MS spectrum of peptide **5**.

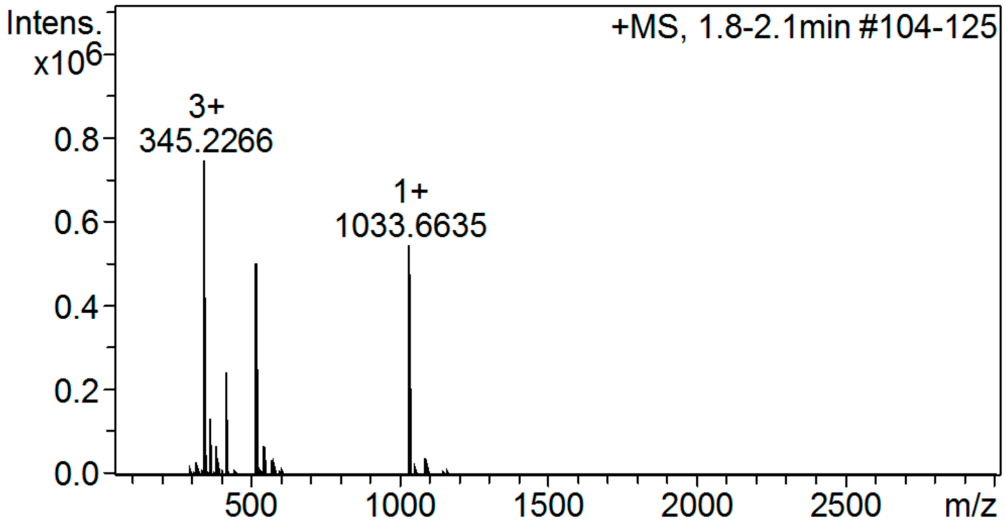

Figure S6. MS spectrum of peptide **6**.

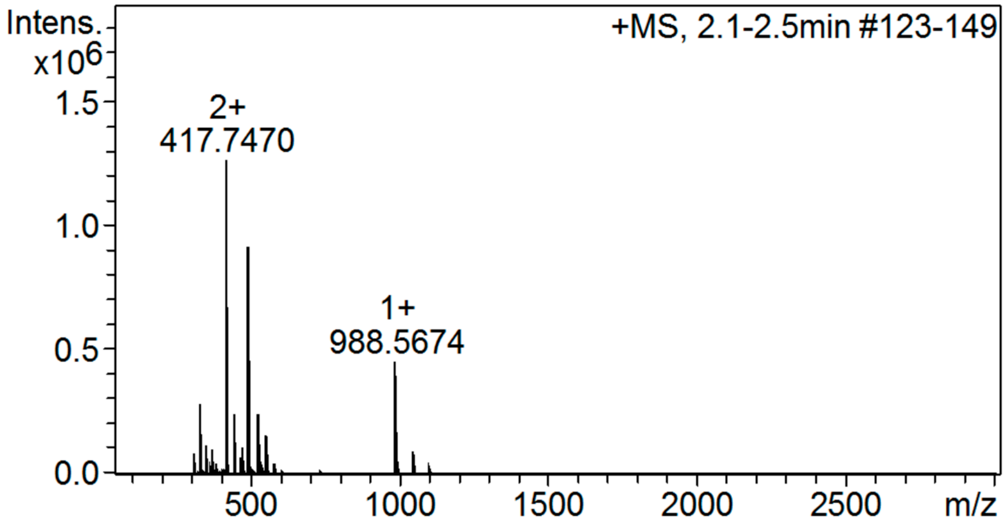

Figure S7. MS spectrum of peptide **7**.

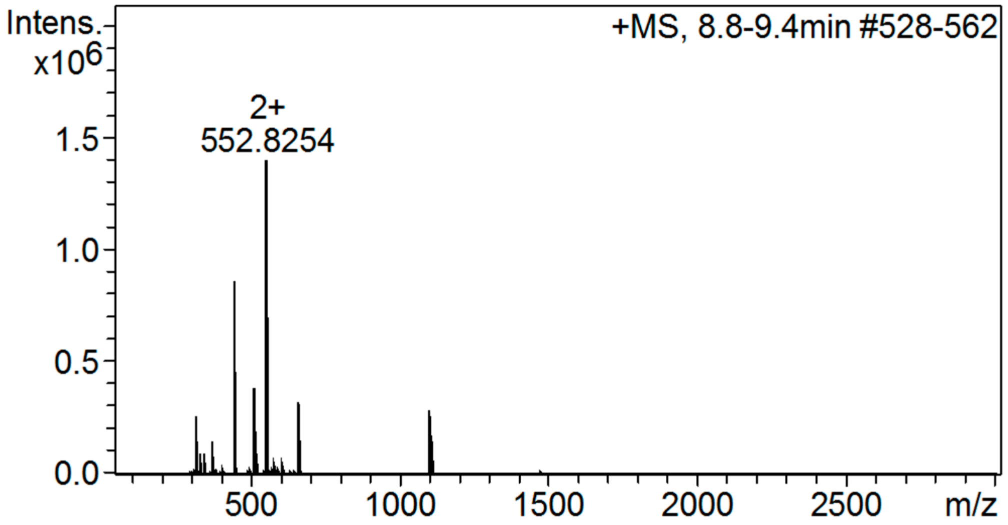

Figure S8. MS spectrum of peptide **8**.

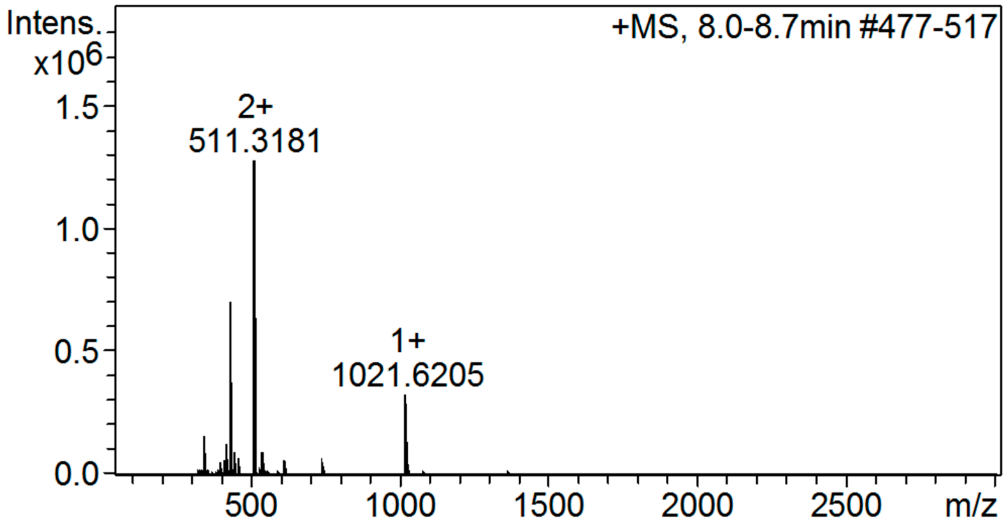

Figure S9. MS spectrum of peptide **9**.

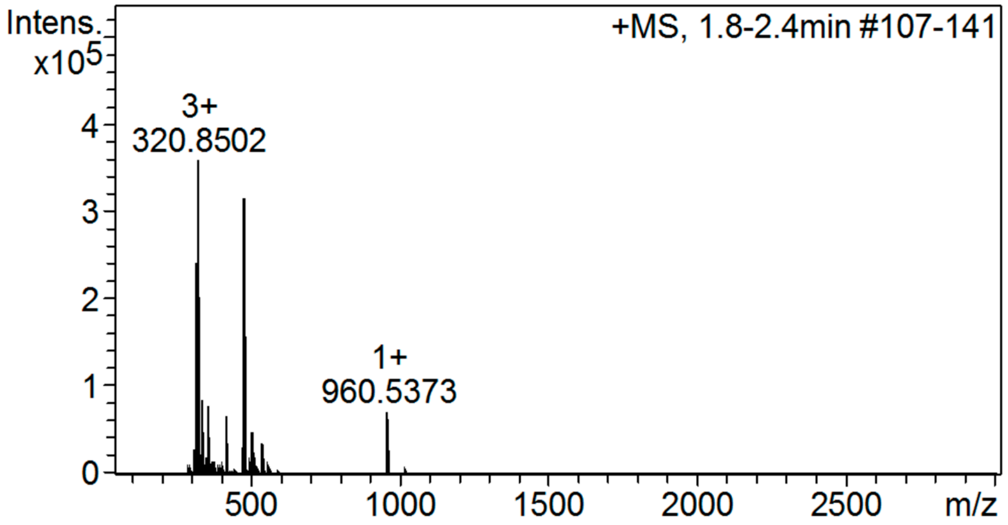

Figure S10. MS spectrum of peptide **10**.

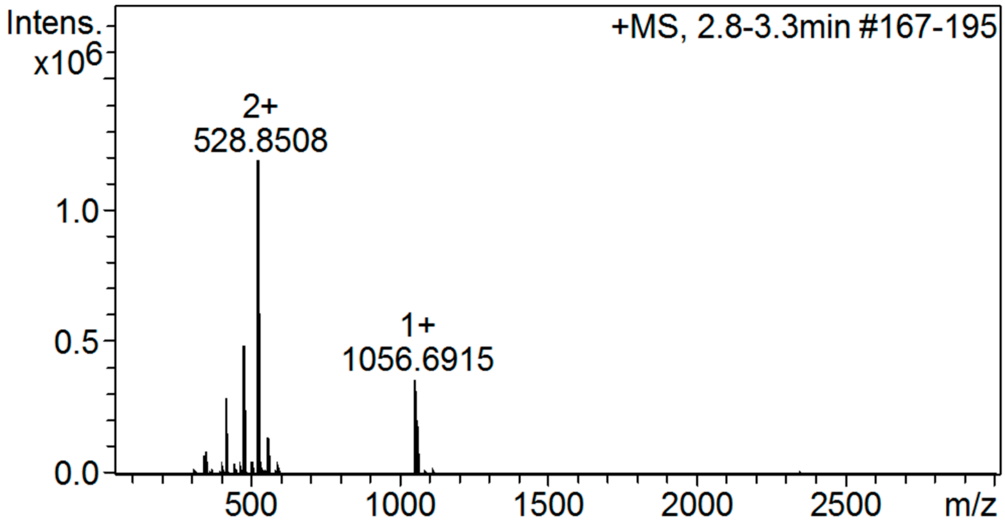

Figure S11. MS spectrum of peptide **11**.

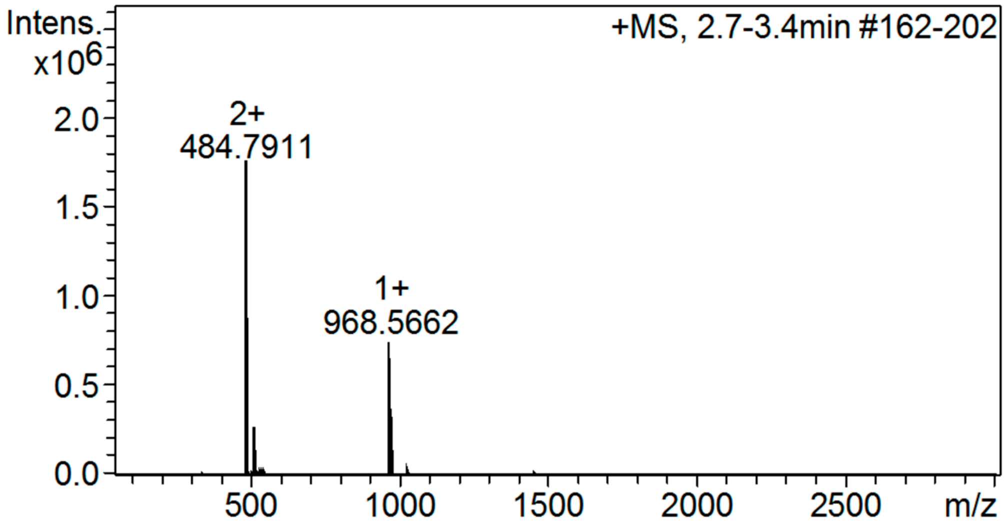

Figure S12. MS spectrum of peptide **12**.

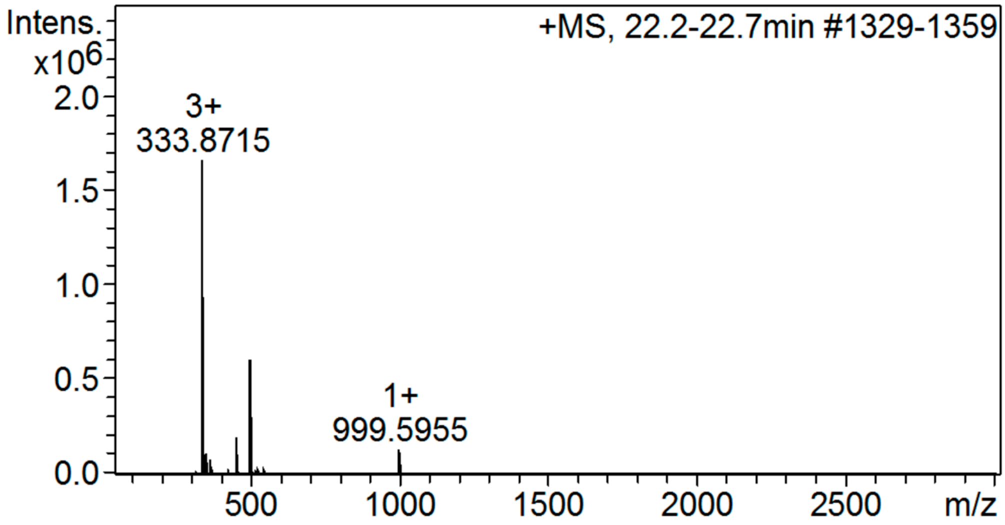

Figure S13. MS spectrum of peptide **13**.

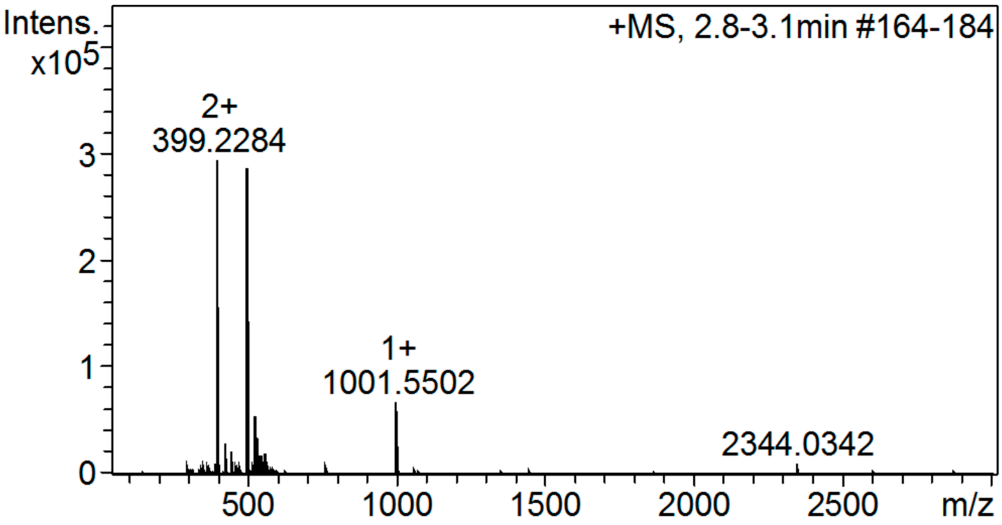

Figure S14. MS spectrum of peptide **14**.

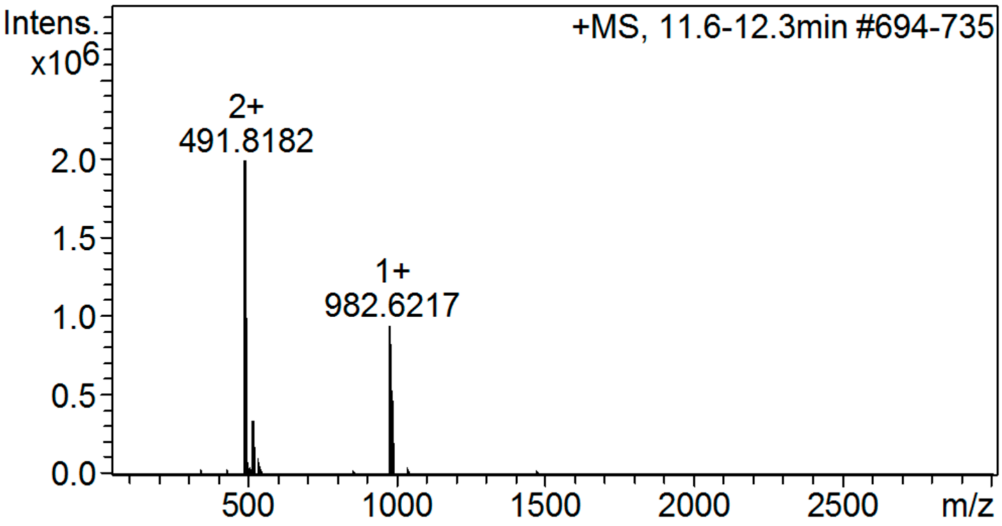

Figure S15. MS spectrum of peptide **15**.

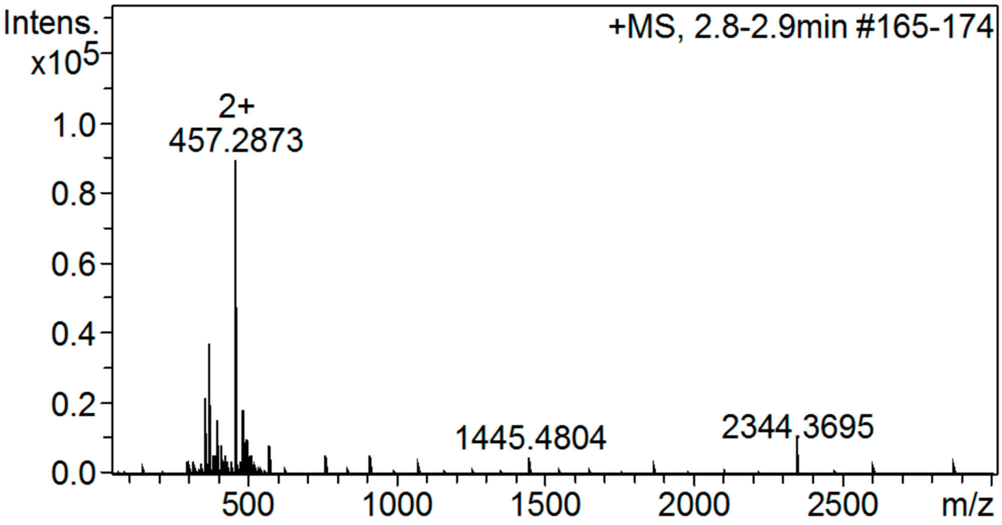

Figure S16. MS spectrum of peptide **16**.

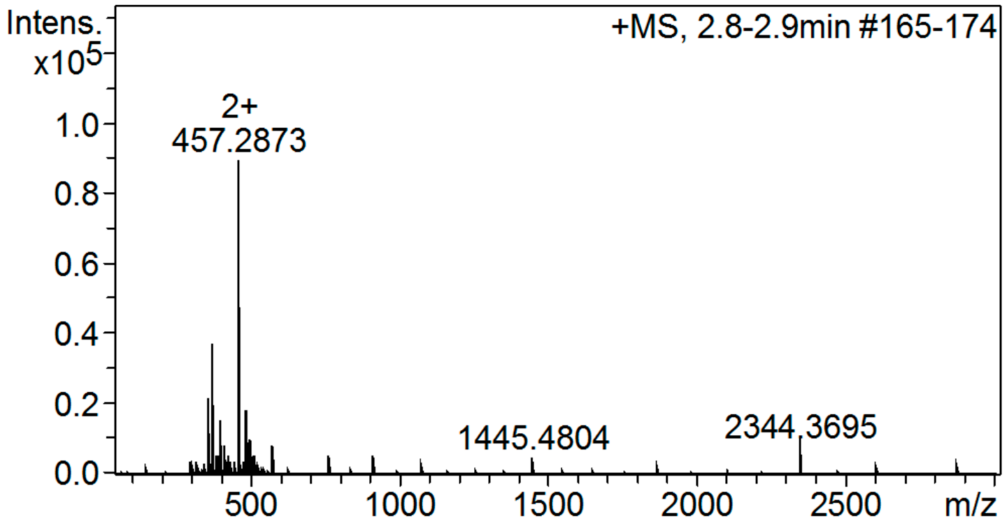

Figure S17. MS spectrum of peptide **17**.

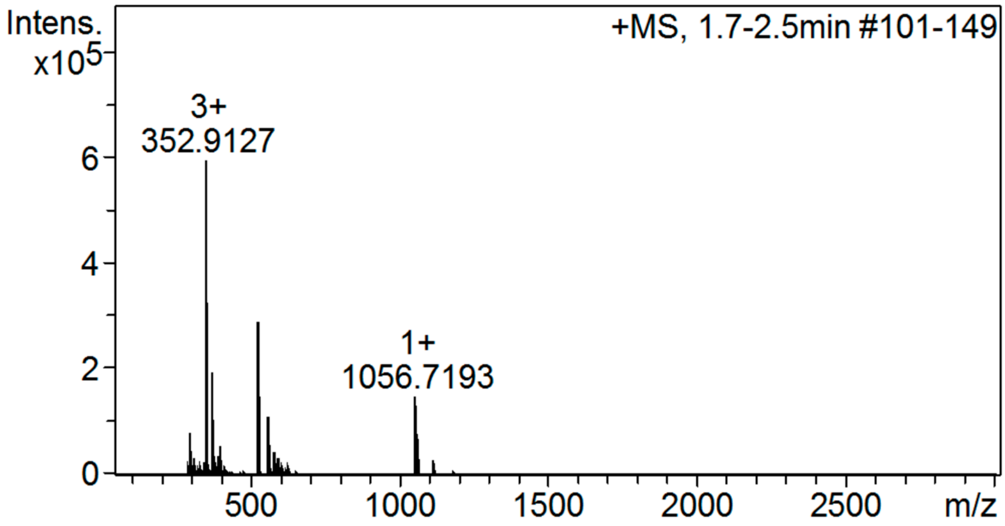

Figure S18. MS spectrum of peptide **18**.

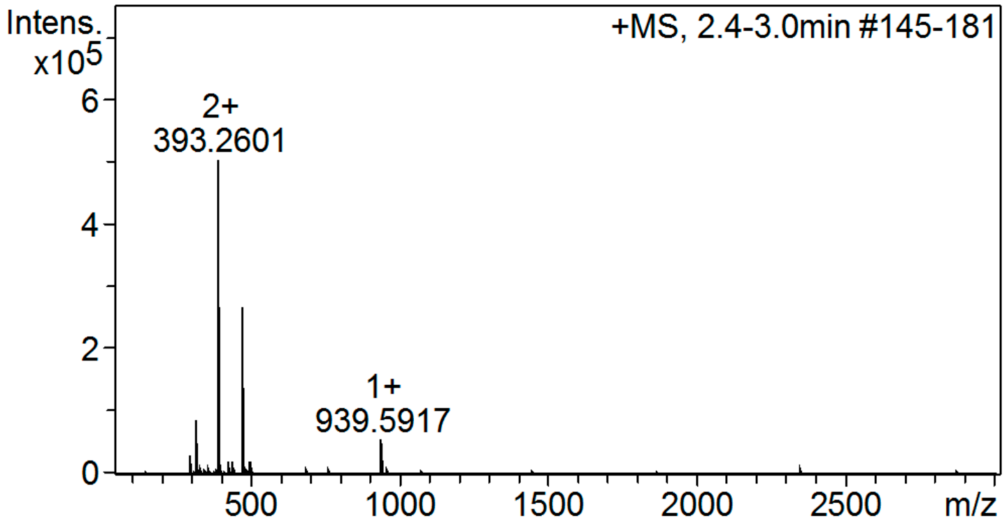

Figure S19. MS spectrum of peptide **19**.

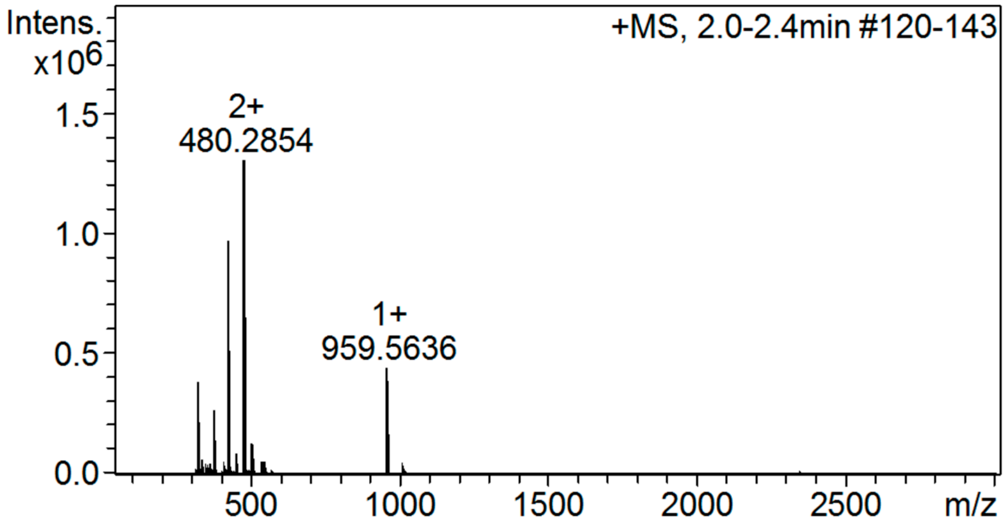

Figure S20. MS spectrum of peptide **20**.

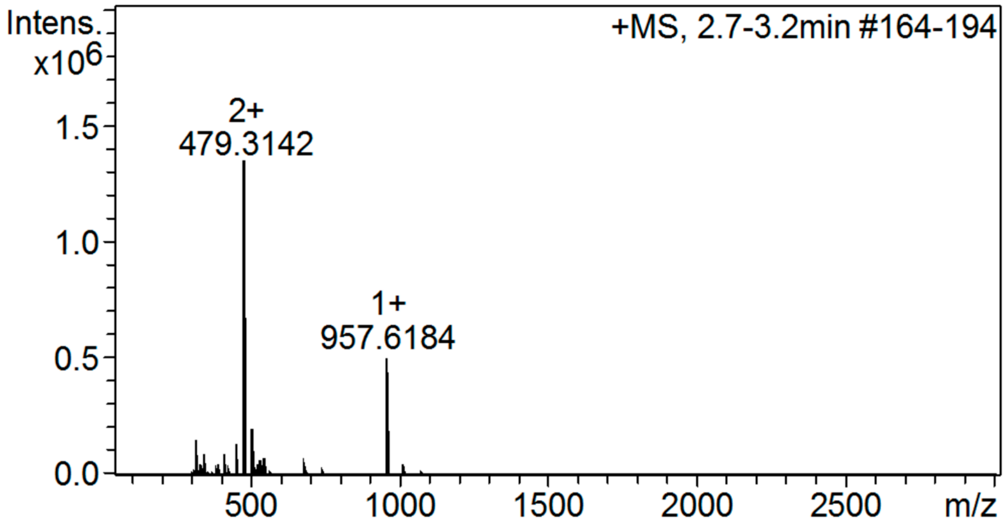

Figure S21. MS spectrum of peptide **21**.

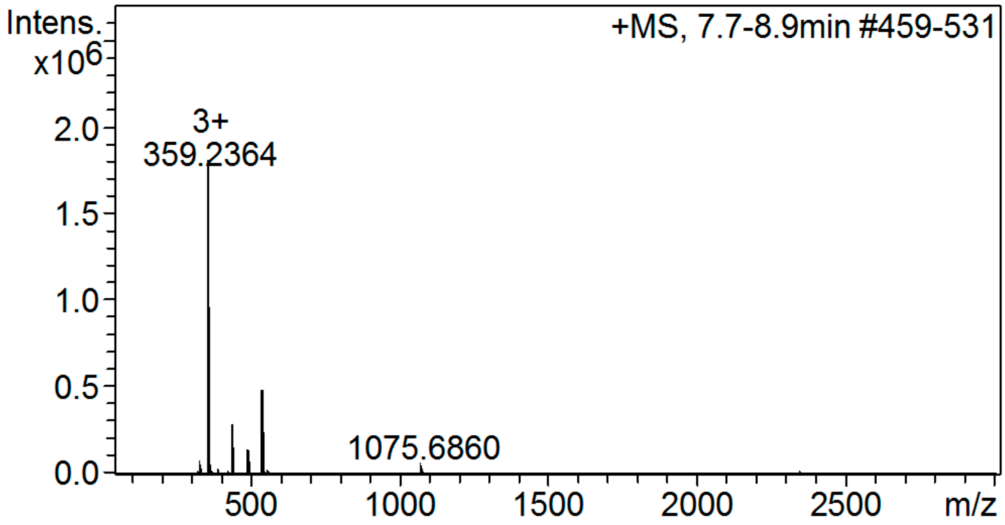

Figure S22. MS spectrum of peptide **22**.

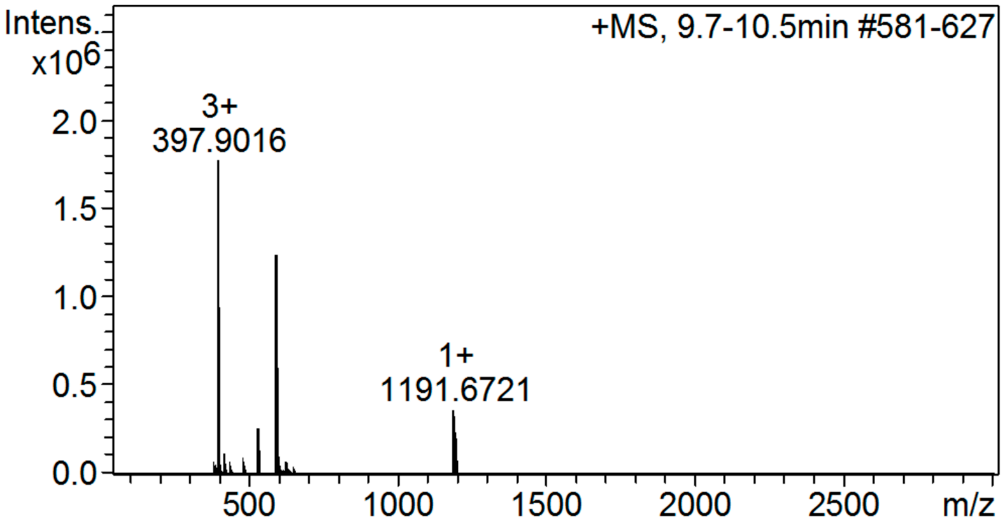

Figure S23. MS spectrum of peptide **23**.

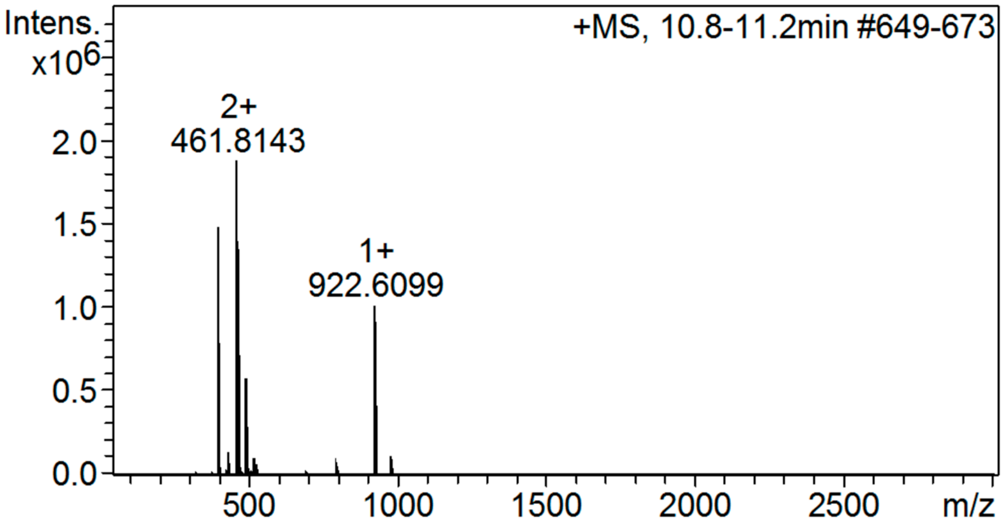

Figure S24. MS spectrum of peptide **24**.

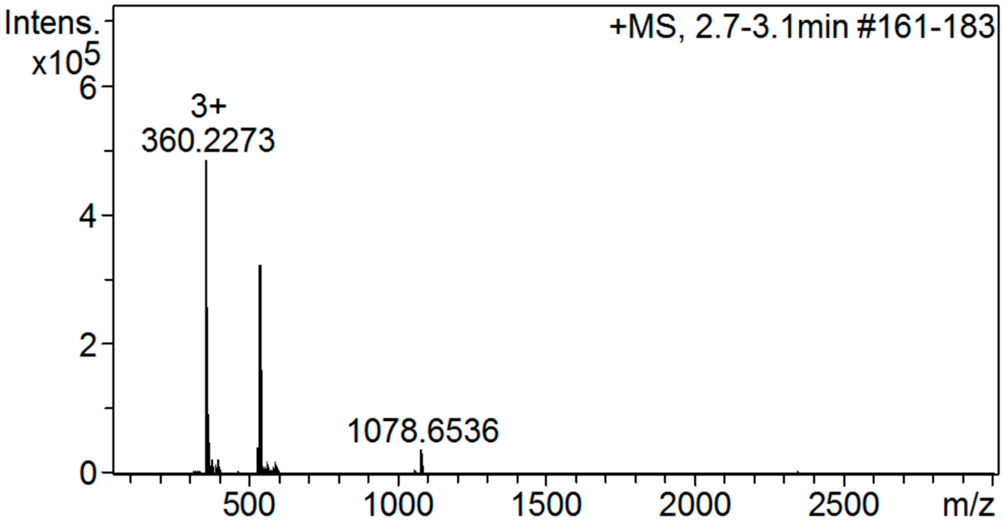

Figure S25. MS spectrum of peptide **25**.

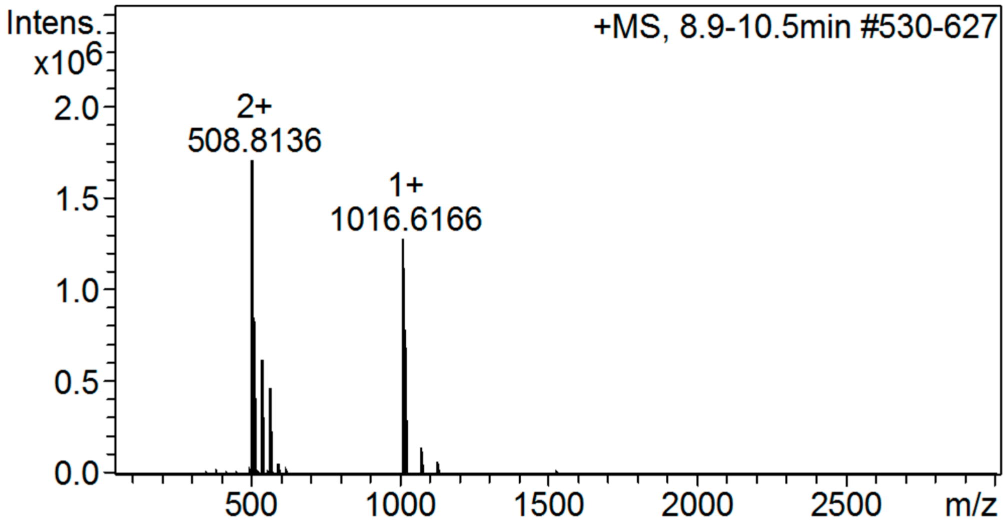

Figure S26. MS spectrum of peptide **26**.

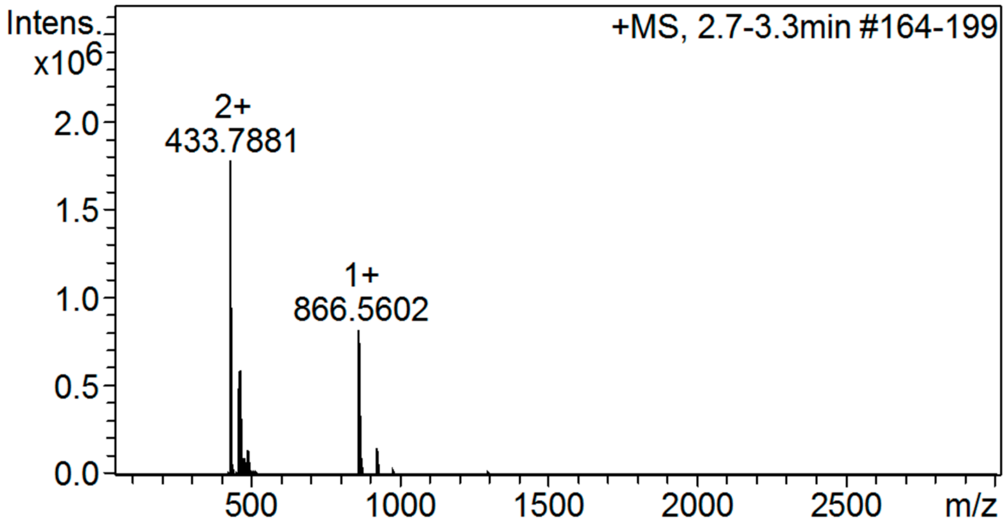

Figure S27. MS spectrum of peptide **27**.

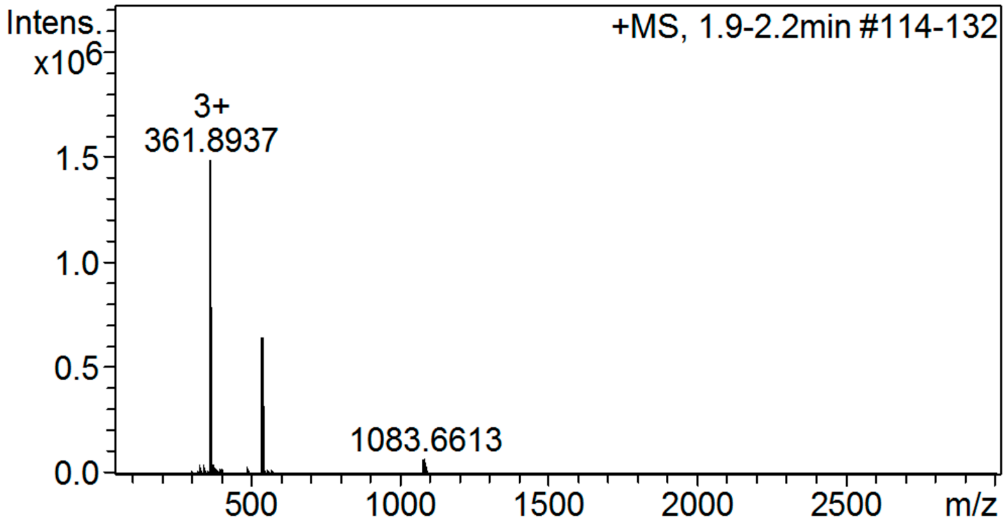

Figure S28. MS spectrum of peptide **28**.

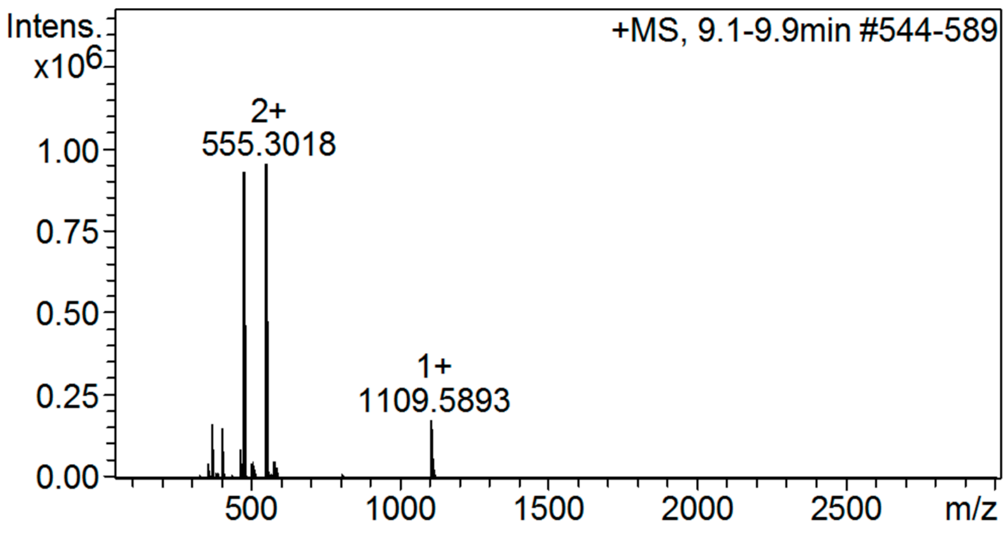

Figure S29. MS spectrum of peptide **29**.

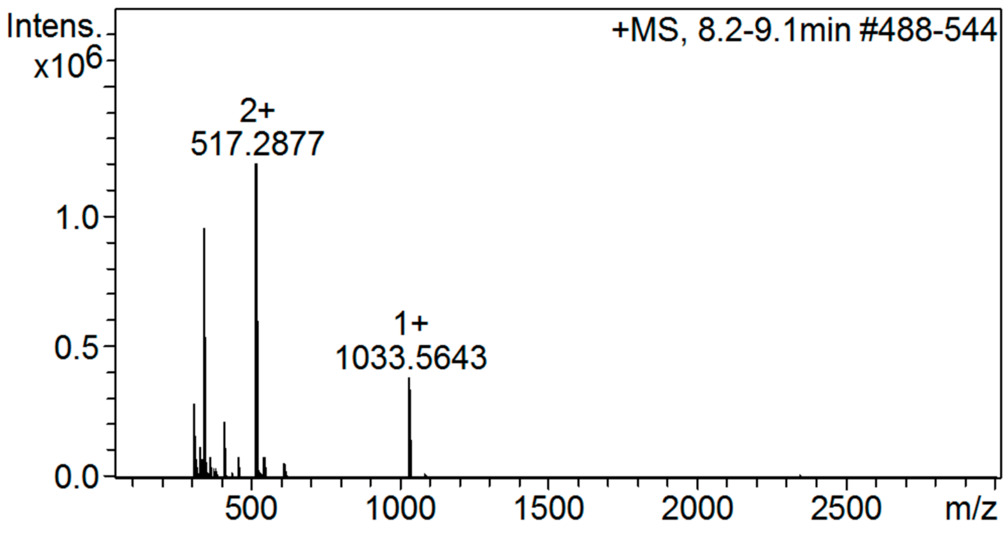

Figure S30. MS spectrum of peptide **30**.

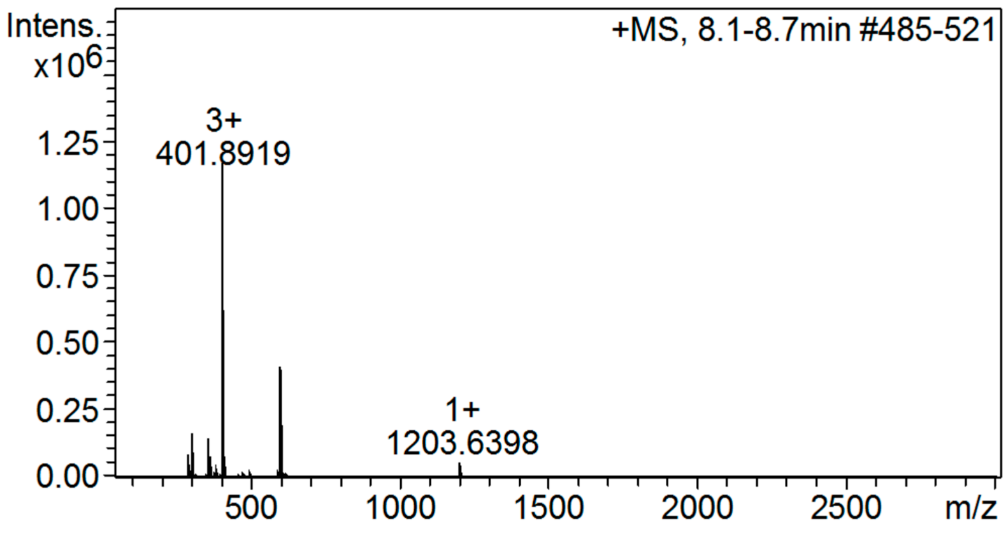

Figure S31. MS spectrum of peptide **31**.

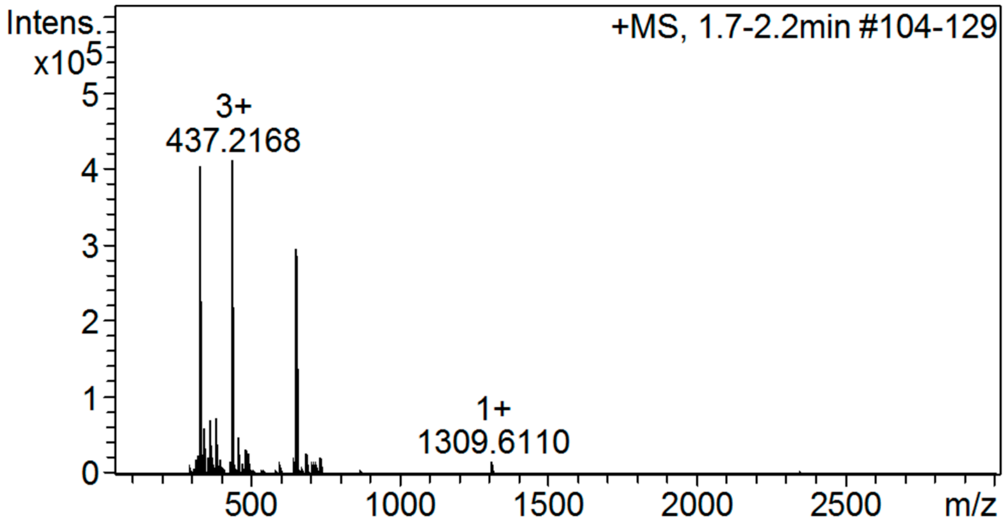

Figure S32. MS spectrum of peptide **32**.

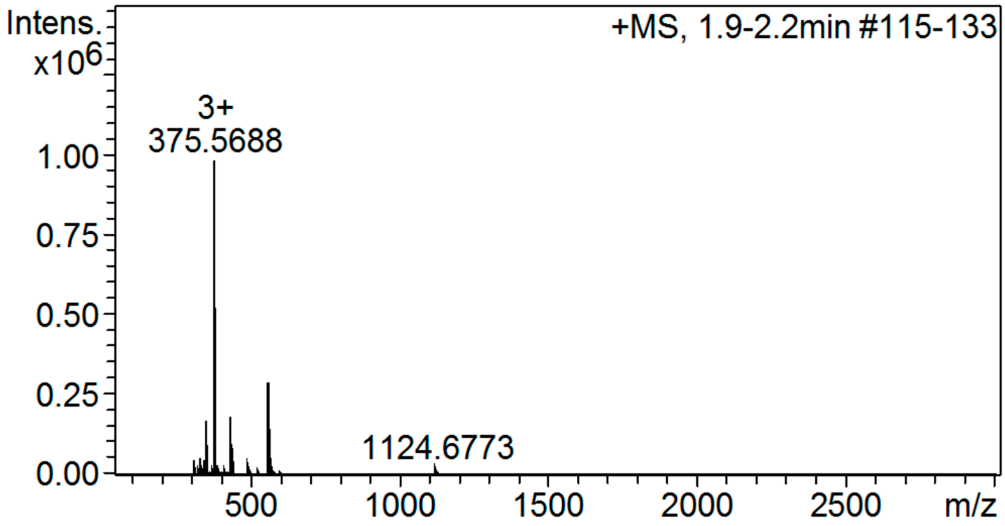

Figure S33. MS spectrum of peptide **33**.

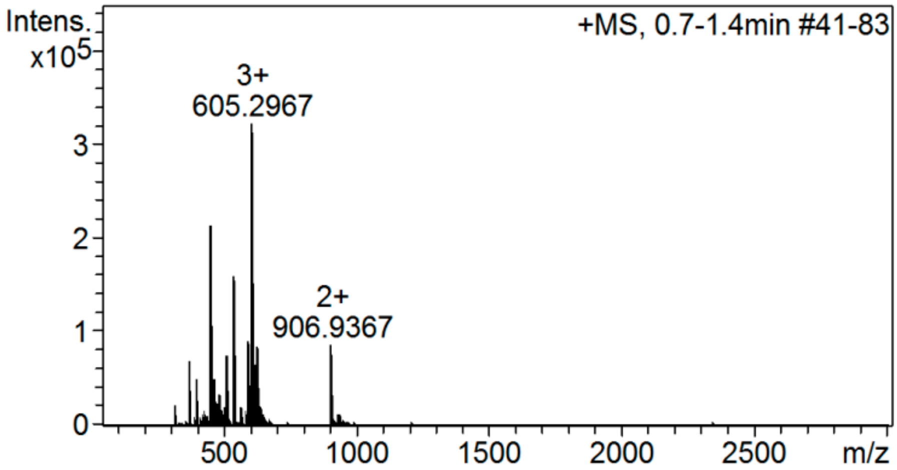

Figure S34. MS spectrum of peptide **2.1**.

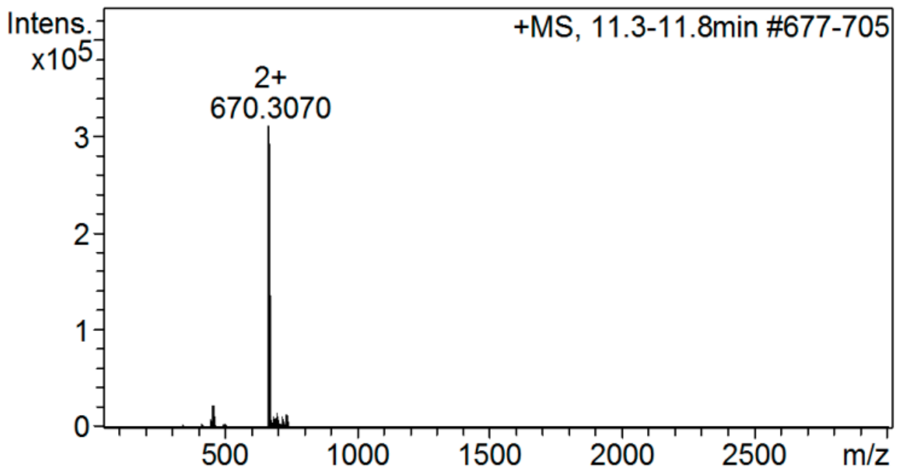

Figure S35. MS spectrum of peptide **2.2**.

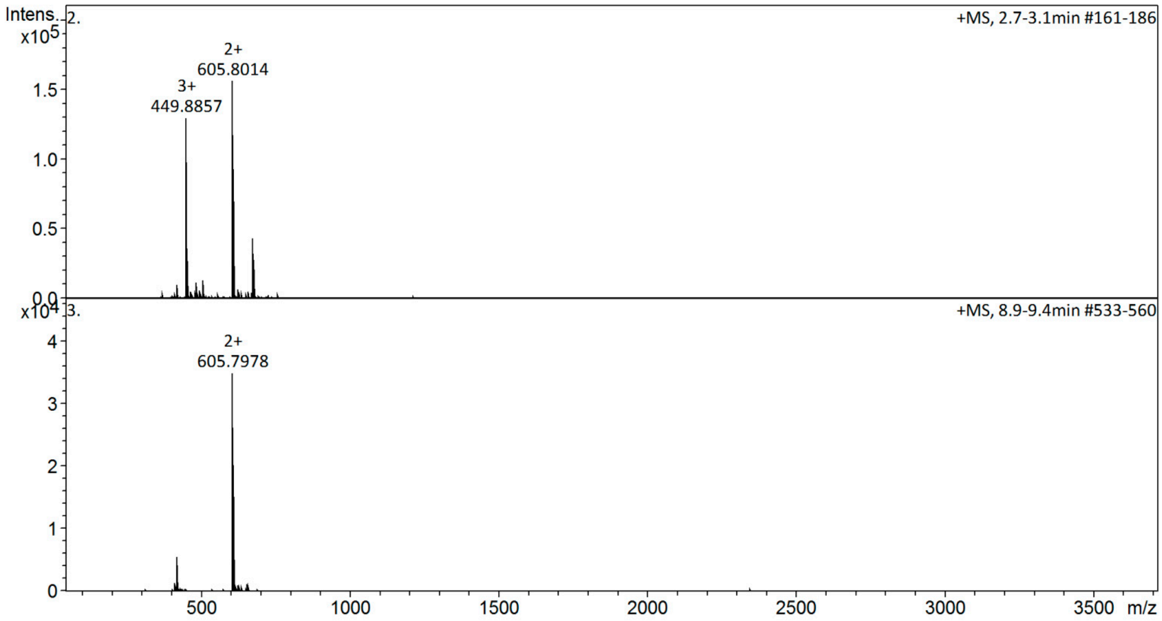

Figure S36. MS spectrum of peptide **2.3**.

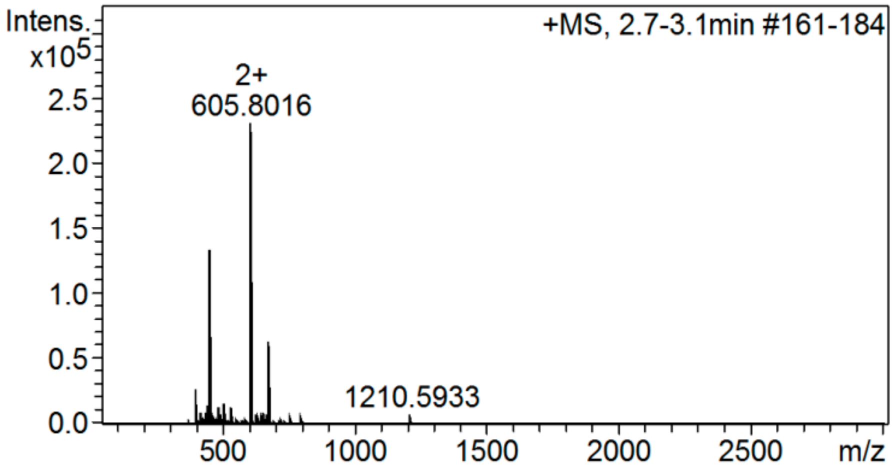

Figure S37. MS spectrum of peptide **2.4**.

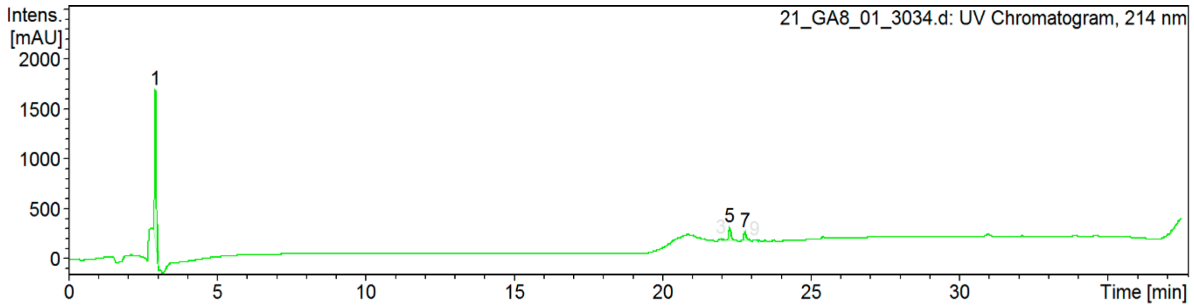

Figure S38. HPLC of peptide **1**.

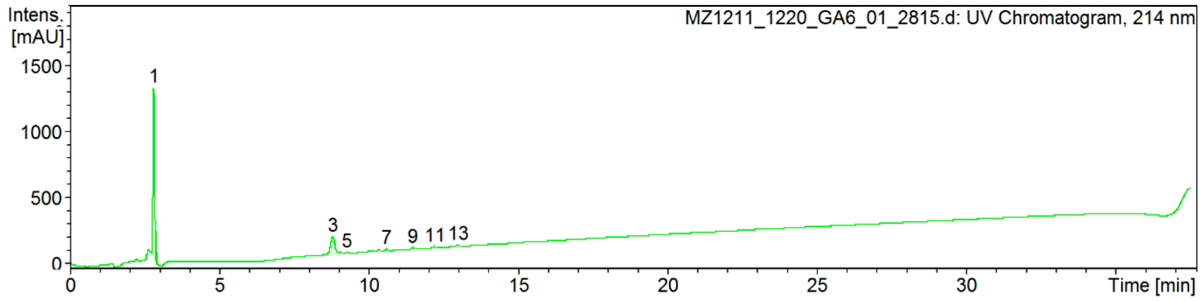

Figure S39. HPLC of peptide **2**.

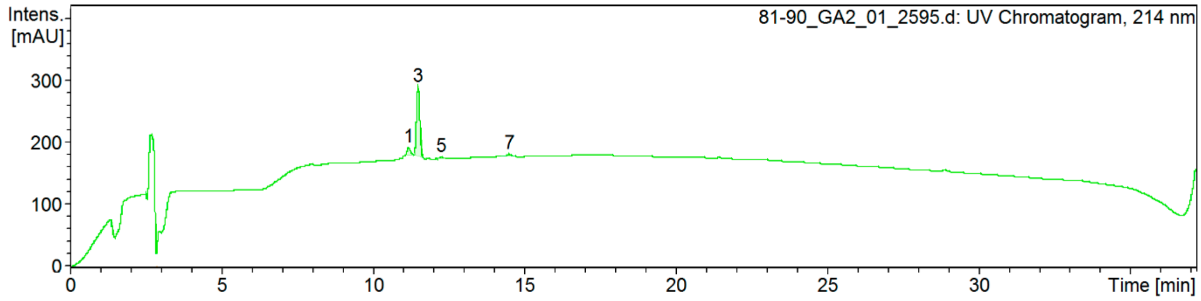

Figure S40. HPLC of peptide **3**.

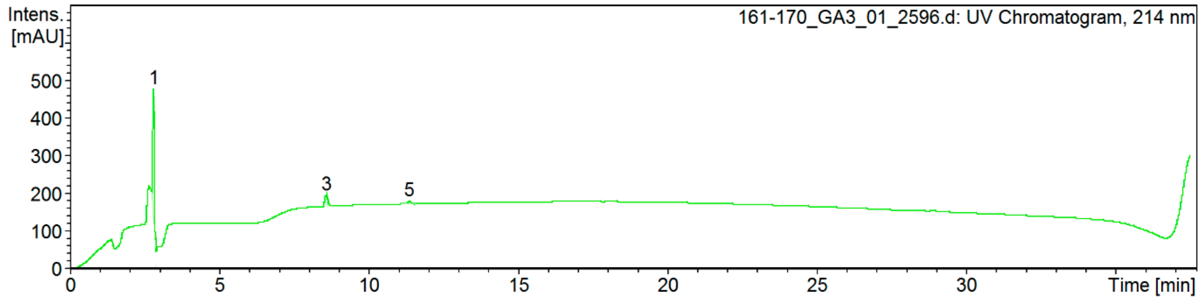

Figure S41. HPLC of peptide **4**.

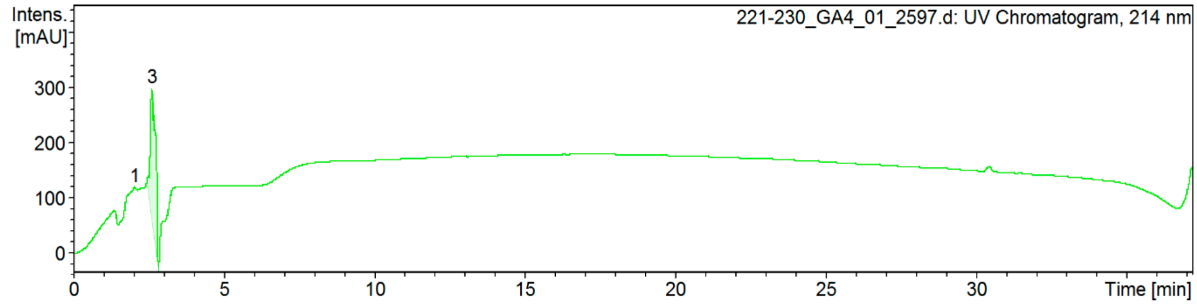

Figure S42. HPLC of peptide **5**.

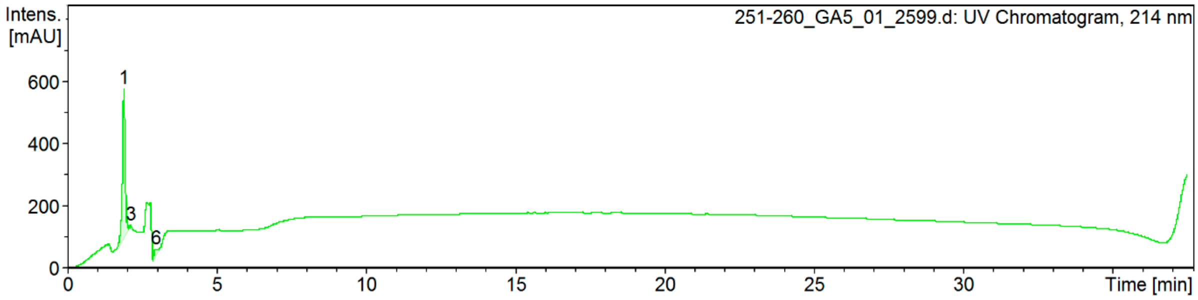

Figure S43. HPLC of peptide **6**.

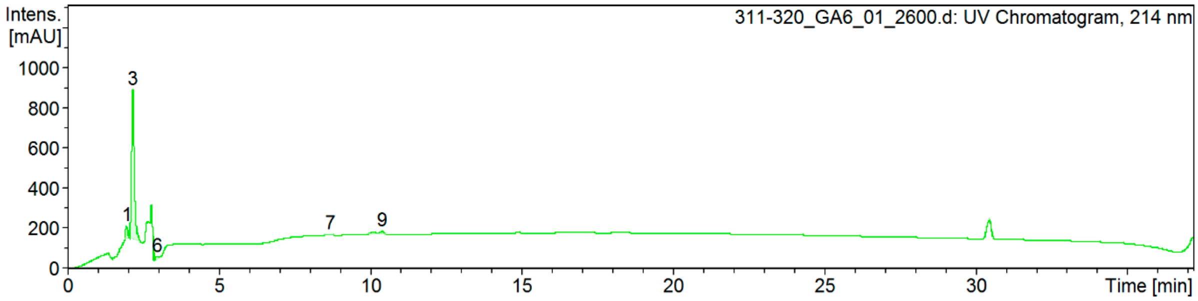

Figure S44. HPLC of peptide **7**.

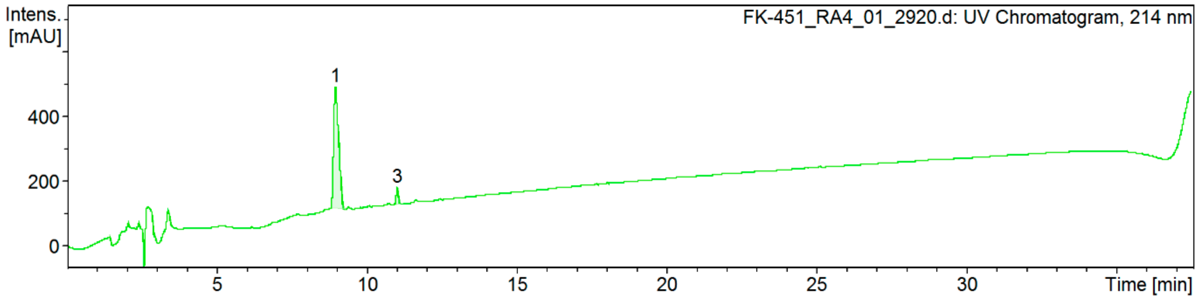

Figure S45. HPLC of peptide **8**.

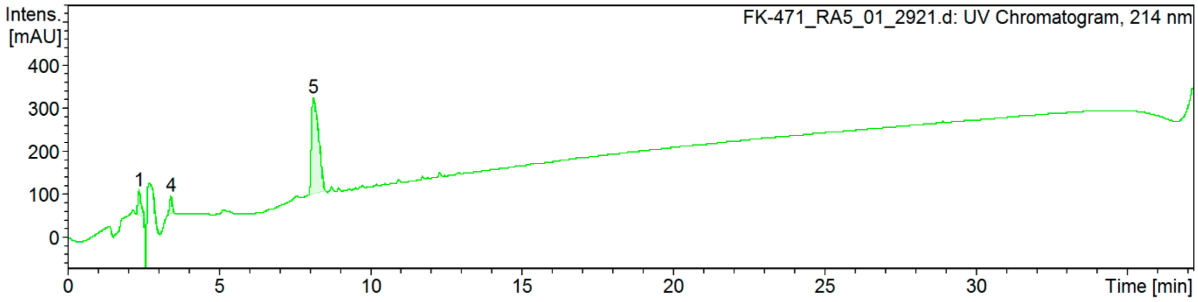

Figure S46. HPLC of peptide **9**.

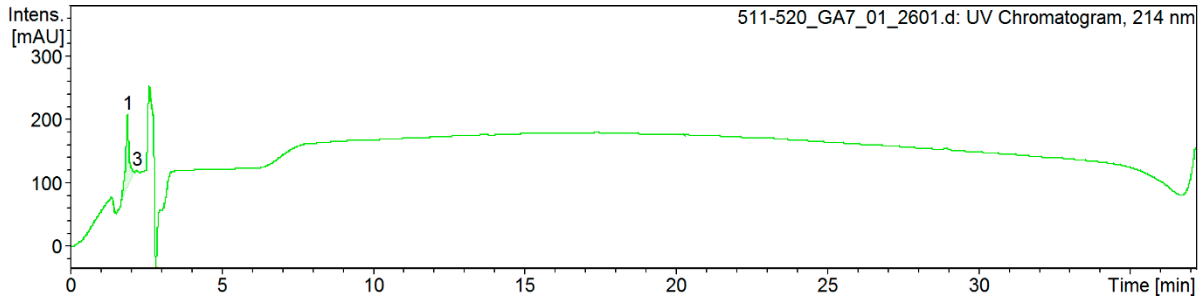

Figure S47. HPLC of peptide **10**.

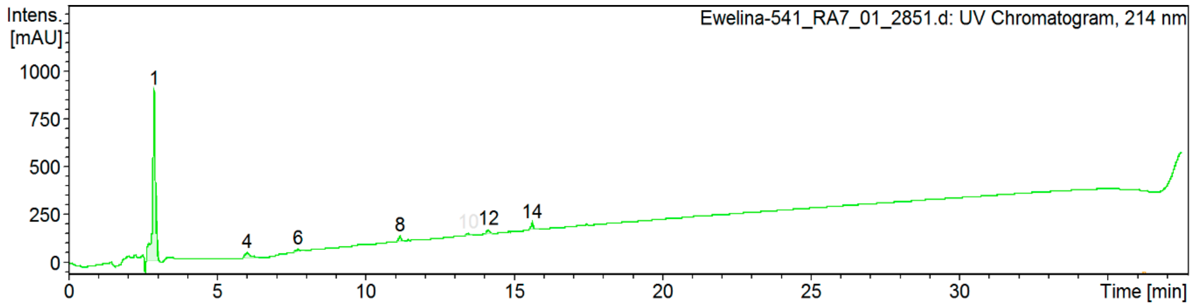

Figure S48. HPLC of peptide **11**.

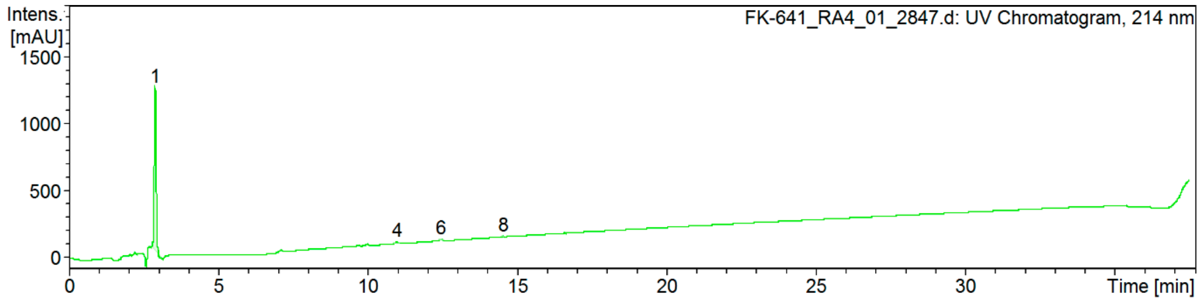

Figure S49. HPLC of peptide **12**.

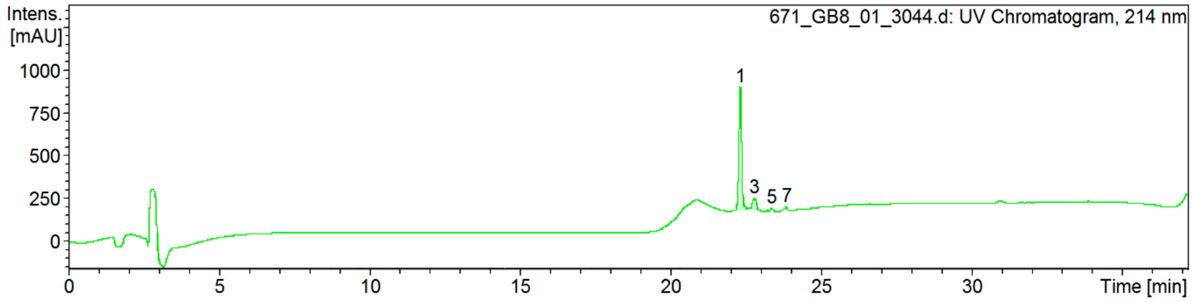

Figure S50. HPLC of peptide **13**.

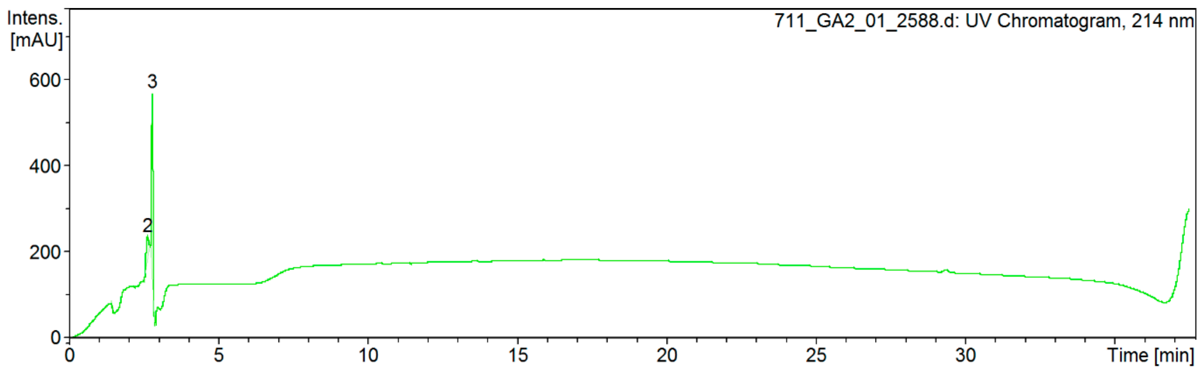

Figure S51. HPLC of peptide **14**.

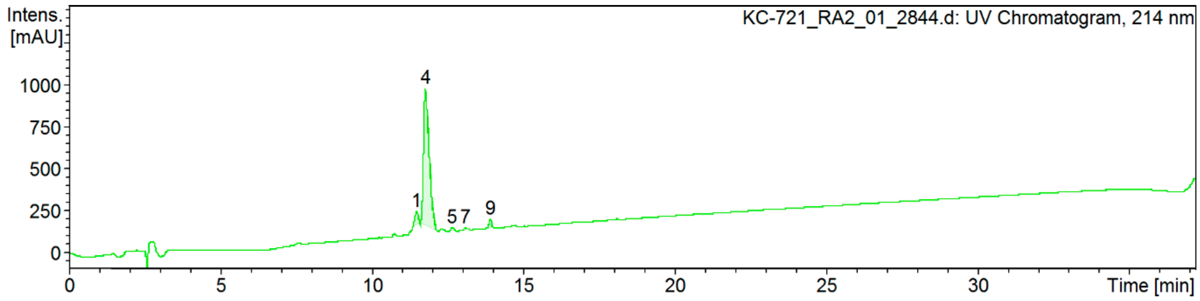

Figure S52. HPLC of peptide **15**.

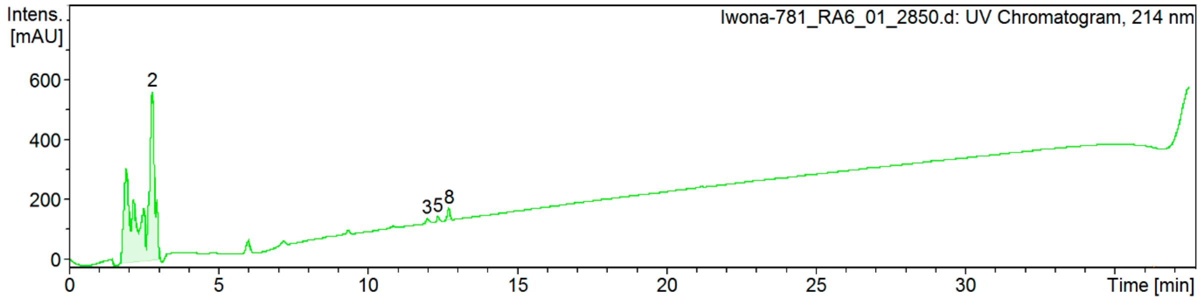

Figure S53. HPLC of peptide **16**.

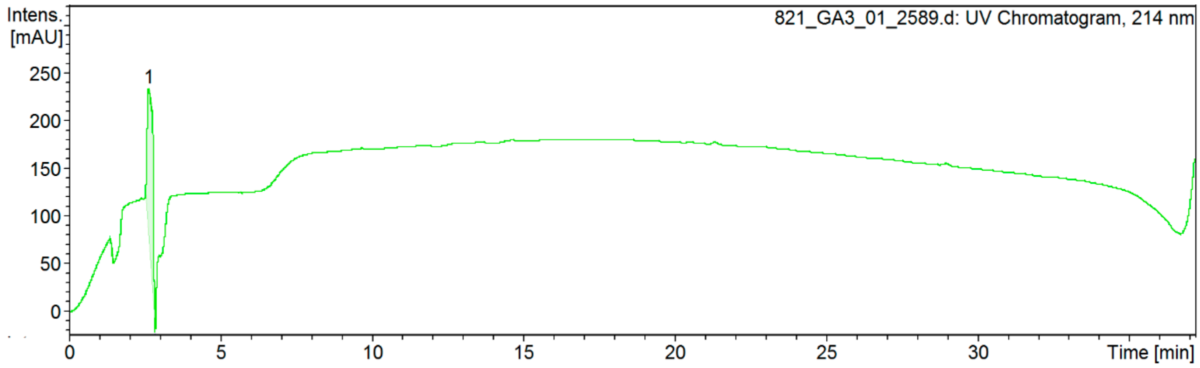

Figure S54. HPLC of peptide **17**.

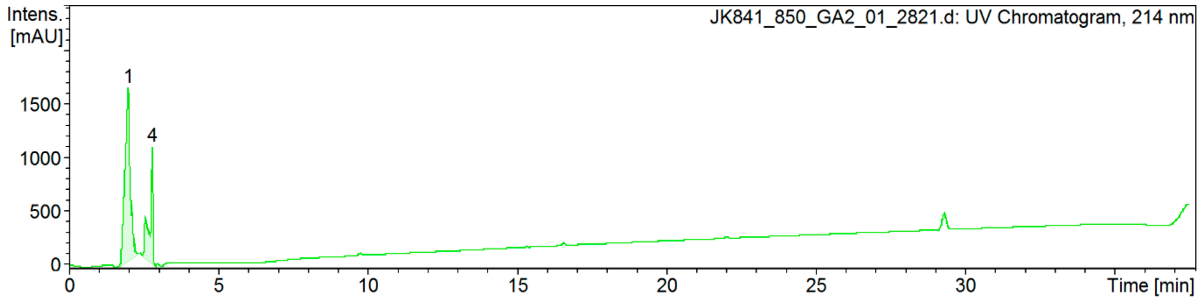

Figure S55. HPLC of peptide **18**.

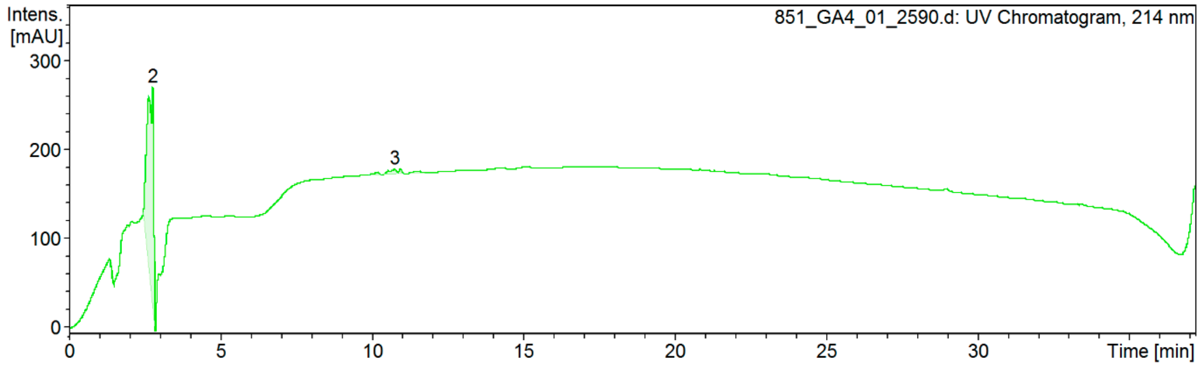

Figure S56. HPLC of peptide **19**.

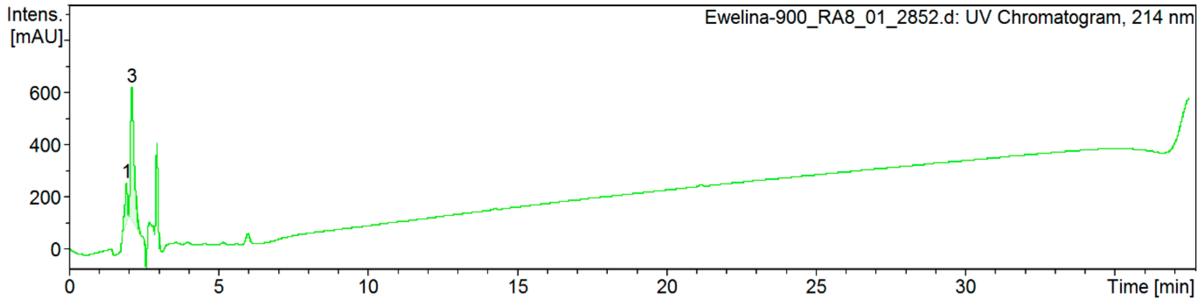

Figure S57. HPLC of peptide **20**.

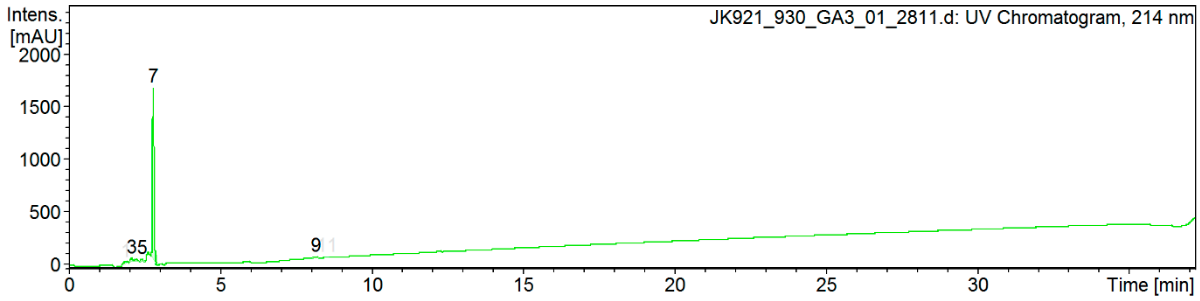

Figure S58. HPLC of peptide **21**.

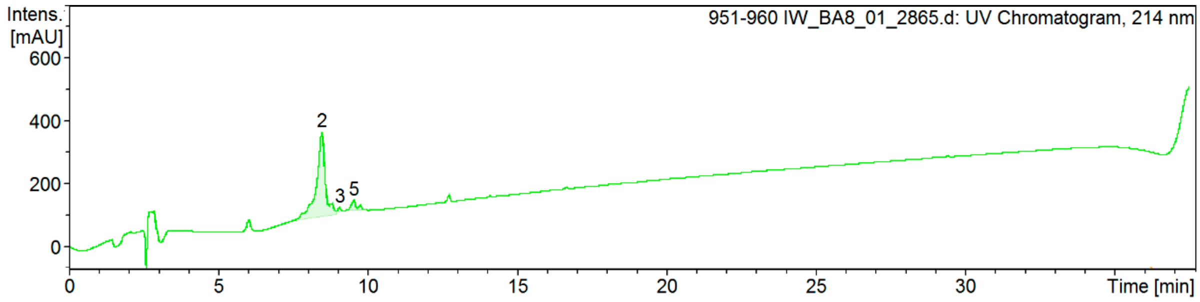

Figure S59. HPLC of peptide **22**.

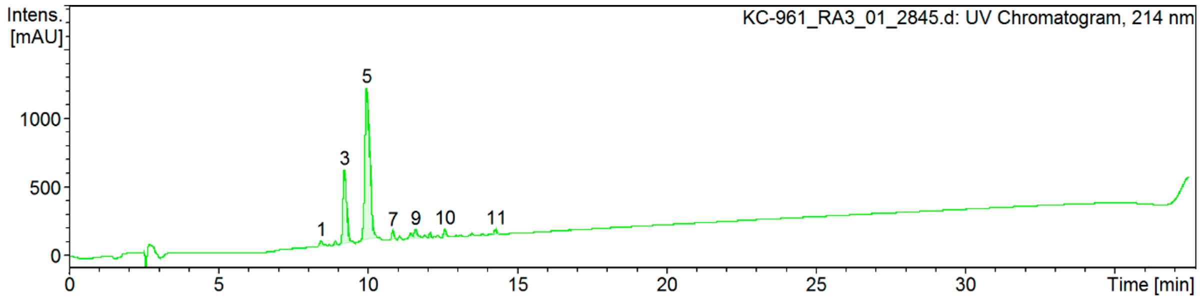

Figure S60. HPLC of peptide **23**.

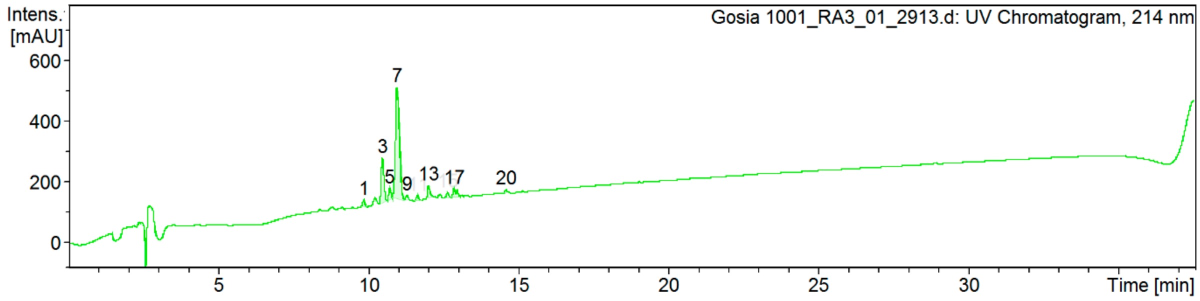

Figure S61. HPLC of peptide **24**.

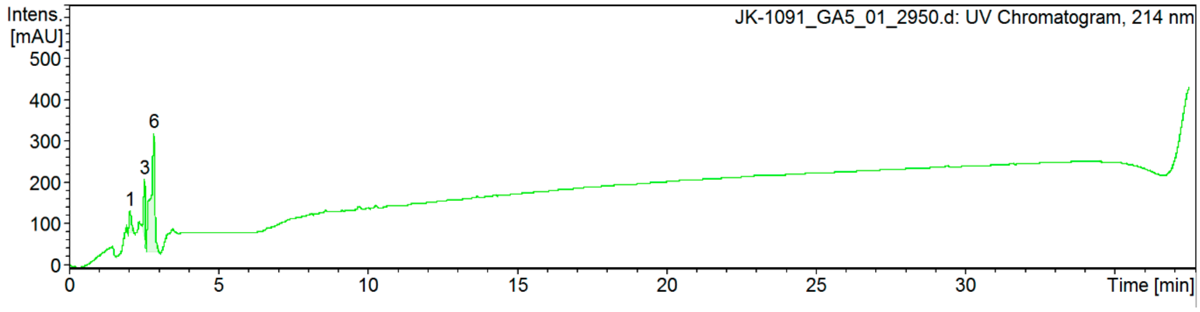

Figure S62. HPLC of peptide **25**.

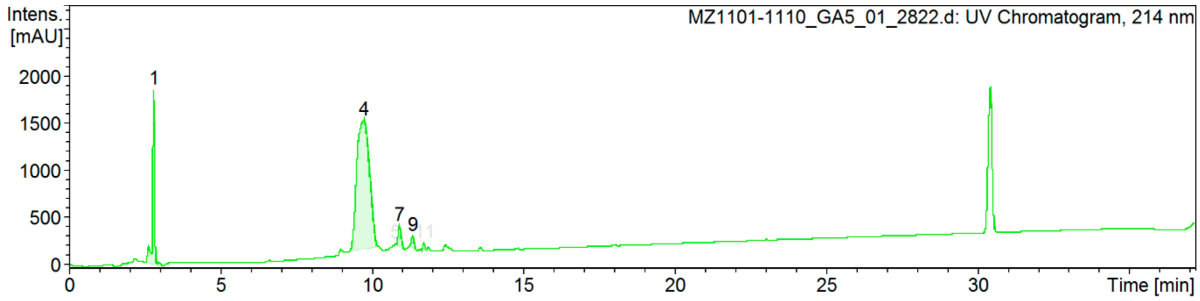

Figure S63. HPLC of peptide **26**.

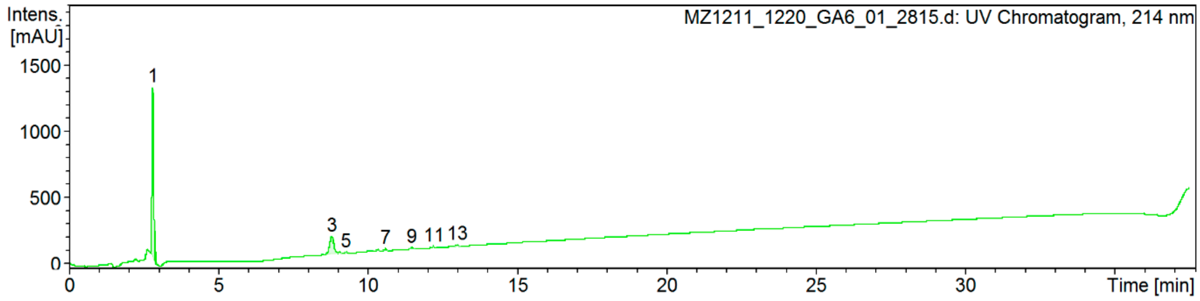

Figure S64. HPLC of peptide **27**.

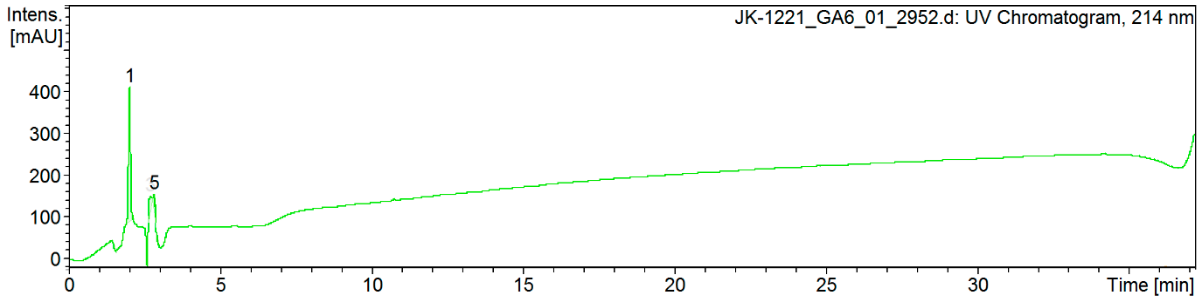

Figure S65. HPLC of peptide **28**.

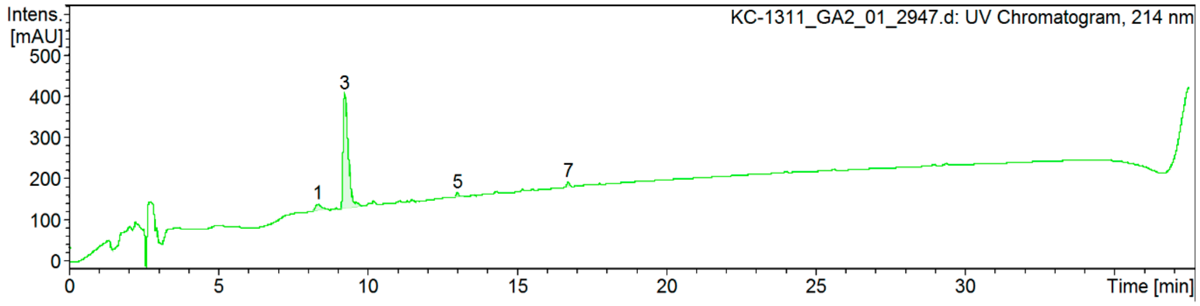

Figure S66. HPLC of peptide **29**.

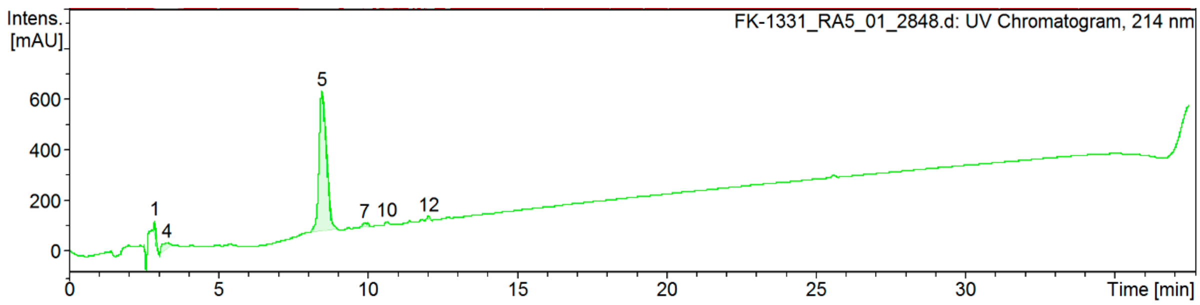

Figure S67. HPLC of peptide **30**.

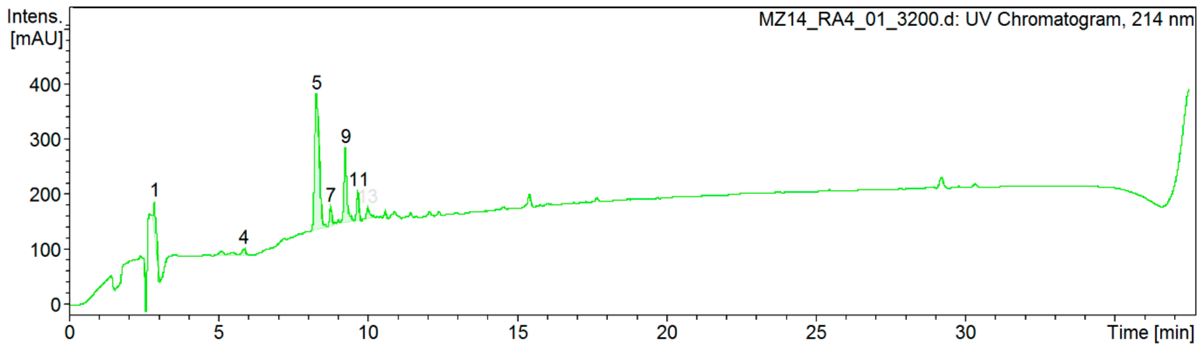

Figure S68. HPLC of peptide **31**.

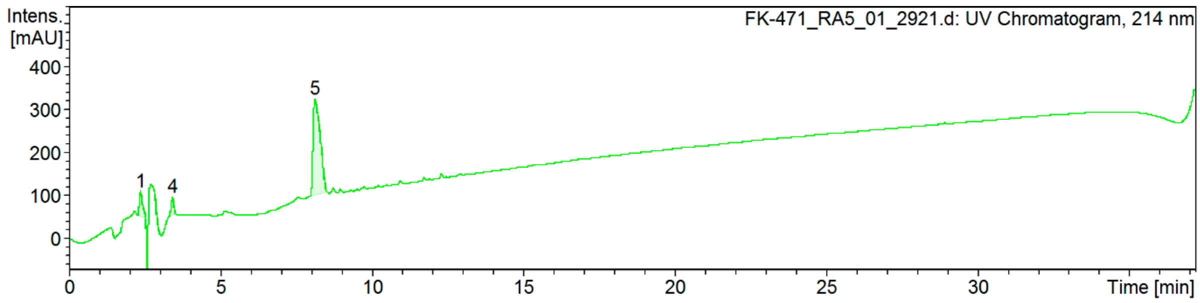

Figure S69. HPLC of peptide **32**.

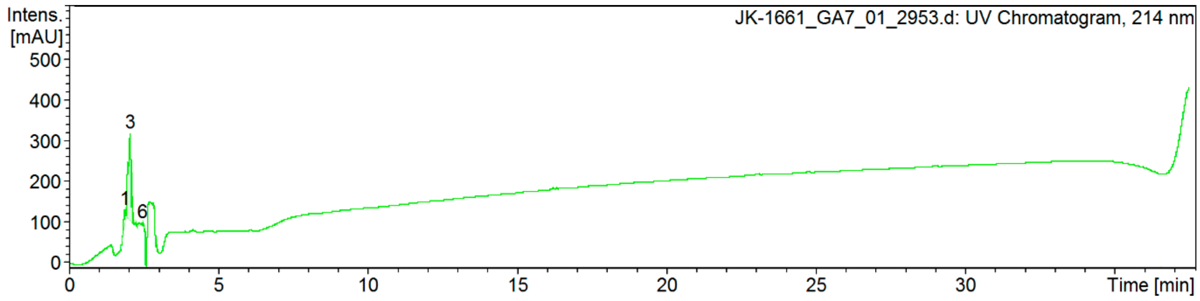

Figure S70. HPLC of peptide **33**.

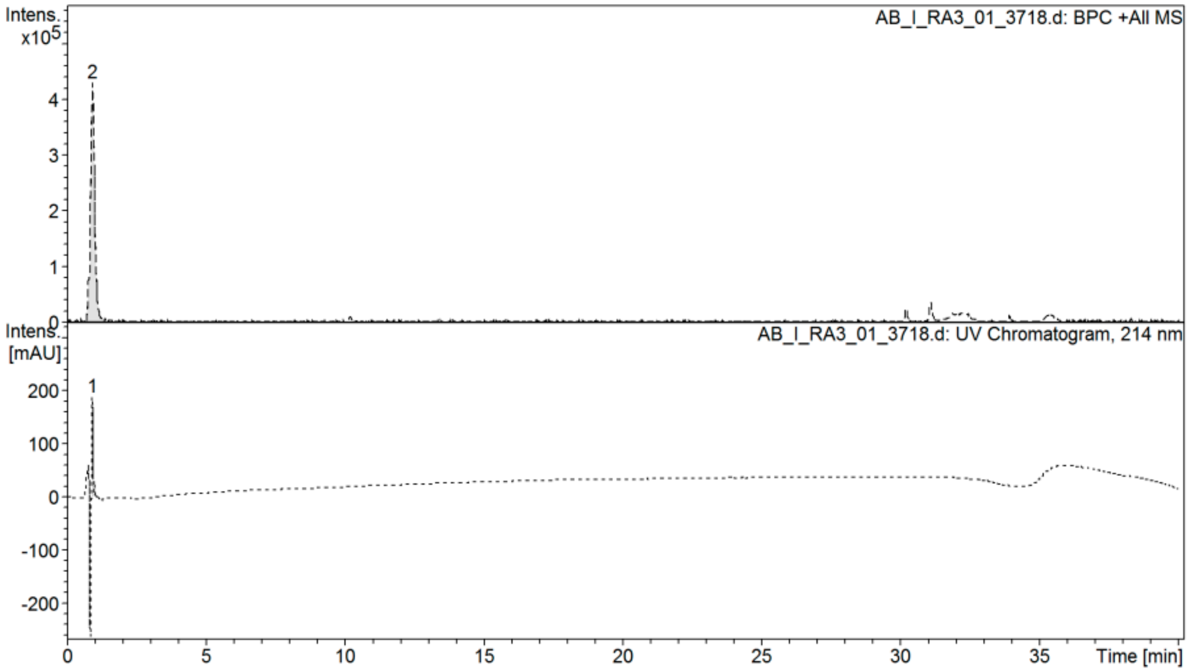

Figure S71. HPLC of peptide **2.1**.

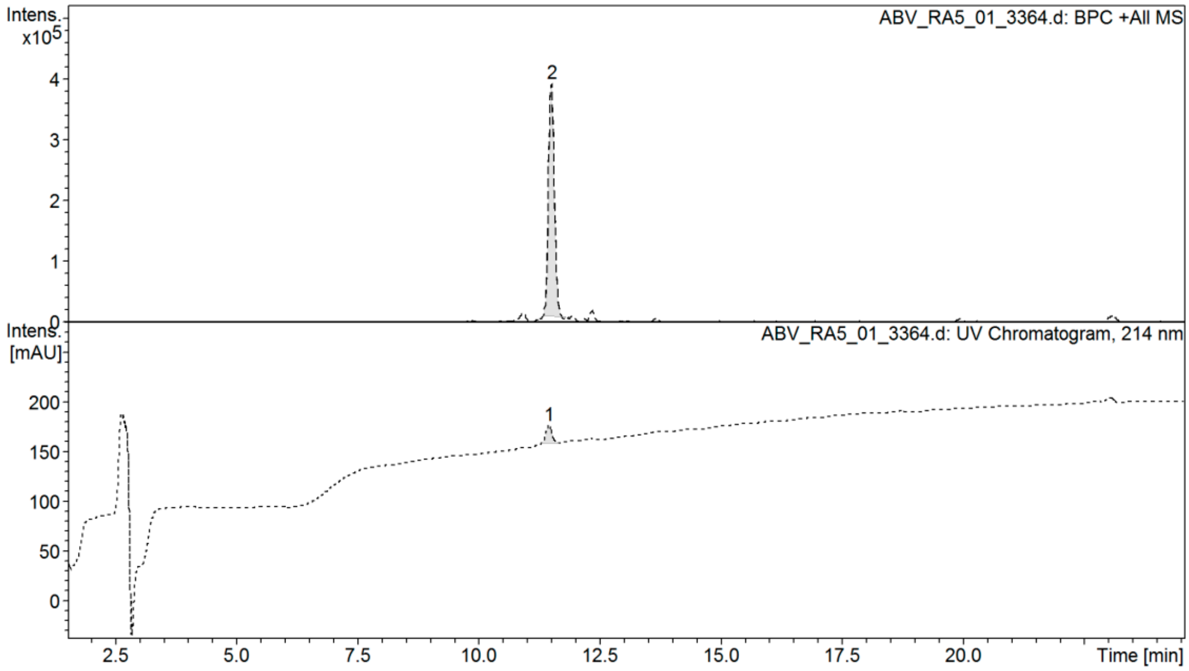

Figure S72. HPLC of peptide **2.2**.

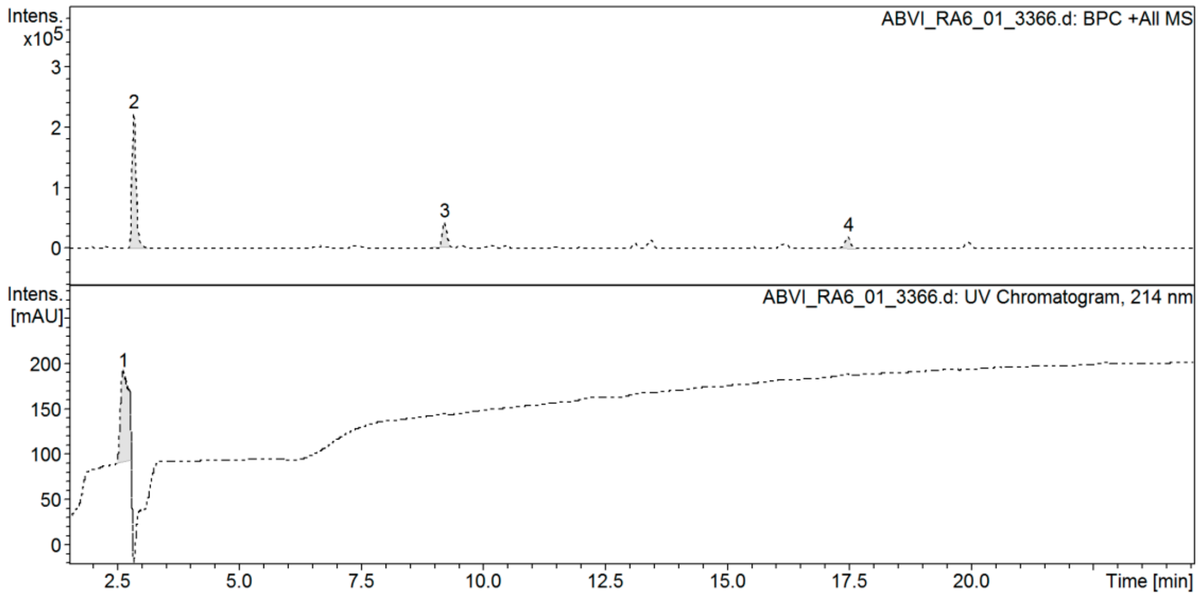

Figure S73. HPLC of peptide **2.3**.

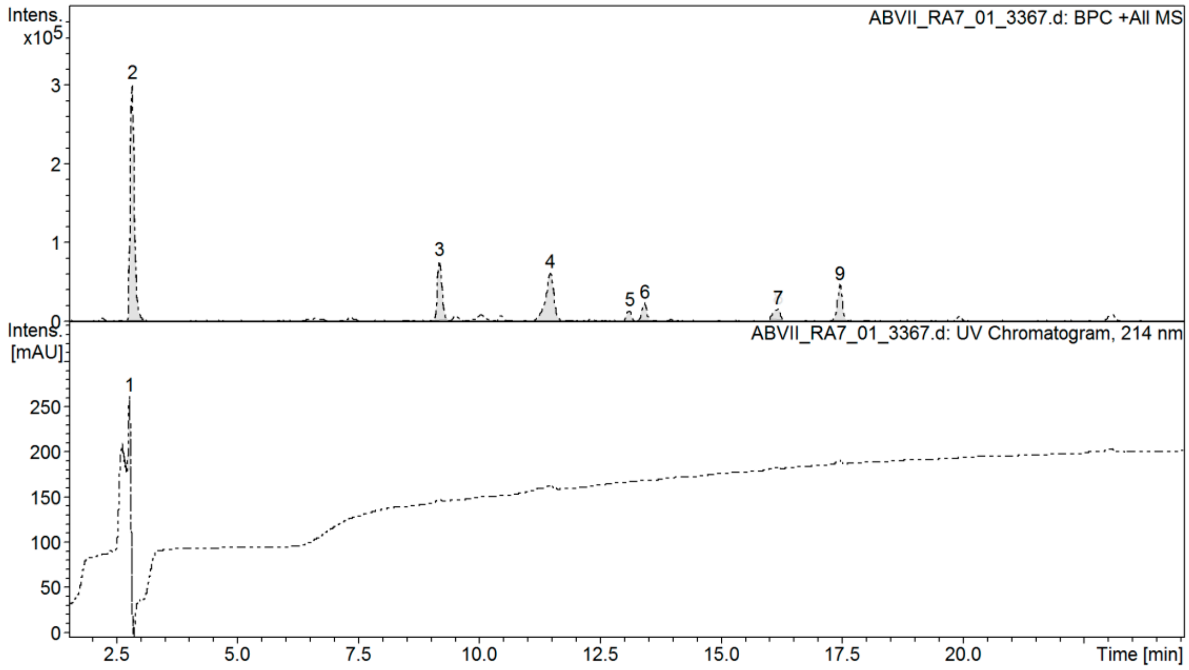

Figure S74. HPLC of peptide **2.4**.

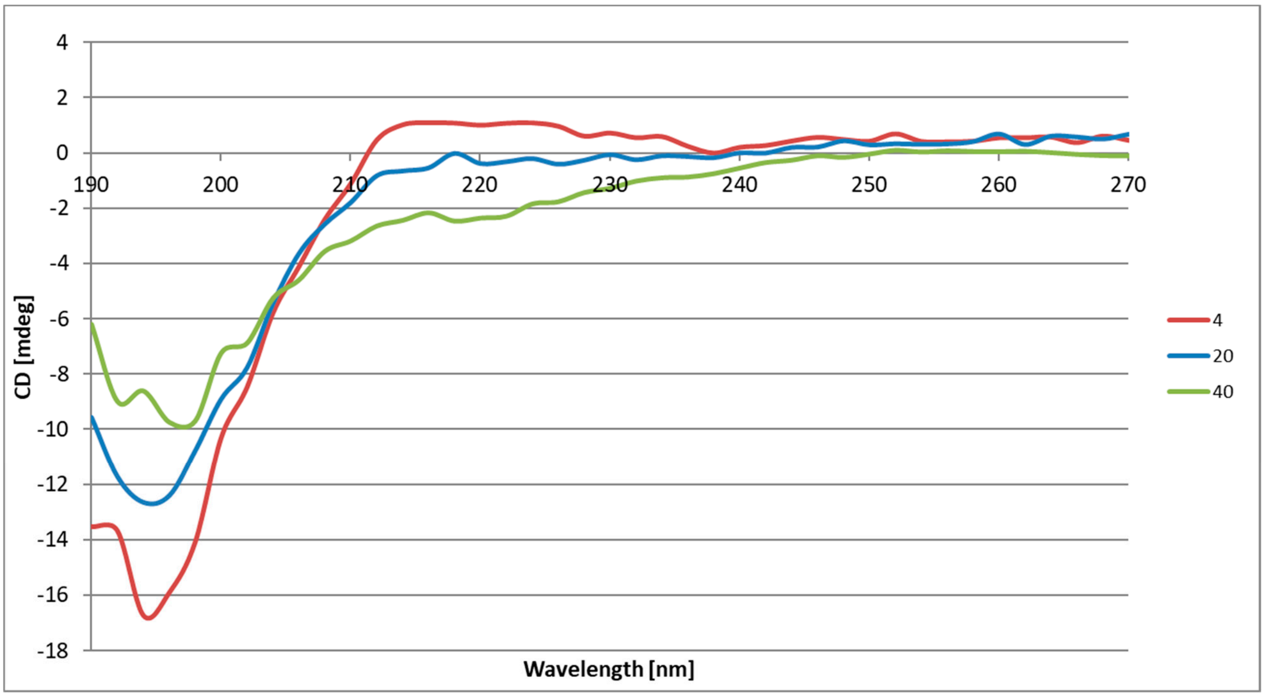

Figure S75. CD spectra of peptide **1**.

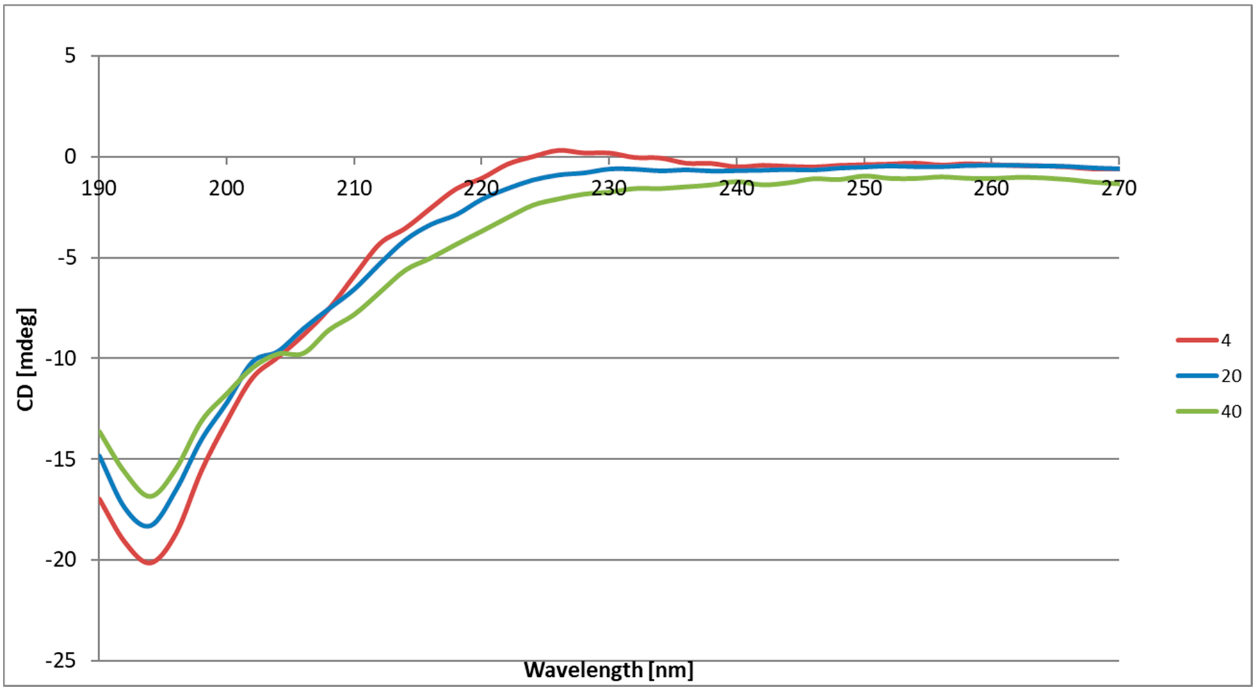

Figure S76. CD spectra of peptide **3**.

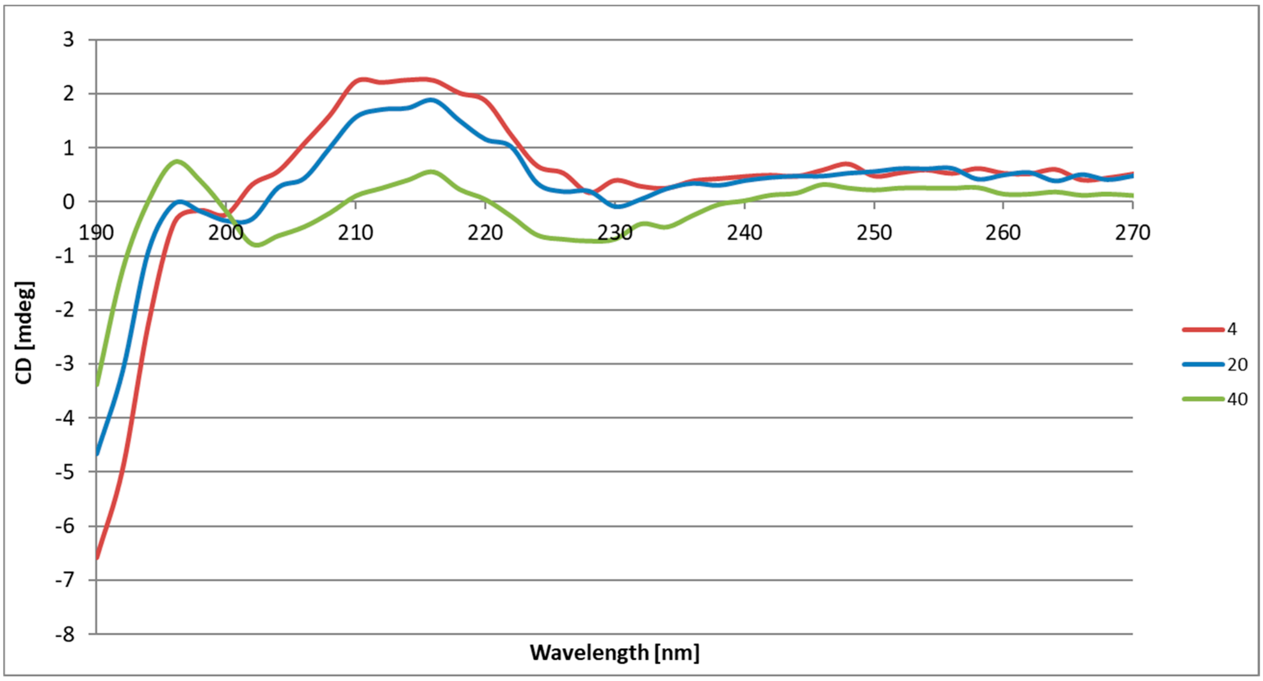

Figure S77. CD spectra of peptide **9**.

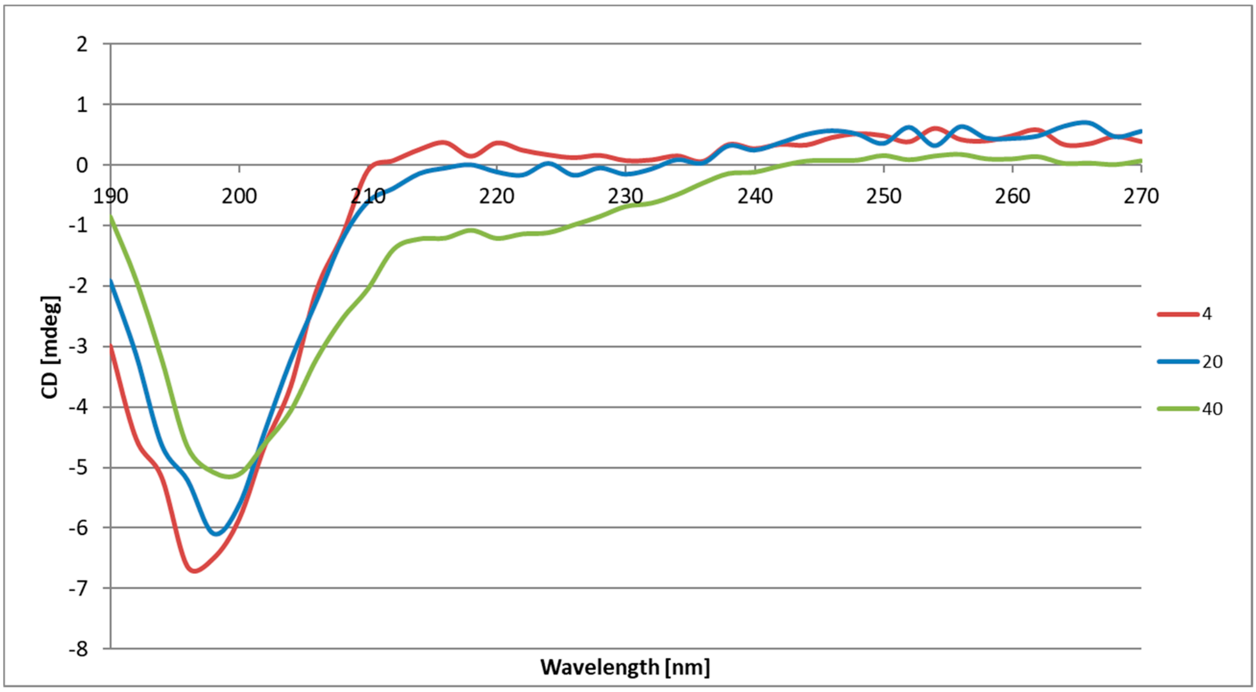

Figure S78. CD spectra of peptide **10**.

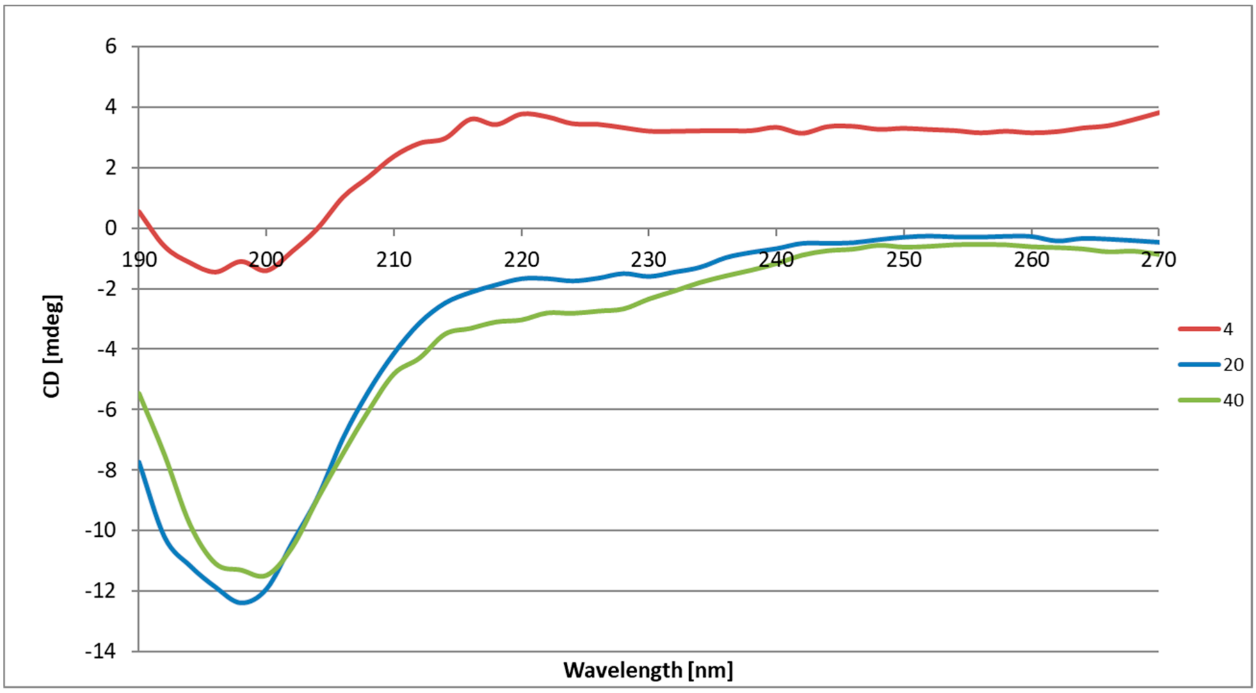

Figure S79. CD spectra of peptide **11**.

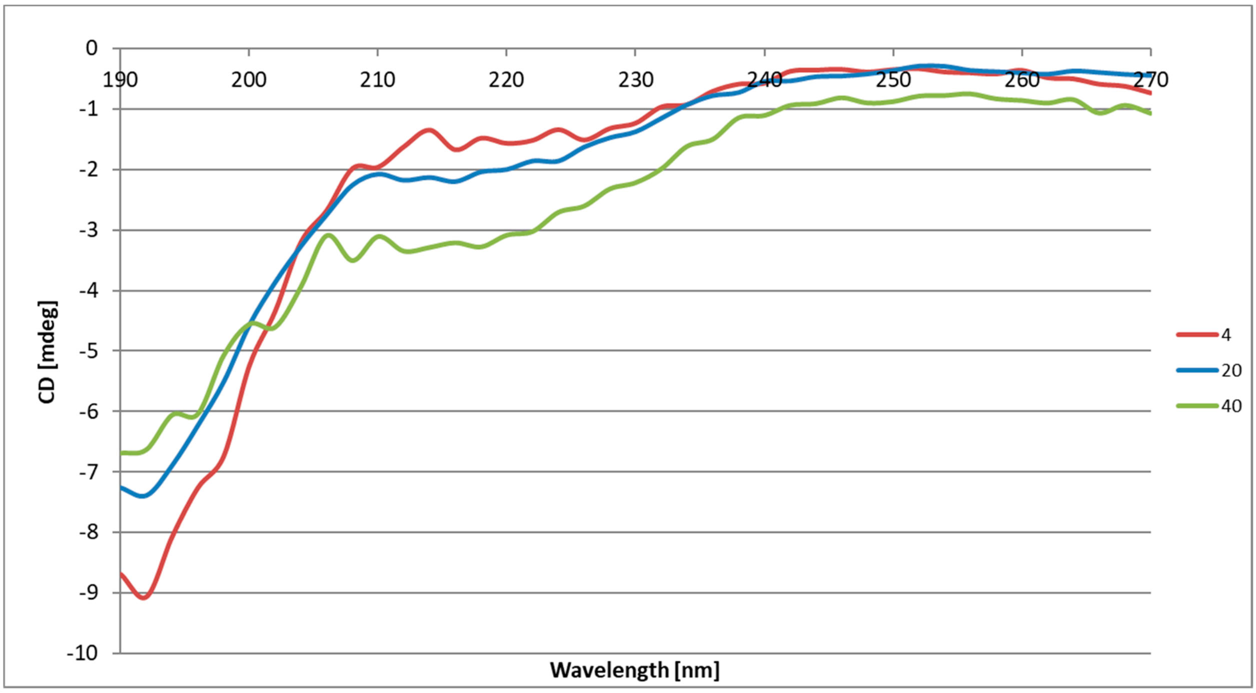

Figure S80. CD spectra of peptide **16**.

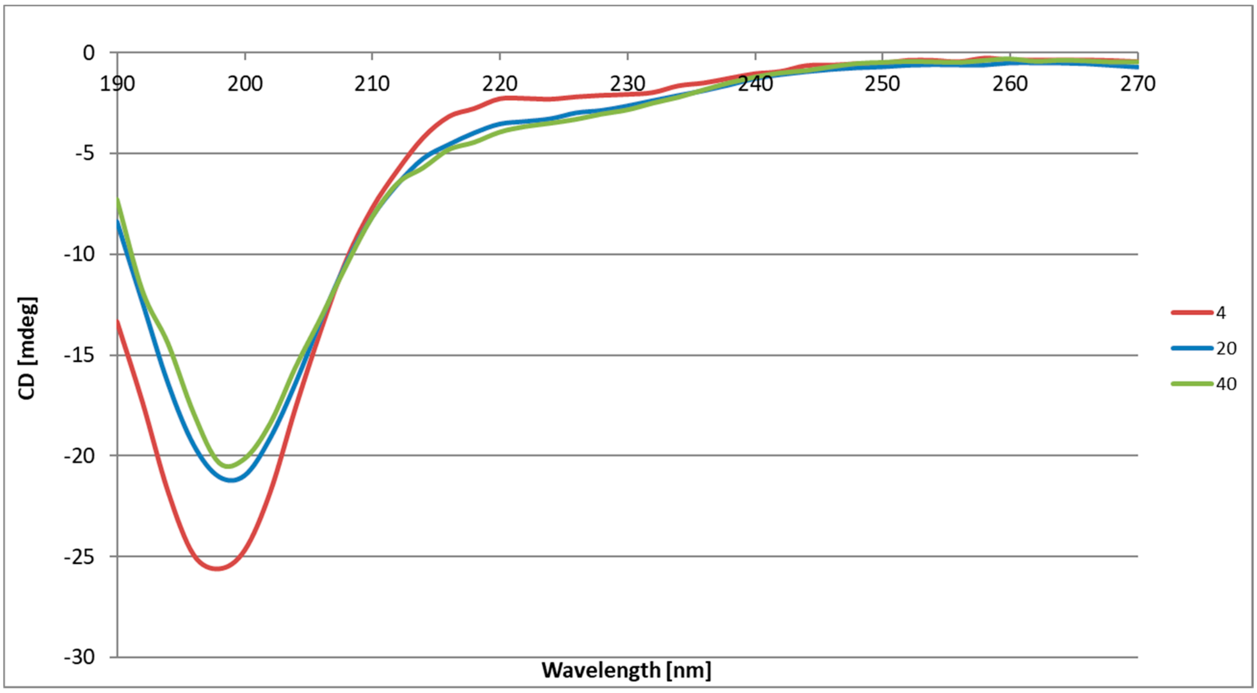

Figure S81. CD spectra of peptide **19**.

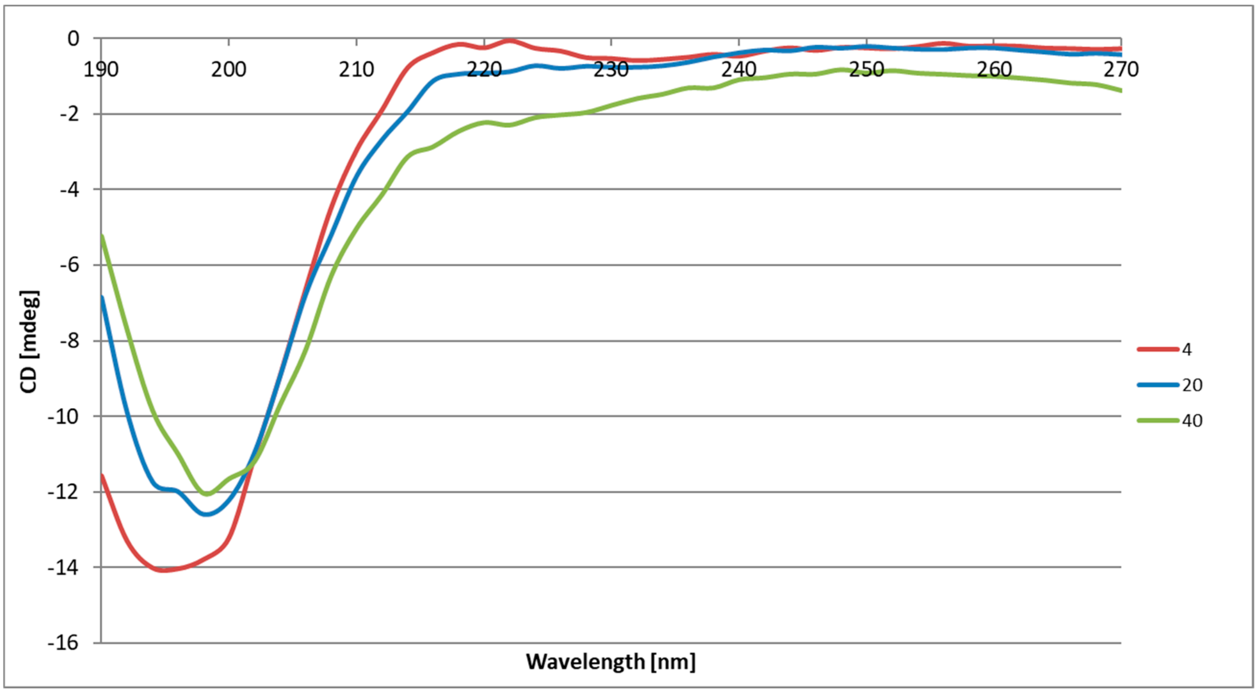

Figure S82. CD spectra of peptide **20**.

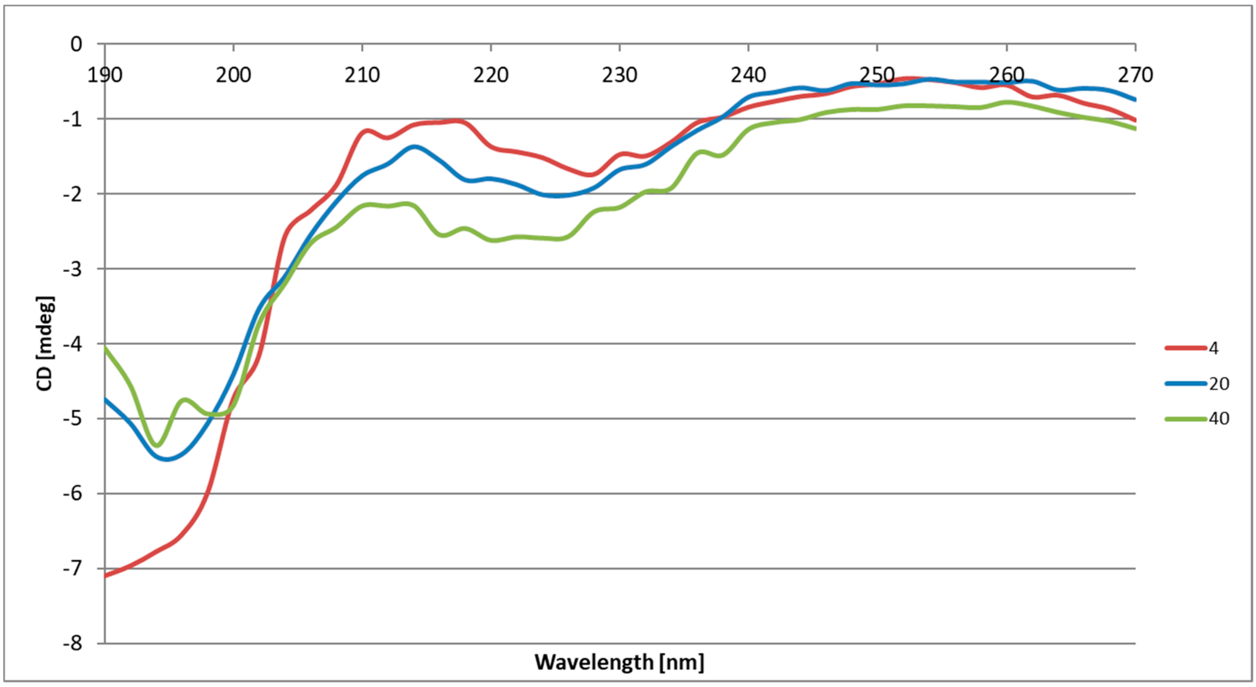

Figure S83. CD spectra of peptide **21**.

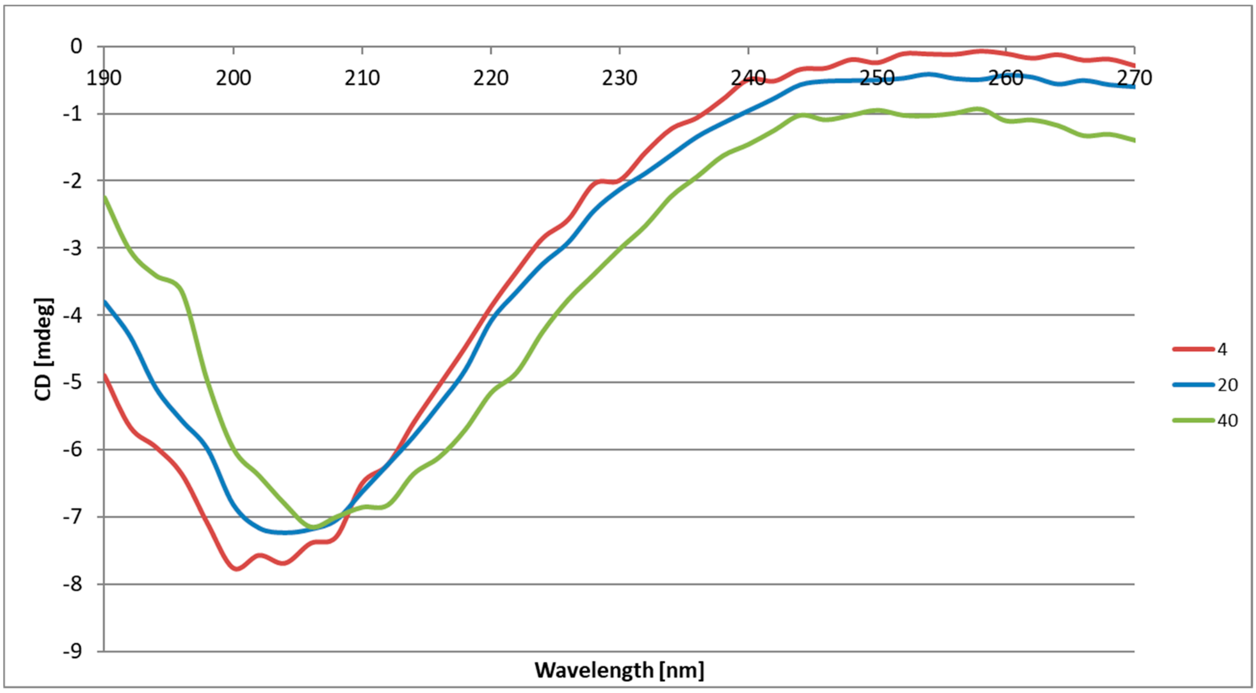

Figure S84. CD spectra of peptide **22**.

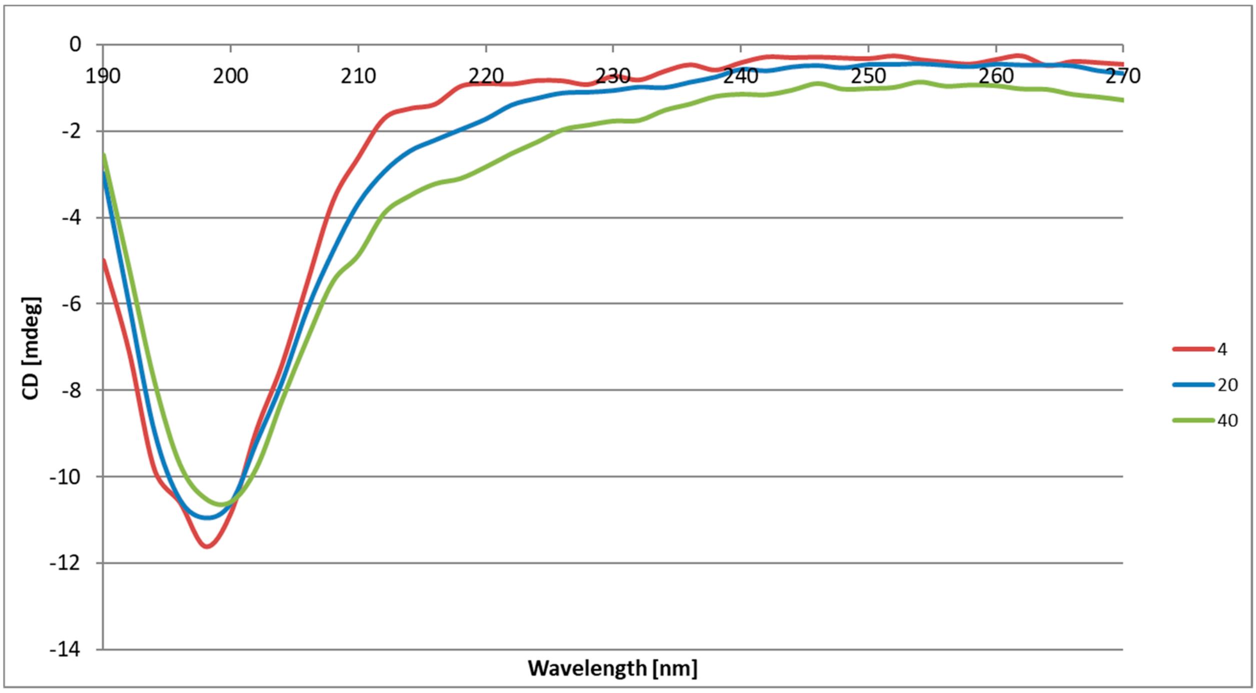

Figure S85. CD spectra of peptide **23**.

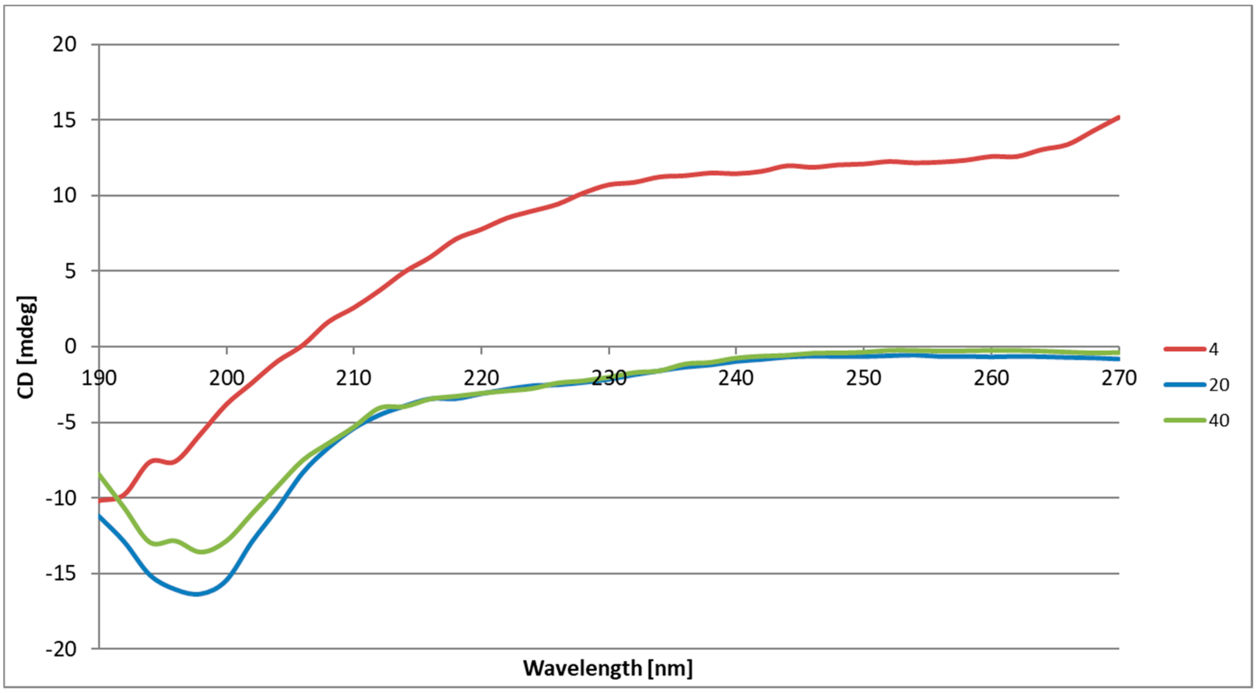

Figure S86. CD spectra of peptide **24**.

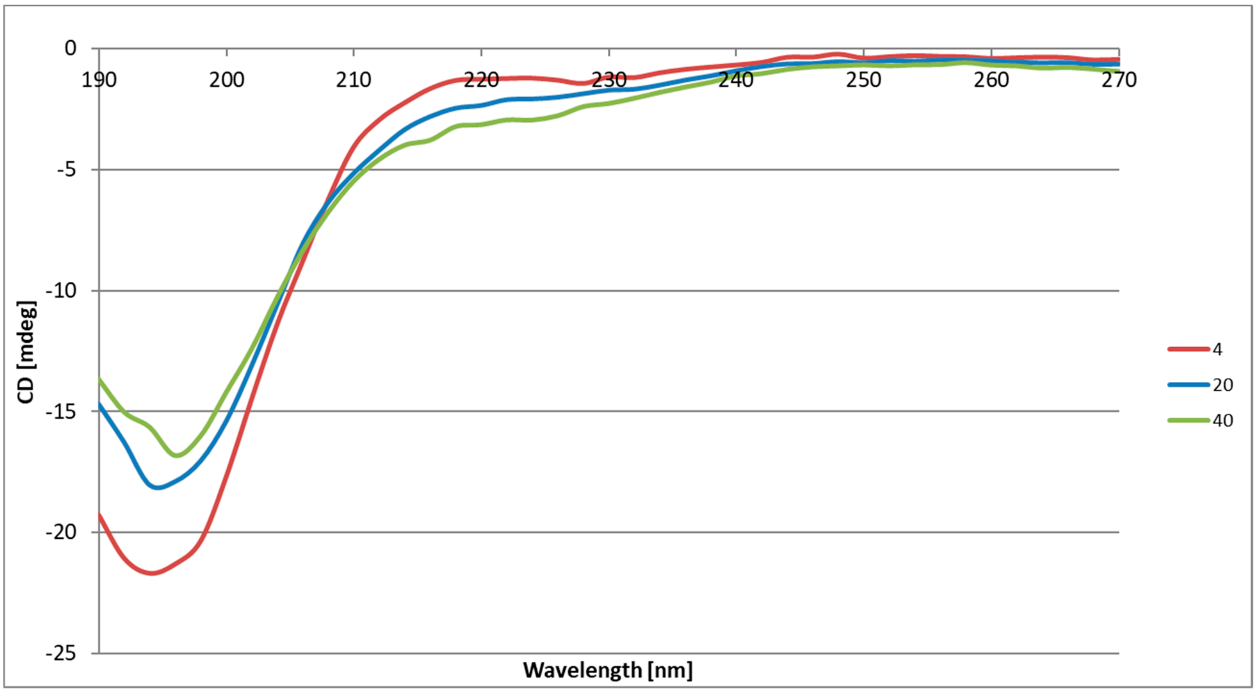

Figure S87. CD spectra of peptide **27**.

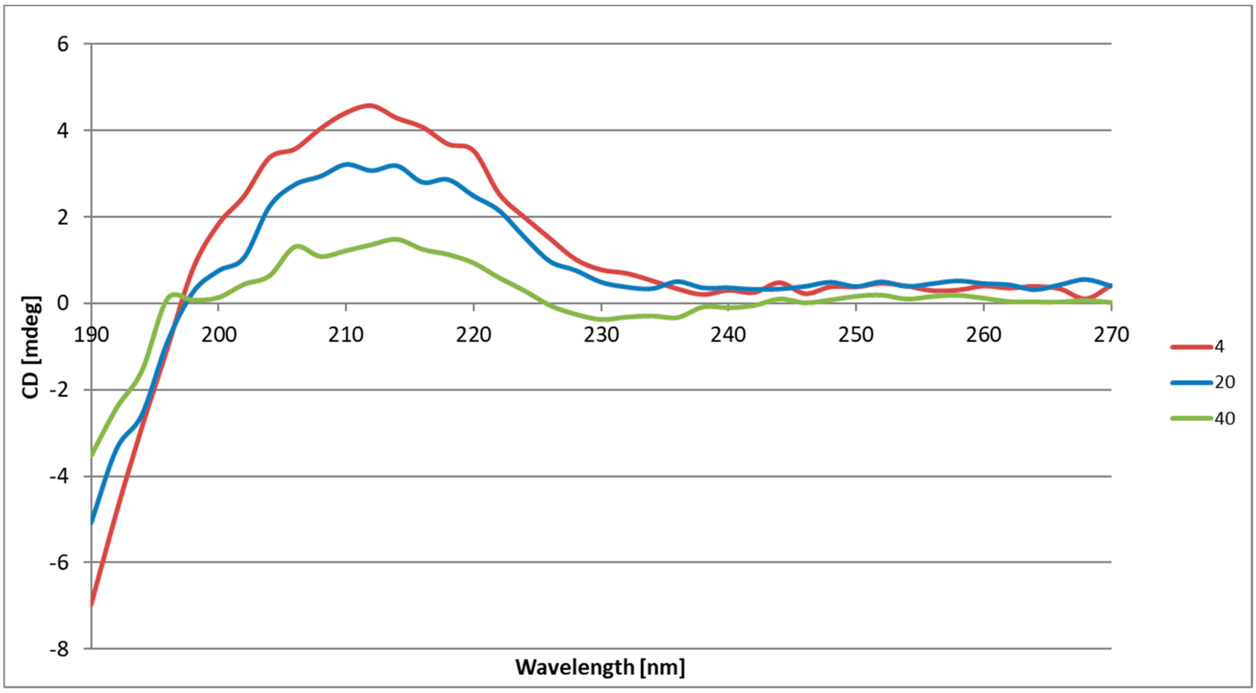

Figure S88. CD spectra of peptide **29**.

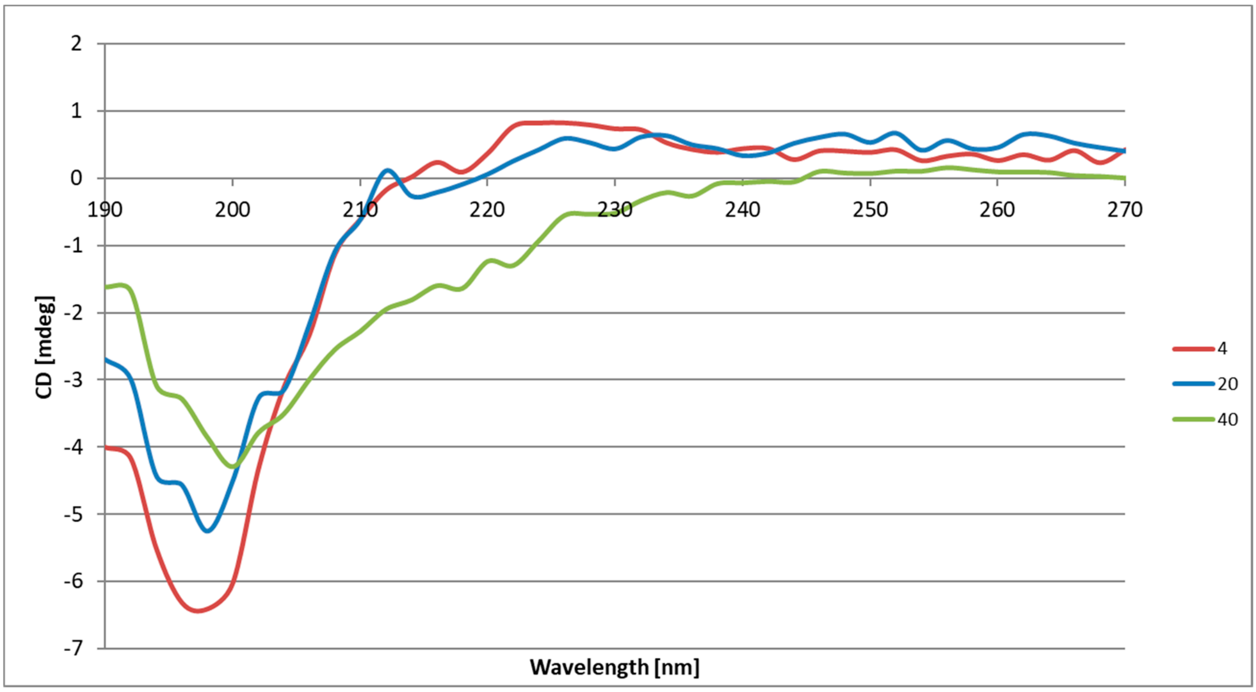

Figure S89. CD spectra of peptide **31**.

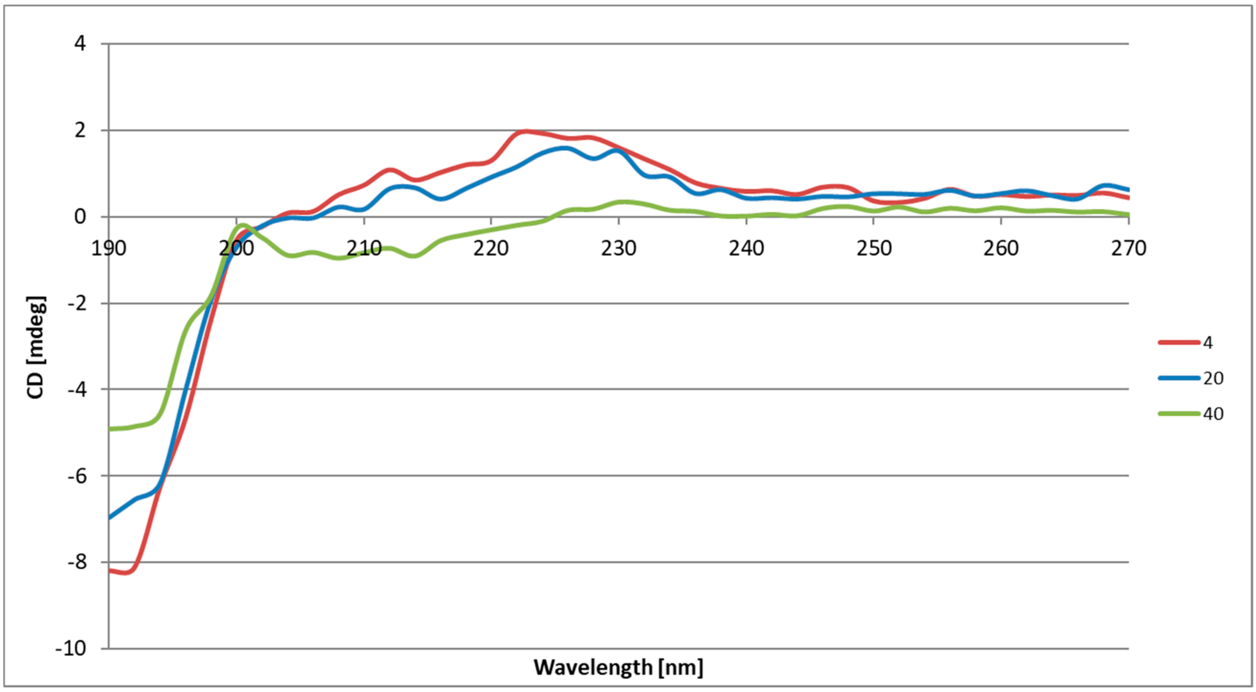

Figure S90. CD spectra of peptide **32**.

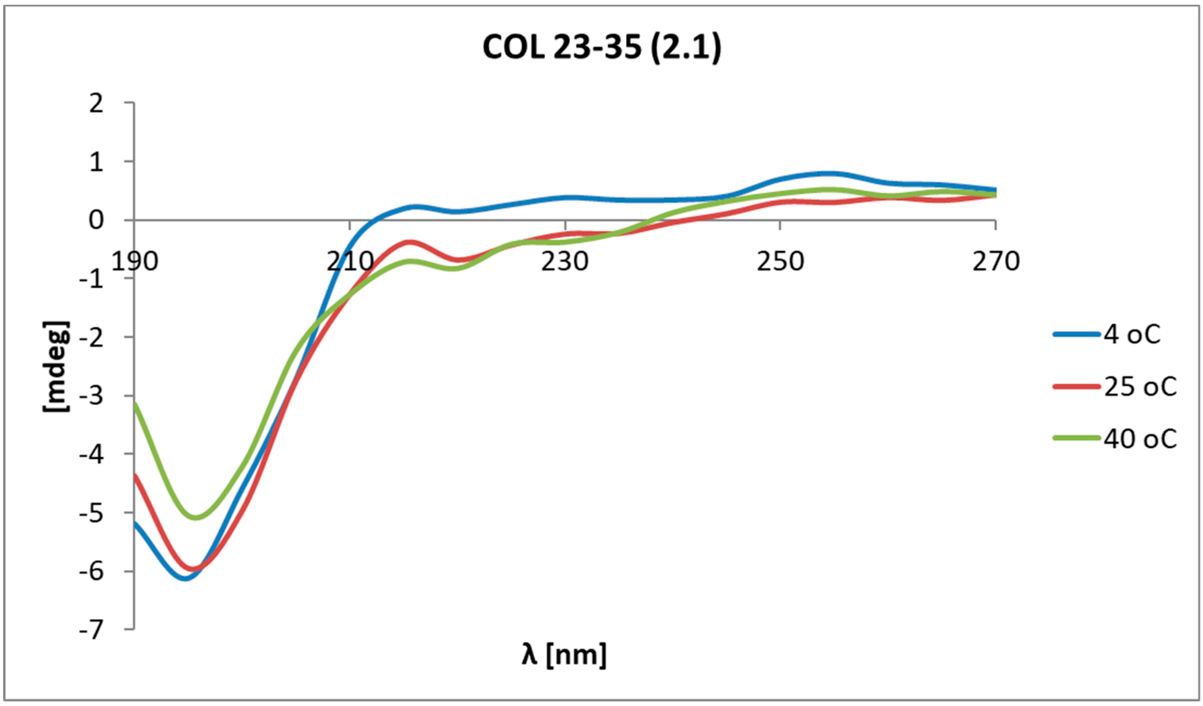

Figure S91. CD spectra of peptide **2.1**.
